# DFT‐assisted rational design of salan ligands for highly enantioselective Zr–salan‐catalyzed hydroxylation of cyclic *β*‐keto esters

**DOI:** 10.1002/smo2.70093

**Published:** 2026-08-27

**Authors:** Cunfei Ma, Kun Tang, Yuchen Jiang, Hongfei Zhu, Yufeng Wu, Yafeng Xing, Yakun Wang, Qilei Liu, Jian Du, Qingwei Meng

**Affiliations:** ^1^ Department of Pharmaceutical Engineering State Key Laboratory of Fine Chemicals Frontiers Science Center for Smart Materials Oriented Chemical Engineering School of Chemical Engineering Dalian University of Technology Dalian China; ^2^ School of Pharmacy Xinxiang Medical University Xinxiang Henan China

**Keywords:** asymmetric catalysis, cyclic *β*‐keto esters, DFT‐assisted, hydroxylation, Zr‐salan catalysts

## Abstract

In this work, a structurally simple, inexpensive, and readily accessible Zr–salan catalytic system was designed and developed with the aid of density functional theory (DFT) calculations. Density functional theory studies revealed that stereocontrol arises primarily from the steric shielding and noncovalent interactions of the salan ligand, rather than diaryl structural motifs, enabling the rational design of an economically efficient ligand framework. This strategy eliminates the reliance on Suzuki–Miyaura coupling by employing a commercially available sterically hindered ligand intermediate. The resulting Zr–salan catalyst exhibits excellent asymmetric catalytic performance, delivering yields of up to 99% and enantiomeric excesses (ee) of 99% across a broad substrate scope of 44 examples, including *β*‐keto esters derived from 1‐indanone and 1‐tetralone. Further DFT investigations indicate that weak noncovalent interactions (C–H···C–H) between the Zr catalyst and the substrate play a crucial role in the reaction mechanism, providing insight into the origin of the high enantioselectivity. The synthetic practicality of this method was demonstrated through gram‐scale reactions, catalyst recyclability over five cycles, and product derivatization.

## Graphical abstract

Guided by DFT calculations, the design of novel C1‐symmetric salan ligands without the involvement of Suzuki coupling was achieved, and the preparation of *α*‐hydroxy‐β‐ketoesters in 99% yield and 99% ee was realized under low catalyst loading.

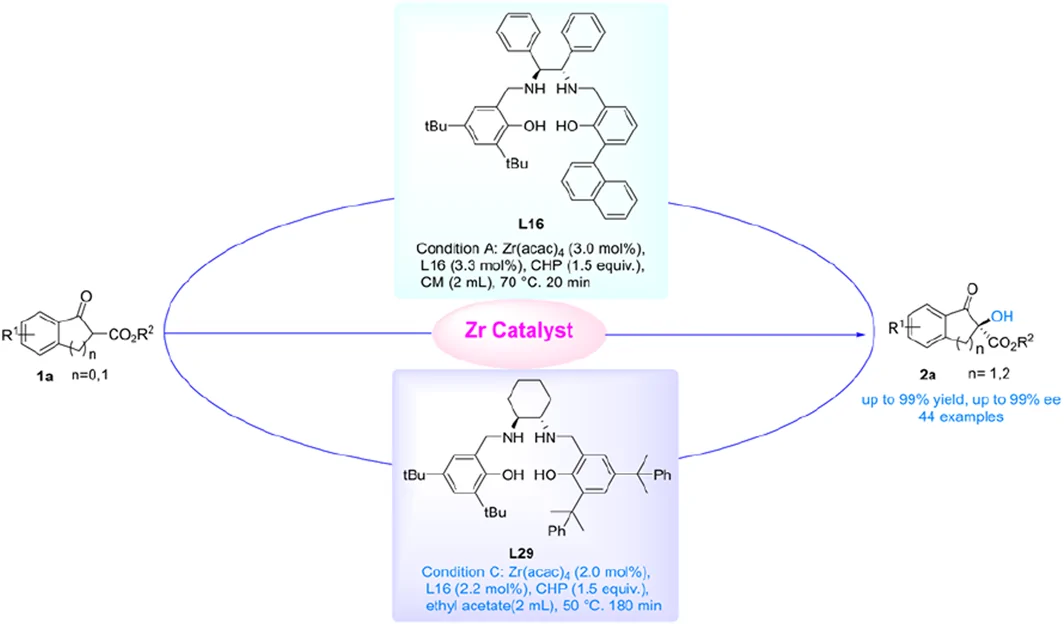

## 1 INTRODUCTION

**FIGURE 1:**
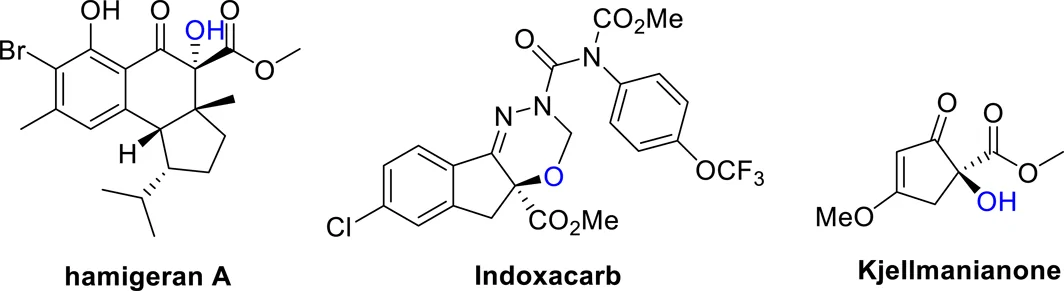
Selected bioactive molecules and agrochemicals containing a chiral *α*‐hydroxy‐β‐ketoester structure.

Asymmetric hydroxylation represents one of the most efficient and broadly applicable strategies for constructing chiral hydroxyl units in organic synthesis (Figure 1).[^1^, ^2^, ^3^] Chiral 1,3‐dicarbonyl derivatives bearing hydroxyl groups—particularly *β*‐keto esters derived from 1‐indanone and 1‐tetralone scaffolds—are prevalent motifs in the core structures of natural products and chiral agrochemicals, highlighting their synthetic relevance. The chiral hydroxyl group also provides a versatile platform for downstream functionalizations, enabling the formation of additional stereogenic centers and facilitating highly efficient transformations into chiral fluorine‐containing units.^[4]^ Accordingly, the development of enantioselective asymmetric hydroxylation methods for 1,3‐dicarbonyl compounds remains an important and active area of research in synthetic chemistry.

**SCHEME 1:**
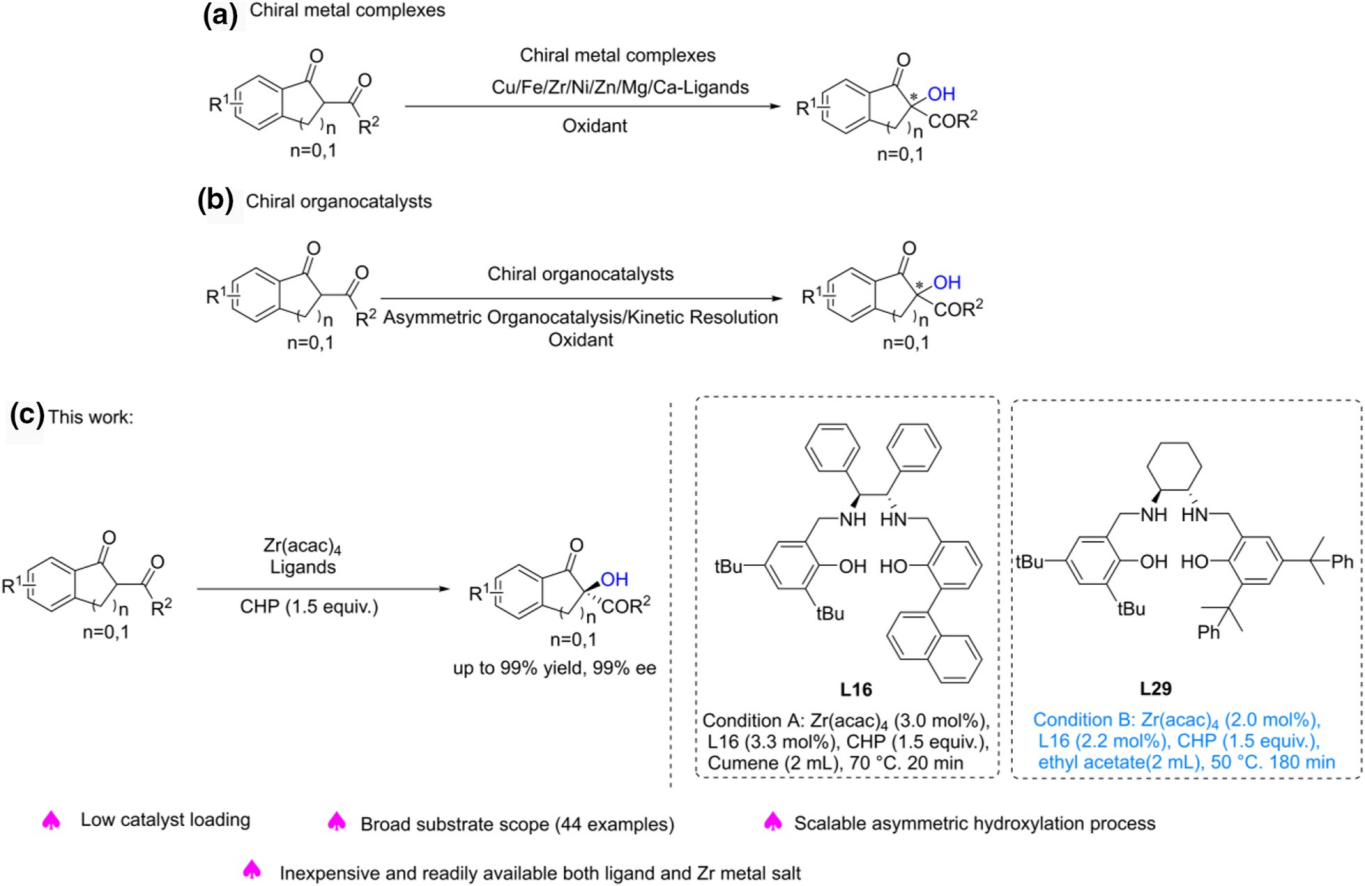
Catalytic asymmetric synthesis of chiral *α*‐hydroxy‐*β*‐ketoesters.

The asymmetric *α*‐hydroxylation of *β*‐keto esters has been pursued using chiral metal complexes,[^5^, ^6^, ^7^, ^8^, ^9^, ^10^, ^11^] organocatalysts,[^12^, ^13^, ^14^, ^15^, ^16^, ^17^] and enzymatic systems.^[18]^ Whereas organocatalytic methods offer valuable complements, their scope is largely restricted to bulky ester groups (e.g.., tBu and Ad), with chiral phosphoric acids remaining notable exceptions for less hindered substrates. In contrast, chiral metal complexes—such as those derived from Fe(III)–salan,^[7]^ Ni(II)–bis(oxazoline),^[8]^ Zr(IV)–salan,[^9^, ^10^] Zn(II)–SPANbox,^[5]^ and Mg(II)–N, N′‐dioxide ligands^[6]^—generally exhibit superior reactivity and stereocontrol toward *β‐*ketoesters bearing bulky ester groups, with the exception of the Zr(IV)–salan and Mg(II)–N,N′‐dioxide catalytic systems (Scheme 1).

However, their application to *β*‐keto esters based on 1‐indanone and 1‐tetralone scaffolds remains challenging. For instance, Mg(II)–N, N′‐dioxide catalysts exhibit good performance with 1‐tetralones but suffer a drastic drop in enantioselectivity for small‐ester 1‐indanones (e.g., 26% ee).^[6]^ Conversely, Zr(IV)–salan systems achieve high ee with the latter but are ineffective for 1‐tetralones (e.g., 9% ee).^[9]^ These limitations highlight three persistent constraints in the field: (i) Existing catalytic systems also suffer from poor recyclability, as catalytic performance declines significantly in the second cycle, hindering efficient reuse. (ii) Current systems lack broad substrate generality. For example, our previous study showed that the asymmetric hydroxylation of 1‐tetralone‐derived *β*‐ketoesters is efficient only with the Hf(IV)–salan system, while the Zr(IV)–salan system performs poorly. No reported system can simultaneously accommodate both 1‐indanone‐ and 1‐tetralone‐derived *β*‐ketoesters, and (iii) dependence on expensive synthetically demanding ligands for small‐ester substrates. Most privileged ligands rely on biaryl backbones constructed via transition‐metal‐catalyzed cross‐couplings (e.g., Suzuki–Miyaura, involving the introduction of aryl groups catalyzed by precious palladium), including our recently designed salan ligands, which significantly increase catalyst cost and hamper industrial application.[^9^, ^10^, ^19^, ^20^] These challenges underscore the urgent need for readily accessible and cost‐effective chiral ligands that enable asymmetric hydroxylation across diverse 1‐indanone and 1‐tetralone derivatives and various ester substituents.

The asymmetric catalytic performance of metal complexes is largely dictated by the electronic and steric properties of the coordinated chiral ligands, which define the stereochemical environment at the metal center.^[21]^ Expanding the utility of chiral ligands commonly involves modifying substituents within established chiral frameworks to generate structurally related derivatives. With advances in organic and computational chemistry, density functional theory (DFT) calculations have become increasingly valuable for guiding the optimization of reactions.[^22^, ^23^] Integrating mechanistic insights with DFT analysis provides a more rational and efficient approach to tuning chiral ligand structures than empirical methods.[^23^, ^24^, ^25^] Based on literature precedents, efforts have focused on the structural refinement of salan ligands to identify candidates suitable for highly efficient asymmetric hydroxylation of *β*‐keto esters. A readily accessible and cost‐effective C1‐symmetric salan ligand was subsequently developed through a mechanistic and DFT‐assisted design strategy, eliminating the need for Suzuki–Miyaura coupling. The resulting Zr–salan catalytic system enables the asymmetric hydroxylation of *β*‐keto esters derived from 1‐indanone and 1‐tetralone, affording the corresponding tertiary alcohols in high yields and excellent enantioselectivities (up to 99% yield, 99% ee), while featuring low catalyst loading (2 mol%) and broad substrate scope (44 examples).

## 2 RESULTS AND DISCUSSION

A sterically demanding C1‐symmetric salan ligand library was prepared from 3,5‐di‐tert‐butylsalicylaldehyde via condensation with (1S, 2S)‐diphenylethylenediamine and subsequent diversification with substituted salicylaldehydes. Using 5‐chloro‐1‐indanone methyl ester (**1a**) as a model substrate, the **L1**–Zr (acac)_4_ system in CHP/isopropylbenzene delivered good performance (98% yield, 97% ee), establishing isopropylbenzene as the solvent of choice (Table 1, entries 2–10, See Supporting Information S1 for details).

**TABLE 1:** Optimization of the reaction conditions^a^.

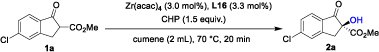			
Entry	Variation from the standard conditions	Yield^b^ (%)	ee^c^ (%)
1	None	99	99
2	Mn (OAc)_2_	43	12
3	Co (OAc)_2_	18	−18
4	Ni (acac)_2_	76	15
5	Toluene as solvent	98	97
6	*n*‐hexane as solvent	98	95
7	Acetone as solvent	88	92
8	Cyclohexane as solvent	98	95
9	MTBE as solvent	89	91
10	Acetonitrile as solvent	98	88
11	3 mol% L16/L21 as ligand	99	99
12	2 mol% L16/L21 as ligand	98	98
13	TBHP instead of CHP	97	97
14	30°C/40°C/50°C/60°C instead of 70°C	99	99
15	80°C instead of 70°C	98	98

Abbreviations: EA, ethyl acetate; PE, petroleum ether, boiling range 60–90°C.

^a^ Reaction conditions: **1a** (0.1 mmol), CHP (1.5 equiv.), Zr (acac)_4_ (3.3 mol%), **L16/L21** (3.3 mol%), cumene (2 mL), 70°C, 20 min.

^b^ The yields of isolated products.

^c^ Determined by HPLC on chiral stationary phase.

**SCHEME 2:**
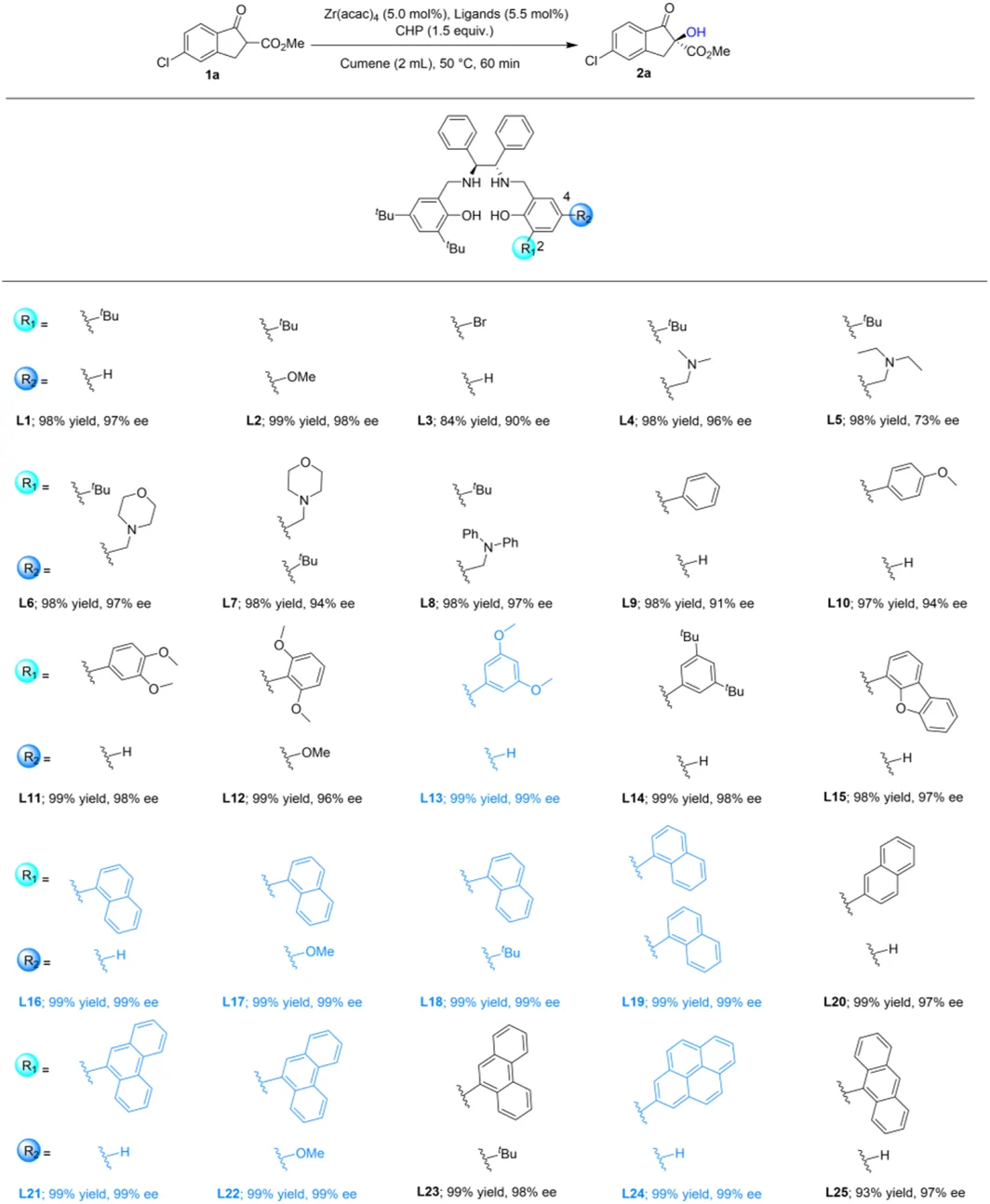
Salan ligand evaluation^*a*, *b*, *C*^. ^*a*^Reaction conditions: **1a** (0.1 mmol), CHP (1.5 equiv.), Zr (acac)_4_ (5.0 mol%), ligand (5.5 mol%), cumene (2 mL), 50°C, nitrogen. ^*b*^The yields of isolated products. ^*c*^Determined by HPLC on the chiral stationary phase.

Structure–activity analysis revealed that steric effects at the ligand 2‐position dominate catalytic behavior. L2 matched L1, whereas introducing Br at C2 (L3) significantly lowered activity and ee. Ligands with 2‐tert‐butyl and various 4‐N substituents (L4–L6, L8) gave modest improvements, while modification of the 2,4‐pattern (**L6 → L7**) underscored the importance of C2 steric demand. Aryl substitution at the 2‐position (**L9–L16**) enabled fine‐tuning of the steric bulk and conjugation. Smaller aryl groups reduced ee, whereas bulkier motifs (**L11–L12**) restored high selectivity. **L13** and **L16** were particularly effective (99% yield, 99% ee). Extended aromatic systems (**L17–L25**) generally maintained excellent performance, with minimal influence from 4‐electron‐donating groups and only excessive bulk (e.g., 9‐anthryl) slightly reducing ee (Scheme 2).

Catalyst‐loading studies identified **L16** and **L21** as the only ligands sustaining 99% ee at 3 mol%. Considering the selectivity, simplicity, and synthetic accessibility, **L16** was chosen for optimization. Under **L16**–Zr (acac)_4_ (1.1:1), increasing the temperature to 70°C shortened the reaction time to 20 min without compromising 99% yield and 99% ee; 80°C reduced the time to 10 min with a slight drop to 98% ee. Optimal conditions were established as 3 mol% **L16**–Zr (acac)_4_, CHP, isopropylbenzene, 70°C, 20 min (Table 1, entries 11–15, See Supporting Information S1 for details).

**SCHEME 3:**
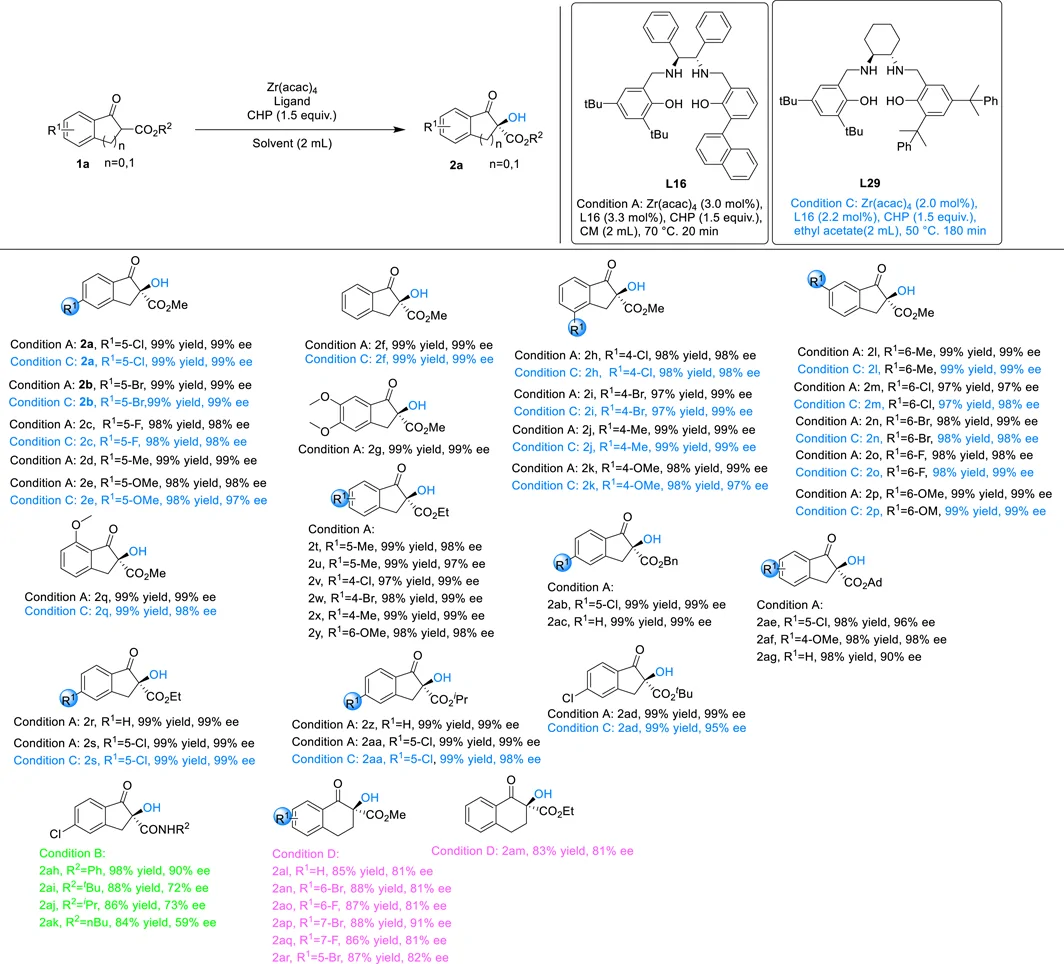
Substrate Scope of *β*‐ketoesters. ^*a*^Unless otherwise noted, all reactions were carried out with Condition A: Zr (acac)_4_/**L16** (3.0 mol%), **1a** (0.1 mmol, 1.0 equiv.), CHP (0.15 mmol, 1.5 equiv.) in cumene at 70°C for 20 min. ^*b*^Condition B: Zr (acac)_4_/**L16** (5.0 mol%), at 50°C for 60 min. ^*c*^Condition C: Zr (acac)_4_/**L29** (2.0 mol%), in ethyl acetate EA at 50°C for 180 min. ^*d*^Condition D: Zr (acac)_4_/**L29** (5.0 mol%), in cumene at 15°C for 24 h. The ee values were determined by HPLC analysis using a chiral column Chiralce OD‐H, AD‐H, and AS‐H.

With the optimized conditions established, the substrate scope was evaluated systematically. *β*‐Ketoesters derived from 1‐indanone were first examined to assess the influence of aromatic substitution. Substrates bearing electron‐withdrawing halogens at the C5, C4, or C6 positions (5‐Cl/Br/F; 4‐Cl/Br; 6‐Cl/Br/F) were well‐tolerated, affording the corresponding hydroxylated products (**2a–2c**, **2h–2i**, **2m–2o**) in excellent yields (98%–99%) and enantioselectivities (98%–99% ee). Under conditions A, electron‐donating substituents—including C5‐Me/OMe, C5/C6‐OMe, C4‐Me/OMe, C6‐Me/OMe, the unsubstituted analog (**1f**), and the C7‐OMe derivative—were also compatible, providing products (**2d–2g**, **2j–2l**, **2p–2q**) in consistently high yields (98%–99%) and ee values (98%–99%), regardless of substitution pattern. With the ester moiety fixed as ethyl, additional aromatic variants were evaluated to probe electronic effects. Unsubstituted (**1r**), electron‐deficient (5‐Cl, 4‐Cl/Br), and methyl‐substituted (C4, C5, C6) substrates all reacted efficiently. Aside from the C6‐methyl substrate (**2u**, 97% ee), the hydroxylated products (**2r–2t**, **2v–2y**) were obtained in 97%–99% yields with 97%–99% ee. Steric effects in the ester group were then assessed using isopropyl (**1z**, **1aa**), benzyl (**1ab**, **1ac**), tert‐butyl (**1ad**), and 1‐adamantyl (**1ae–1ag**) esters. Isopropyl esters yielded **2z** and **2aa** with excellent enantioselectivity (99% ee). Benzyl and tert‐butyl esters also showed outstanding performance (**2ab–2ad**, 99% yield, 99% ee). For the more sterically hindered 1‐adamantyl esters, enantioselectivity varied (90%–98% ee) depending on the aromatic substituent. These data indicate broad structural tolerance of the catalytic system toward the ester moiety, a feature not previously documented. The method was further applied to *β*‐ketoamides, which present greater challenges due to reduced *α*‐hydrogen acidity, particularly with alkyl‐substituted amides. Conventional methods typically require indirect access via asymmetric nitration followed by hydrolysis and reduction. Under conditions B (5 mol% catalyst, 50°C), direct asymmetric hydroxylation of *β*‐ketoamides bearing Ph, ^t^Bu, ^*i*^Pr, and n‐Bu groups proceeded smoothly, affording products (**2ah–2ak**) in moderate to good yields (72%–98%) with satisfactory enantioselectivities (59%–90% ee) (Scheme 3).

**FIGURE 2:**
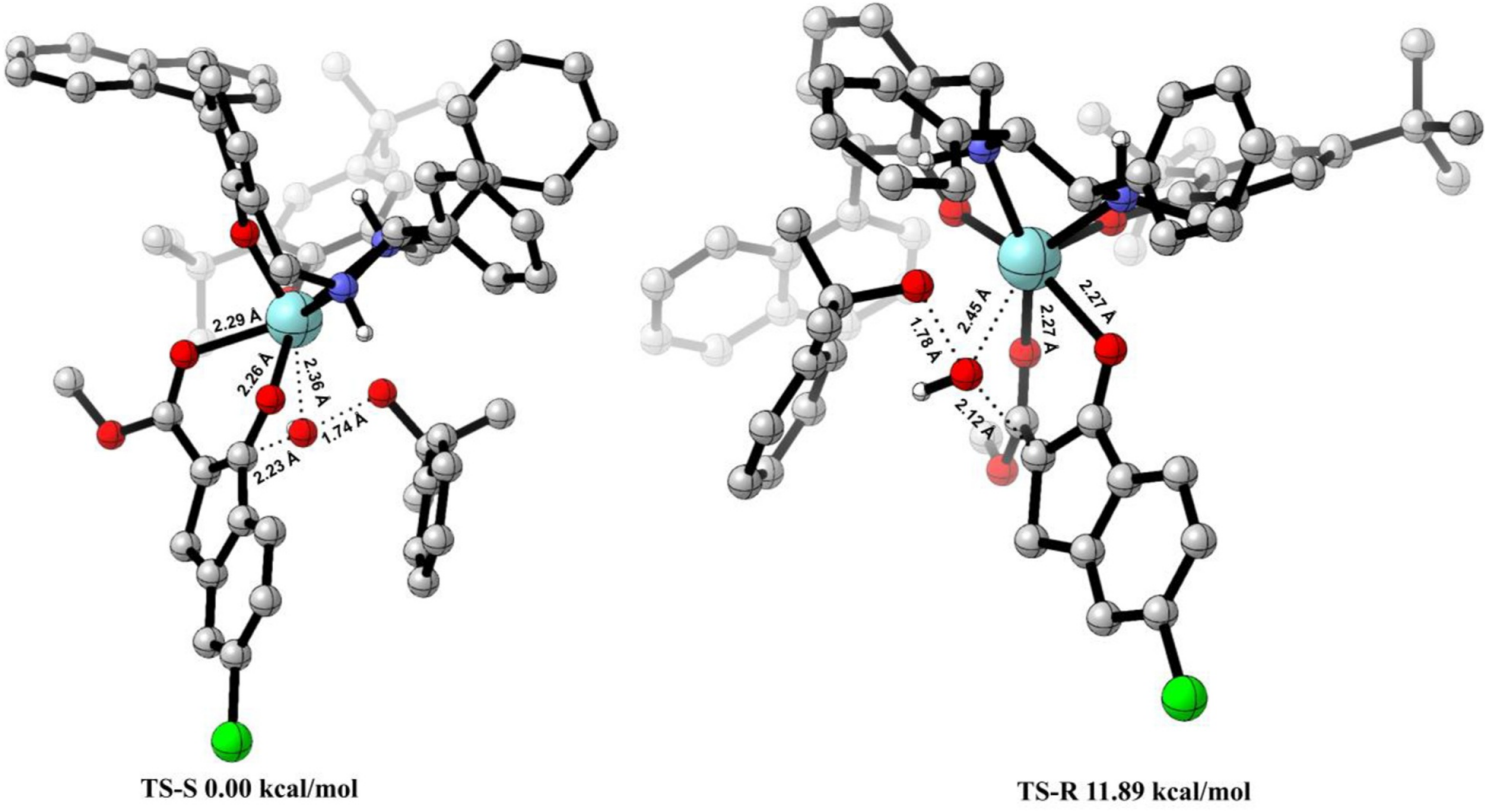
The optimized transition states and the relative Gibbs‐free energies (in kcal/mol) for the most stable one (**L16**). Bond distances are given in Å.

To elucidate the active catalytic species, key intermediates were identified by high‐resolution mass spectrometry (HRMS), including [**L16**–Zr(acac)]^+^ (calcd m/z 849.3203; found 849.3216) and the substrate‐bound species [**L16**–Zr–**1a**]^+^ (calcd m/z 793.2919; found 793.2896). Density functional theory calculations then located the key transition states, revealing that the energy barrier for the *S*‐configured product (TS‐S) is 11.89 kcal/mol lower than that for the *R*‐configured product (TS‐R), in full agreement with the experimental stereoselectivity (Figure 2). Independent gradient model (IGMH) analysis was subsequently conducted to unravel the origin of stereocontrol.[^26^, ^27^, ^28^, ^29^] In TS‐S, weak noncovalent interactions—including C–H···C–H interactions between the substrate and the ligand's tert‐butyl groups, C–H···N–H hydrogen bonding with the oxidant (CHP), and π–π stacking between the substrate and CHP—were identified (See Supporting Information S1 for details). Combined DFT/IGMH analyses suggested a distinct role for the ligand moieties: the tert‐butyl group engages in weak interactions with the substrate, while the 1‐naphthyl group primarily provides steric shielding. These computational findings reveal a critical design principle: the high stereocontrol stems primarily from spatial shielding and weak non‐covalent interactions rather than specific electronic conjugation of the biaryl skeleton. This suggests that the cumbersome Suzuki–Miyaura coupling—typically required to install such biaryl frameworks—is not strictly necessary, provided an alternative substituent offers equivalent steric confinement.

**SCHEME 4:**
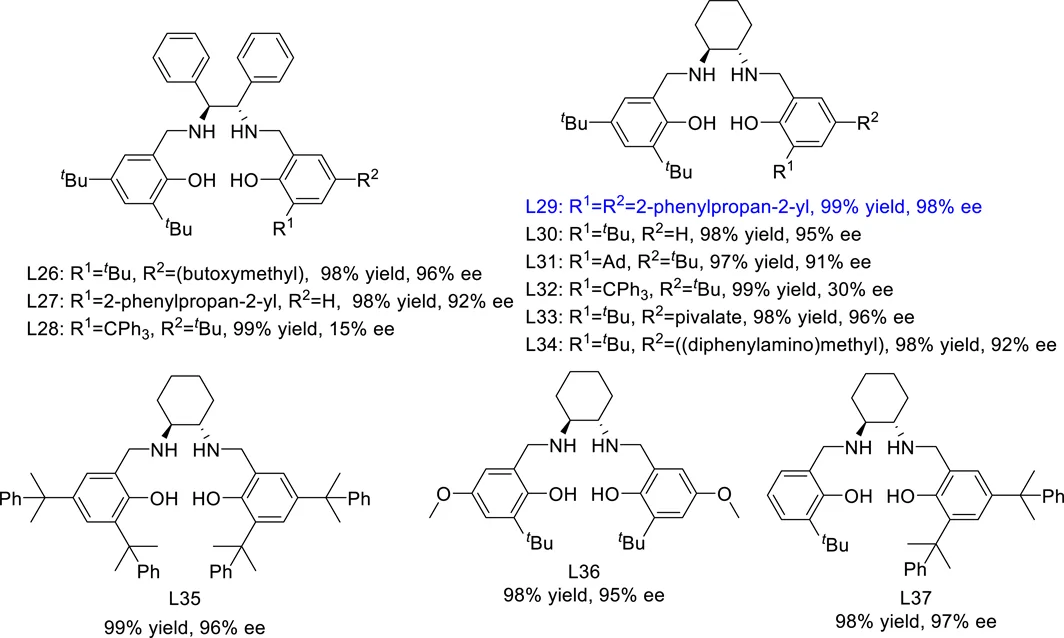
Optimization of salan ligands^*a*, *b*^. ^*a*^Reaction conditions: **1a** (0.1 mmol), CHP (1.5 equiv.), metal salt (5.0 mol%), ligand (5.5 mol%), cumene (2 mL), 50°C, nitrogen. ^*b*^The yields of isolated products. ^*c*^Determined by HPLC on chiral stationary phase.

Guided by DFT calculations, we designed and synthesized a series of inexpensive and readily accessible salan ligands lacking biphenyl motifs as alternatives to costly naphthyl salicylaldehyde units. Meanwhile, the chiral source was replaced with more economical and readily available chiral cyclohexanediamines. As a result, a series of chiral salan ligands were successfully designed and synthesized without the involvement of Suzuki–Miyaura coupling reactions (Scheme 4).

The commercially available 2‐hydroxy‐3,5‐bis(2‐phenylprop‐2‐yl)benzaldehyde was selected as a key building block due to its cost‐effectiveness and the bulky tert‐butyl‐like character of its 3,5‐substituents. Ligand screening revealed that when (S, S)‐1,2‐diphenylethylenediamine was employed as the chiral source, fixing a tert‐butyl group at the 2‐position of the aromatic ring and replacing the 4‐position with a butoxymethyl substituent (**L26**) afforded the hydroxylation product with 96% ee. Further substitution of the 2‐position with a phenylisopropyl group (**L27**) led to a reduced enantioselectivity of 92% ee. Notably, fixing a tert‐butyl group at the 4‐position while introducing a triphenylmethyl group at the 2‐position (**L28** and **L32**) resulted in a dramatic decrease in enantioselectivity to 15% ee and 30%, likely due to excessive steric hindrance that impedes effective substrate coordination to the central zirconium metal.

Upon switching the chiral source to cyclohexanediamine, a series of C1‐symmetric ligands (**L29**–**L34**) was designed by introducing various substituents at the 2‐ and 4‐positions of the aromatic ring. Among them, ligand **L29** exhibited superior performance, delivering the desired product with an excellent enantioselectivity of 98% ee, whereas the remaining ligands afforded enantioselectivities ranging from 30% to 96% ee.

In addition, C2‐symmetric ligands **L35** and **L36** were synthesized, providing the target product with enantioselectivities of 96% ee and 95% ee, respectively. Finally, replacement of the 3,5‐di‐tert‐butyl salicylaldehyde unit in **L29** with a more economical 2‐tert‐butyl salicylaldehyde afforded ligand **L37**, accompanied by a slight decrease in enantioselectivity to 97% ee. Subsequent optimization of reaction conditions using **L29** established the optimal protocol: 2 mol% **L29**–Zr (acac)_4_ in ethyl acetate at 50°C for 180 min (Condition C).

After establishing the optimal reaction condition C, the substrate scope of this catalytic system was systematically evaluated using *β*‐ketoester derivatives of 1‐indanone. For 1‐indanone‐derived *β*‐ketoesters, hydroxylation proceeded smoothly under condition C irrespective of the electronic nature of substituents at C4 (Cl, Br, Me, OMe), C5 (Cl, Br, F, OMe), C6 (Cl, Br, F, OMe), and C7 (OMe) of the aromatic ring, affording the corresponding chiral hydroxy products in excellent yields and enantioselectivities comparable to the **L16**–Zr catalyst (**2a–2c**, **2e–2f**, **2h–2q**; 97%–99% yield, 97%–99% ee). Substrates with electron‐donating methoxy groups at C4 or C5 (**1e**, **1k**) showed a slight reduction in enantioselectivity, yet still delivered 97% ee for **2e** and **2k**. Steric effects of ester substituents (Et, ^*i*^Pr, ^*t*^Bu) were also examined: tert‐butyl esters slightly lowered enantioselectivity (**2ad**, 95% ee), whereas ethyl and isopropyl esters were well tolerated (**2s**, **2aa**; 99% and 98% ee) (Scheme 3).

The comparable enantioselectivity of the simple alkyl‐substituted **L29** (98% ee) versus the naphthyl‐substituted **L16** (99% ee) strongly corroborates our DFT‐based hypothesis: the costly biaryl skeleton is functionally replaceable by the accessible 2‐phenylpropan‐2‐yl group, provided the steric environment is maintained.

For the asymmetric hydroxylation of 1‐tetralone methyl ester, enantioselectivity was strongly temperature‐dependent, likely due to the prevalent enol form of **1al** and associated background reaction. Using **1al** as a model, the reaction was re‐optimized to 5 mol% **L29**–Zr (acac)_4_ in cyclooctene at 15°C for 24 h (Condition D). Under condition D, 1‐tetralone derivatives bearing electron‐withdrawing substituents at C5 (Br), C6 (Br, F), and C7 (Br, F) underwent efficient hydroxylation, affording **2an–2ar** in 81%–88% yield with 81%–89% ee (**2an‐2ar**). Ethyl esters were also compatible (**2am**, 85% yield, 81% ee) (Scheme 3). Mechanistic studies by HRMS detected the key catalytic species [**L29**–Zr (acac)]^+^ (calcd. m/z 861.4148, found 861.4137) and the substrate‐bound intermediate [**L29**–Zr‐**1a**]^+^ (calcd. m/z 985.3864, found 985.3858) (See Supporting Information S1 for details).

**FIGURE 3:**
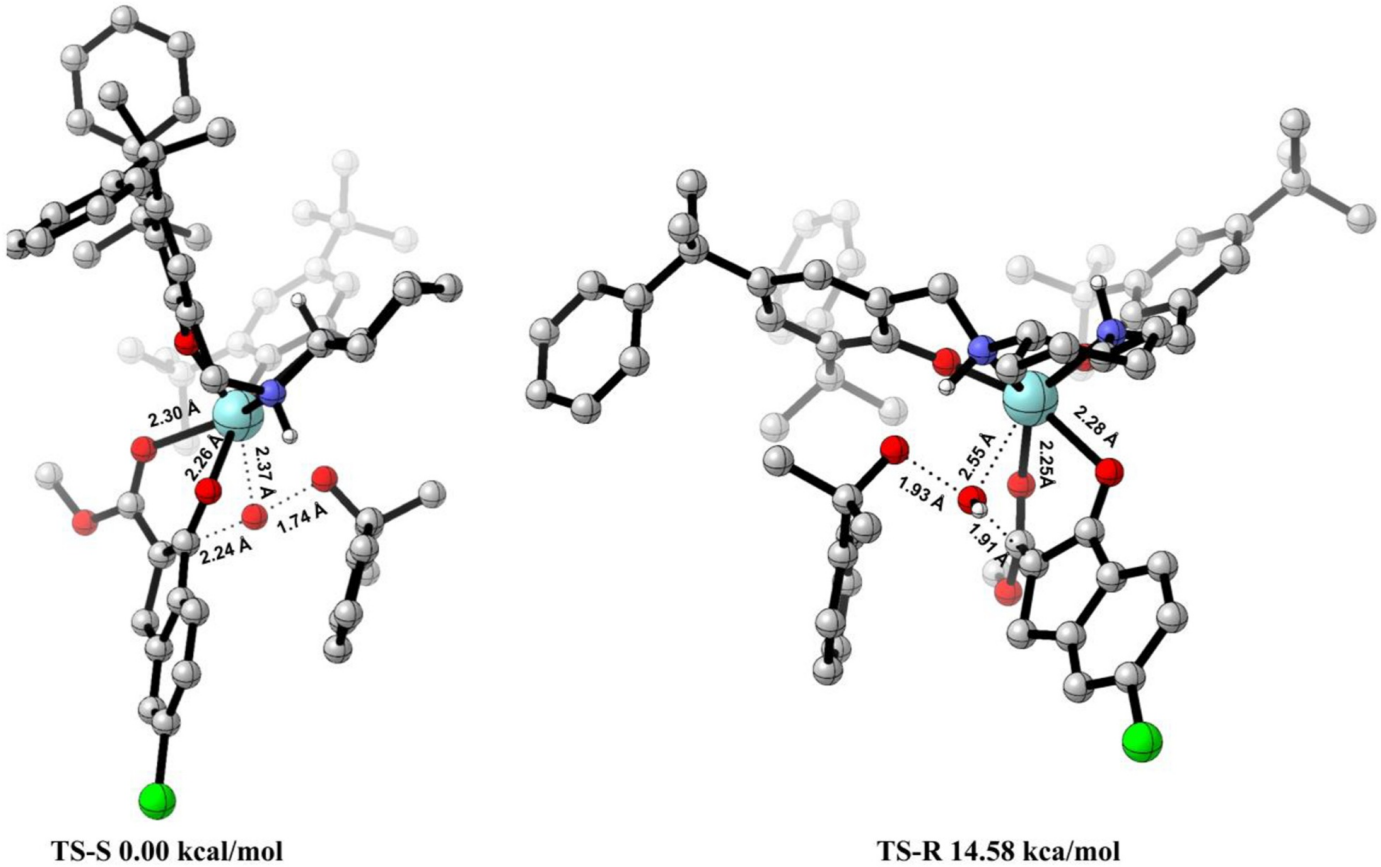
The optimized transition states and the relative Gibbs‐free energies (in kcal/mol) for the most stable one (**L29**). Bond distances are given in Å.

Density functional theory calculations and IGMH analysis of **L29** provided plausible transition‐state structures, showing that the TS leading to the S‐configured product is 14.58 kcal/mol lower in energy than that for the R‐configured product (Figure 3). These results support a stereocontrol model involving weak C–H···C–H interactions between the ligand tert‐butyl group and substrate, C–H···N–H interactions between the ligand and oxidant CHP, and π–π stacking between the substrate and oxidant, consistent with the **L16**–Zr catalytic system (See Supporting Information S1 for details).

In the Zr(IV)‐**L16** catalytic system, where ligand **L16** is functionalized with a 1‐naphthyl group via Suzuki–Miyaura coupling, the energetically favored S‐configured TS (lowest energy barrier) adopts a conformation where the 1‐naphthyl moiety is spatially oriented away from substrate **1a** and CHP. This 1‐naphthyl group acts as a steric shield to construct a well‐defined chiral environment. IGMH analysis of the Zr(IV)‐**L16** system further reveals that the stereocontrol originates mainly from two types of noncovalent interactions: multiple weak C–H···H–C interactions between the tert‐butyl groups of the ligand and the substrate, and π–π stacking between substrate **1a** and CHP. These interactions selectively stabilize the S‐configured TS, thus dictating the enantioselectivity of the reaction.

Analogous structural features are observed in the TS‐S of the reactions of Zr(IV)‐**L29** and Zr(IV)‐**L35** with **1a** and CHP. In their respective favored S‐configured TS‐S, the 1,1‐dimethylphenyl group on the ligands mimics the role of the 1‐naphthyl moiety in **L16**, providing steric shielding that stabilizes the dominant TS, reduces its energy barrier, and delivers enantioselectivity comparable to Zr(IV)‐**L16**. IGMH analysis of the Zr(IV)‐**L35** system with 1a and CHP further validates these findings. These results demonstrate that combined DFT and IGMH analyses are a feasible strategy to guide ligand optimization. By integrating theoretical calculations with chemical intuition, the key structural motifs of chiral ligands that govern stereocontrol can be identified, enabling the rational design of cost‐effective ligands that reproduce the optimal chiral environment.

To verify the generality of this qualitative ligand design strategy, we extended our DFT calculations to additional substrates. TS optimization and IRC calculations were conducted for the reactions of Zr(IV)‐**L16** and Zr(IV)‐**L29** with CHP and substrate 1aa, which bears an isopropyl ester group. Comparison of the energetically favored S‐configured TS‐S shows that the conformations of the 1‐naphthyl group in **L16** and the 1,1‐dimethylphenyl group in **L29** are nearly identical to those observed with substrate **1a**. The 1,1‐dimethylphenyl substituent retains a similar steric shielding effect to the 1‐naphthyl moiety, and both **L16** and **L29** maintain excellent enantioselectivity with this structurally distinct substrate (See Supporting Information S1 for details).

**SCHEME 5:**
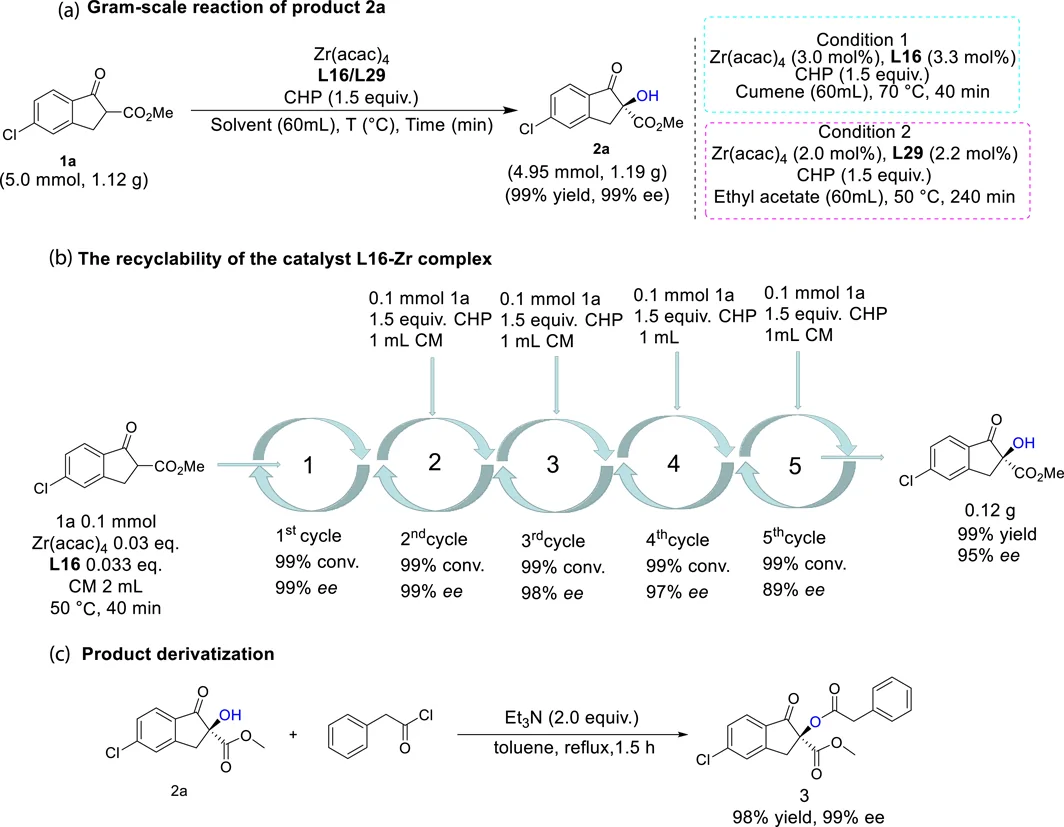
Gram‐scale reaction, recyclability of the catalyst, and product derivation.

To evaluate the practicality of our catalytic system, scale‐up experiments were conducted to assess the practicality of the catalytic system. Using ligands **L16** or **L29**, the desired products were obtained on a gram scale with 99% yield and 99% ee, demonstrating excellent scalability (Scheme 5A). The recyclability of the catalyst was then examined (Scheme 5B). Under 3 mol% **L16**–Zr catalyst, the initial reaction of substrate **1a** was followed by the addition of CHP (1.5 equiv.), cumene (1 mL), and a fresh portion of **1a** to initiate the next cycle. This operation was repeated for successive runs. A gradual erosion in enantioselectivity was observed with increasing cycle number, reaching 89% ee in the fifth cycle, at which point the study was discontinued. The decline in ee is likely attributed to the accumulation of product **2a** and its limited solubility in cumene, leading to precipitation and partial encapsulation of the catalyst, thereby hindering effective coordination of substrate **1a**. Finally, derivatization of product **2a** revealed that its chiral hydroxyl group underwent smooth acylation with 2‐phenylacetyl chloride without loss of enantiomeric purity (Scheme 5C).

**FIGURE 4:**
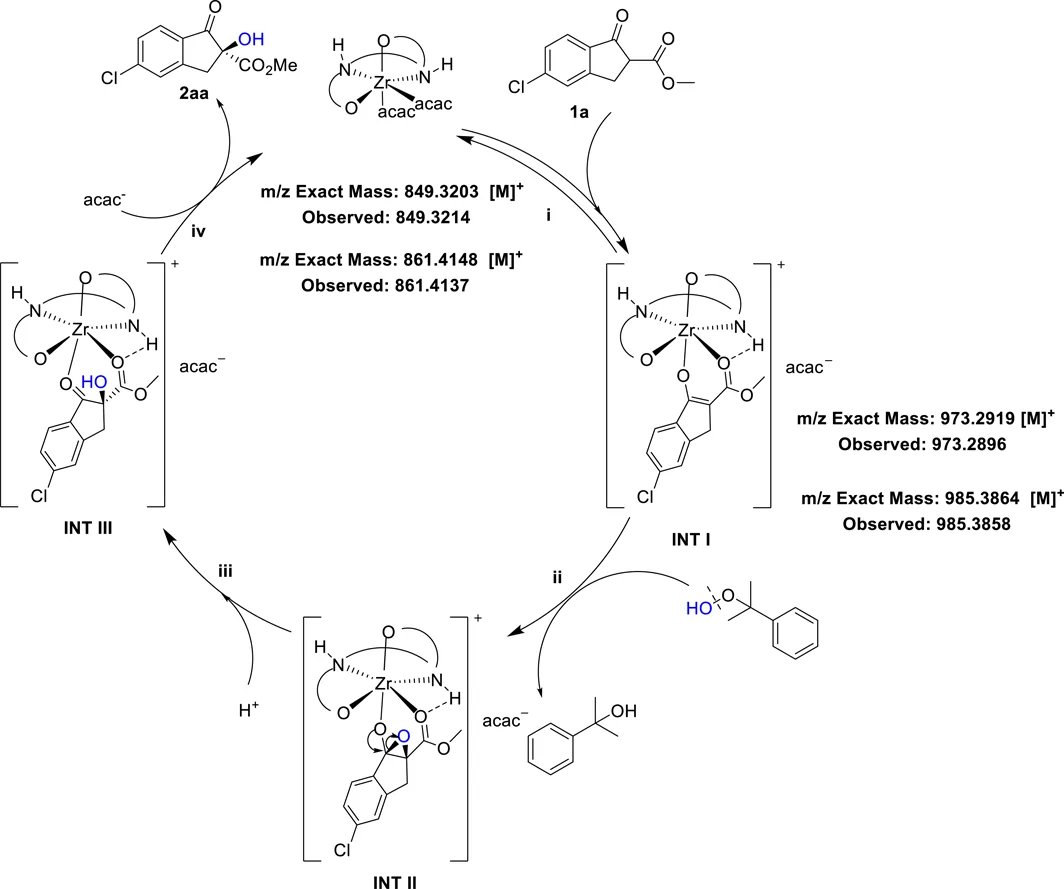
Proposed catalytic cycle.

Based on precedent literature,[^9^, ^10^, ^30^] high‐resolution mass spectrometric data, and DFT‐calculated transition states, a plausible catalytic cycle is proposed (Figure 4). The cycle initiates with the enolization of substrate 1a, followed by coordination to the Zr–**L16/L29** catalyst to form intermediate **INT1**. Subsequently, the O–O Bond cleavage of CHP generates an electrophilic oxidant that attacks the C=C bond of **INT1**, affording the epoxidized intermediate INT2. Proton‐promoted ring‐opening and rearrangement of **INT2**—likely mediated by protons originating from acetylacetone or the substrate—then leads to intermediate **INT3**. Finally, the release of product **2a** regenerates the catalyst and completes the catalytic cycle.

## 3 CONCLUSIONS

In summary, with the assistance of DFT calculations and mechanistic insights, a structurally simple salan ligand was developed. This ligand enabled a highly efficient chiral Zr‐**L16/29** complex catalytic system for the asymmetric *α*‐hydroxylation of 1‐indanone‐ and 1‐tetralone‐derived *β*‐keto esters and amides. Employing a low catalyst loading of 2–3 mol%, this system afforded a broad range of *α*‐hydroxy products in excellent yields with outstanding enantioselectivities (up to 99% yield and 99% ee). The synthetic utility of this methodology was further validated through gram‐scale synthesis, catalyst recycling, and product derivatization. Furthermore, DFT studies provided key insights into the origin of stereocontrol. Current work is focused on extending this catalytic platform to the asymmetric hydroxylation of other substrate classes.

## AUTHOR CONTRIBUTIONS

**Cunfei Ma**: Investigation; methodology; validation; data curation; writing—original draft. **Kun Tang**: Formal analysis; software; methodology; visualization; writing—review and Editing. **Qilei Liu**: Conceptualization; funding acquisition; project administration; supervision; writing—review and editing. **Jian Du**: Conceptualization; funding acquisition; project administration; supervision; writing—review and editing. **Qingwei Meng**: Conceptualization; funding acquisition; project administration; supervision; writing—review and editing.

## CONFLICT OF INTEREST STATEMENT

The authors declare no conflicts of interest.

## ETHICS STATEMENT

No animal or human experiments were involved in this study.

## Supporting information

Supporting Information S1

## Data Availability

The data underlying this study are available in the published article and its Supporting Information.
