# Supplementary material for: DFT‐assisted rational design of salan ligands for highly enantioselective Zr–salan‐catalyzed hydroxylation of cyclic β‐keto esters
Source: Smart Mol. 2026 Aug 27:e70093. Online ahead of print. doi: 10.1002/smo2.70093 (PMC13520753; doi:10.1002/smo2.70093)
Supplement: Supplementary file 1 [file SMO2-9999-0-s001.docx]

**Supporting Information**

**DFT-Assisted Rational Design of Salan Ligands for Highly Enantioselective Zr–Salan-Catalyzed Hydroxylation of cyclic β‑keto esters**

Cunfei Ma^a^, Kun Tang^a^, Yuchen Jiang^a^, Hongfei Zhu^a^, Yufeng Wu^a^, Yafeng Xing^a^, Yakun Wang^b^, Qilei Liu^a^*, Jian Du^a^* and Qingwei Meng^a^*

*State Key Laboratory of Fine Chemicals, Frontiers Science Center for Smart Materials Oriented Chemical Engineering, Dalian University of Technology, Dalian 116024, China.*

*E-mail: Mengqw@dlut.edu.cn.*

[General	2](#_Toc236132837)

[Optimization of the reaction conditions	3](#_Toc236132838)

[Preparation of chiral ligands	10](#_Toc236132839)

[General procedure (A) for the reaction of α-hydroxylation of β-ketoester compounds 1a-1ak	35](#_Toc236132840)

[General procedure (B) for the reaction of α-hydroxylation of β-keto amide compounds 1ah-1ak	36](#_Toc236132841)

[General procedure (C) for the reaction of α-hydroxylation of β-ketoester compounds	36](#_Toc236132842)

[General procedure (D) for the reaction of α-hydroxylation of β-ketoester compounds 1al-1ar	37](#_Toc236132843)

[Mechanism Study	50](#_Toc236132844)

[The gram-scale experiment of substrate 1a.*^a^*	53](#_Toc236132845)

[Transformation of chiral Hydroxylation product	53](#_Toc236132846)

[Computation details	54](#_Toc236132847)

[Optimized transition state structures	55](#_Toc236132848)

[The independent gradient model based on Hirshfeld partition (IGMH)	58](#_Toc236132849)

[References	75](#_Toc236132850)

[NMR spectra	77](#_Toc236132851)

[NMR spectra of chiral ligands	77](#_Toc236132852)

[NMR spectra of hydroxylation products	118](#_Toc236132853)

[HPLC Charts for Hydroxylation Products	165](#_Toc236132854)

# General

Unless otherwise stated, all commercial reagents and solvents were used without additional purification. Analytical TLC was visualized with UV light at 254nm. Thin-layer chromatography was performed on TLC glass sheets coated with silica gel 60 F_254_. Purification of reaction products was carried out by chromatography using silica gel 60 (200-300 mesh). Melting points were determined with a hot plate apparatus and are uncorrected. Optical rotations were measured on a digital polarimeter with a sodium lamp at room temperature (10 cm cell, c given in g/100 mL). Optical rotations were reported as follows: [α] _D_^T^ (c g/100 mL, solvent). All ^1^H NMR (400 MHz) and ^13^C NMR (101 MHz), ^1^H NMR (500 MHz) and ^13^C NMR (126 MHz) were recorded on a VARIAN INOVA-400M and B[ruker AVANCE Ⅲ 500](https://gxpt.dlut.edu.cn/lims/!equipments/equipment/index.1003) spectrometer at 25°C. Chemical shifts are reported in ppm from tetramethylsilane with the solvent resonance as internal standard (CDCl_3_: δ 7.26, for ^1^H NMR and CDCl_3_: δ 77.0 for ^13^C NMR). For ^1^H NMR, data are reported as follows: chemical shift, multiplicity (s = singlet, d = doublet, dd = double doublet, t = triplet, q = quartet, br = broad, m = multiplet), coupling constants (Hz) and integration. High-resolution mass spectrometry data were obtained with UPLC/Q-Tof Mass Spectrometer and were determined by electrospray ionization (ESI).

The enantiomeric excesses (*ee*) were determined by HPLC. Separation of stereoisomers was carried out with a Diacel Chiralpak AD-H, OD-H, and AS-H chiral column (0.46 cm × 25 cm). Retention times were reported at ambient temp (25°C) with an injection volume of 20 µL at a flow rate of 1 mL/min (unless otherwise stated), using mixtures of n-hexane/isopropyl alcohol as mobile phase.

Experimental procedures and data for β-ketoesters have been reported in our previous paper. The racemic products used to determine the ee values were synthesized using 3-chloroperoxybenzoic acid.

Compounds I1–I31 and 1a–1ar were all synthesized in our laboratory^1^. In addition, all reactions described herein that required heating were carried out using an oil bath.

## Optimization of the reaction conditions

Table S1 Screening of metal salts^[a]^

| Entry | Lewis acid | Time(min) | Yield^b^(%) | Ee^c^(%) |
| --- | --- | --- | --- | --- |
| 1 | Mn(OAc)_2_ | 60 | 43 | 12 |
| 2 | Zn(OAc)_2_ | 60 | 25 | -3 |
| 3 | Co(OAc)_2_ | 60 | 18 | -18 |
| 4 | Cu(acac)_2_ | 60 | 53 | 11 |
| 5 | NiBr_2_ | 60 | 9 | 7 |
| 6 | Zn(OTf)_2_ | 60 | 3 | 0 |
| 7 | Mn(acac)_2_ | 60 | 68 | 9 |
| 8 | Zr(acac)_4_ | 60 | 98 | 97 |
| 9 | Fe(acac)_3_ | 60 | 4 | 13 |
| 10 | Ni(acac)_2_ | 60 | 76 | 15 |
| 11 | Ni(OTf)_2_ | 60 | 2 | 17 |

a) Unless otherwise specified, the reactions are conducted with **1a** (0.1 mmol), Lewis acid (5 mol%)/**L1** (5.5 mol%), and 1.5 equiv. CHP in 2 mL Cumene under 50 ^o^C for 60 min. b) Isolated yields. c) Determined by HPLC on a chiral stationary phase.

Table S2 Screening of Solvents^[a]^

| Entry | Solvent | Time(min) | Yield^b^(%) | Ee^c^(%) |
| --- | --- | --- | --- | --- |
| 1 | toluene | 60 | 98 | 97 |
| 2 | *p*-Xylene | 60 | 98 | 98 |
| 3 | *o*-Xylene | 60 | 98 | 97 |
| 4 | *m*-Xylene | 60 | 99 | 97 |
| 5 | mesitylene | 60 | 99 | 98 |
| 6 | n-hexane | 60 | 98 | 95 |
| 7 | cyclohexane | 60 | 98 | 95 |
| 8 | cumene | 60 | 98 | 98 |
| 9 | methyl tert-butyl ether | 180 | 89 | 91 |
| 10 | acetonitrile | 180 | 98 | 88 |
| 11 | acetone | 180 | 88 | 92 |
| 12 | chloroform | 180 | 98 | 95 |
| 13 | 1,4-dioxane | 180 | 48 | 87 |
| 14 | 1,2-Dimethoxyethane | 180 | 60 | 90 |

a) Unless otherwise specified, the reactions are conducted with **1a** (0.1 mmol), Zr(acac)_4_ (5 mol%)/**L1** (5.5 mol%), and 1.5 equiv. CHP in 2 mL Cumene under 50 ^o^C for 1 h. b) Isolated yields. c) Determined by HPLC on a chiral stationary phase.

Table S3 Ligands evaluated in the hydroxylation reaction^[a]^

| Entry | Ligand | Time(min) | Yield^b^(%) | Ee^c^(%) |
| --- | --- | --- | --- | --- |
| 1 | L1 | 60 | 98 | 97 |
| 2 | L2 | 60 | 99 | 98 |
| 3 | L3 | 60 | 84 | 90 |
| 4 | L4 | 60 | 98 | 96 |
| 5 | L5 | 60 | 98 | 73 |
| 6 | L6 | 60 | 98 | 97 |
| 7 | L7 | 60 | 98 | 94 |
| 8 | L8 | 60 | 98 | 97 |
| 9 | L9 | 60 | 98 | 91 |
| 10 | L10 | 60 | 97 | 94 |
| 11 | L11 | 60 | 99 | 98 |
| 12 | L12 | 60 | 99 | 96 |
| 13 | L13 | 60 | 99 | 99 |
| 14 | L14 | 60 | 99 | 98 |
| 15 | L15 | 60 | 98 | 97 |
| 16 | L16 | 60 | 99 | 99 |
| 17 | L17 | 60 | 99 | 99 |
| 18 | L18 | 60 | 99 | 99 |
| 19 | L19 | 60 | 99 | 99 |
| 20 | L20 | 60 | 99 | 99 |
| 21 | L21 | 60 | 99 | 99 |
| 22 | L22 | 60 | 99 | 99 |
| 23 | L23 | 60 | 99 | 98 |
| 24 | L24 | 60 | 99 | 99 |
| 25 | L25 | 60 | 93 | 97 |

a) Unless otherwise specified, the reactions are conducted with **1a** (0.1 mmol), Zr(acac)_4_ (5 mol%)/Ligand (5.5mol %), and 1.5 equiv. CHP in 2 mL Cumene under 50 ^o^C for 1 h. b) Isolated yields. c) Determined by HPLC on a chiral stationary phase.

Table S4 Ligands loading evaluated in the Asymmetric hydroxylation reaction^[a]^

| Entry | Ligand | Catalyst loading | Yield^b^(%) | Ee^c^(%) |
| --- | --- | --- | --- | --- |
| 1 | L13 | 3.0 mol% | 99 | 98 |
| 2 | L16 | 3.0 mol% | 99 | 99 |
| 3 | L16 | 2.0 mol% | 99 | 98 |
| 4 | L17 | 3.0 mol% | 99 | 98 |
| 5 | L18 | 3.0 mol% | 99 | 97 |
| 6 | L20 | 3.0 mol% | 99 | 98 |
| 7 | L21 | 3.0 mol% | 99 | 99 |
| 8 | L21 | 2.0 mol% | 99 | 98 |
| 9 | L22 | 3.0 mol% | 99 | 98 |
| 10 | L24 | 3.0 mol% | 99 | 97 |

a) Unless otherwise specified, the reactions are conducted with **1a** (0.1 mmol), Zr(acac)_4_ (x mol%)/Ligand (y mol%), and 1.5 equiv. CHP in 2 mL Cumene under 50 ^o^C for 1 h. b) Isolated yields. c) Determined by HPLC on a chiral stationary phase.

Table S5 Screening of Ligand/metal salts ratio^[a]^

| Entry | Ligand/Metal salts | Time(min) | Yield^b^(%) | Ee^c^(%) |
| --- | --- | --- | --- | --- |
| 1 | 0.9:1.0 | 60 | 96 | 93 |
| 2 | 1.0:1.0 | 60 | 97 | 97 |
| 3 | 1.1:1.0 | 60 | 99 | 99 |
| 4 | 1.2:1.0 | 60 | 99 | 99 |

a) Unless otherwise specified, the reactions are conducted with **1a** (0.1 mmol), Zr(acac)_4_ (3 mol%)/**L16** (3.3x mol%), and 1.5 equiv. CHP in 2 mL Cumene under 50 ^o^C for 1 h. b) Isolated yields. c) Determined by HPLC on a chiral stationary phase.

Table S6 Screening of Oxidan^[a]^

| Entry | Oxidant | Time(min) | Yield^b^(%) | Ee^c^(%) |
| --- | --- | --- | --- | --- |
| 1 | TBHP | 60 | 97 | 97 |
| 2 | H_2_O_2_ | 60 | 17 | -9 |
| 3 | *m*-CPBA | 60 | 93 | 0 |
| 4 | CHP | 60 | 99 | 99 |

a) Unless otherwise specified, the reactions are conducted with **1a** (0.1 mmol), Zr(acac)_4_ (3 mol%)/**L16** (3.3 mol%), and 1.5 equiv. Oxidant in 2 mL Cumene under 50 ^o^C for 1 h. b) Isolated yields. c) Determined by HPLC on a chiral stationary phase.

Table S7 Screening of temperature^[a]^

| Entry | Temperature(℃) | Time(min) | Yield^b^(%) | Ee^c^(%) |
| --- | --- | --- | --- | --- |
| 1 | 30 | 180 | 99 | 99 |
| 2 | 40 | 110 | 99 | 99 |
| 3 | 50 | 60 | 99 | 99 |
| 4 | 60 | 45 | 99 | 99 |
| 5 | 70 | 20 | 99 | 99 |
| 6 | 80 | 10 | 98 | 98 |

a) Unless otherwise specified, the reactions are conducted with **1a** (0.1 mmol), Zr(acac)_4_ (3.0 mol%)/**L16** (3.3 mol%), and 1.5 equiv. CHP in 2 mL Cumene under T ^o^C. b) Isolated yields. c) Determined by HPLC on a chiral stationary phase.

Table S8 Screening of concentration^[a]^

| Entry | Cumene(x ml) | Time(min) | Yield^b^(%) | Ee^c^(%) |
| --- | --- | --- | --- | --- |
| 1 | 0.5 | 60 | 99 | 96 |
| 2 | 1 | 60 | 99 | 97 |
| 3 | 2 | 60 | 99 | 99 |
| 4 | 3 | 60 | 99 | 99 |

a) Unless otherwise specified, the reactions are conducted with **1a** (0.1 mmol), Lewis acid (3.0 mol%)/**L16** (3.3 mol%), and 1.5 equiv. CHP in 2 mL Cumene under 50 ^o^C for 60 min. b) Isolated yields. c) Determined by HPLC on a chiral stationary phase.

Table S9 Ligands evaluated in the hydroxylation reaction^[a]^

| Entry | Ligand | Time(min) | Yield^b^(%) | Ee^c^(%) |
| --- | --- | --- | --- | --- |
| 1 | L26 | 60 | 98 | 96 |
| 2 | L27 | 60 | 98 | 92 |
| 3 | L28 | 60 | 99 | 15 |
| 4 | L29 | 60 | 99 | 98 |
| 5 | L30 | 60 | 98 | 95 |
| 6 | L31 | 60 | 97 | 91 |
| 7 | L32 | 60 | 99 | 30 |
| 8 | L33 | 60 | 98 | 96 |
| 9 | L34 | 60 | 98 | 92 |
| 10 | L35 | 60 | 99 | 96 |
| 11 | L36 | 60 | 98 | 95 |
| 12 | L37 | 60 | 98 | 97 |

b) Unless otherwise specified, the reactions are conducted with **1a** (0.1 mmol), Zr(acac)_4_ (5 mol%)/Ligand (5.5 mol%), and 1.5 equiv. CHP in 2 mL Cumene under 50 ^o^C for 1 h. b) Isolated yields. c) Determined by HPLC on a chiral stationary phase.

Table S10 Screening of Solvents with L29^[a]^

| Entry | Solvent | Time(min) | Yield^b^(%) | Ee^c^(%) |
| --- | --- | --- | --- | --- |
| 1 | toluene | 60 | 98 | 87 |
| 2 | *p*-Xylene | 60 | 98 | 96 |
| 3 | *o*-Xylene | 60 | 98 | 88 |
| 4 | *m*-Xylene | 60 | 99 | 93 |
| 5 | mesitylene | 60 | 99 | 90 |
| 6 | n-hexane | 60 | 98 | 97 |
| 7 | cyclohexane | 60 | 98 | 96 |
| 8 | cumene | 60 | 98 | 98 |
| 9 | methyl tert-butyl ether | 180 | 89 | 98 |
| 10 | acetonitrile | 180 | 97 | 91 |
| 11 | acetone | 180 | 89 | 92 |
| 12 | chloroform | 180 | 95 | 97 |
| 13 | ethyl acetate | 180 | 99 | 99 |
| 14 | methanol | 180 | 94 | 90 |

b) Unless otherwise specified, the reactions are conducted with **1a** (0.1 mmol), Zr(acac)_4_ (5 mol%)/**L29** (5.5 mol%), and 1.5 equiv. CHP in 2 mL Cumene under 50 ^o^C . b) Isolated yields. c) Determined by HPLC on a chiral stationary phase.

Table S11 Ligands loading evaluated in the Asymmetric hydroxylation reaction^[a]^

| Entry | Ligand | Catalyst loading | Yield^b^(%) | Ee^c^(%) |
| --- | --- | --- | --- | --- |
| 1 | L29 | 3.0 mol% | 99 | 99 |
| 2 | L29 | 2.0 mol% | 99 | 99 |
| 3 | L29 | 1.0 mol% | 99 | 97 |

a) Unless otherwise specified, the reactions are conducted with **1a** (0.1 mmol), Zr(acac)_4_ (x mol%)/**L29** (1.1x mol%), and 1.5 equiv. CHP in 2 mL ethyl acetate under 50 ^o^C for 3 h. b) Isolated yields. c) Determined by HPLC on a chiral stationary phase.

Table S12 Screening of temperature^[a]^

| Entry | Temperature(℃) | Time(min) | Yield^b^(%) | Ee^c^(%) |
| --- | --- | --- | --- | --- |
| 1 | 30 | 480 | 99 | 99 |
| 2 | 40 | 300 | 99 | 99 |
| 3 | 50 | 180 | 99 | 99 |
| 4 | 60 | 140 | 99 | 98 |
| 5 | 70 | 110 | 99 | 97 |
| 6 | 80 | 70 | 98 | 96 |

b) Unless otherwise specified, the reactions are conducted with **1a** (0.1 mmol), Zr(acac)_4_ (2.0 mol%)/**L29** (2.2 mol%), and 1.5 equiv. CHP in 2 mL ethyl acetate under T ^o^C. b) Isolated yields. c) Determined by HPLC on a chiral stationary phase.

## Preparation of chiral ligands

**Synthesis of ligands L1-29^2^**

Synthesis of (1S,2S)-2-amino-1,2-diphenylethan-1-aminium chloride (M2)^2a^

(1S,2S)-1,2-diphenylethane-1,2-diamine mono(hydrogen chloride) was prepared according to a published procedure of (±)-1,2-cyclohexanediamine mono(hydrogen chloride) : (1S,2S)-1,2-diphenylethane-1,2-diamine (10.0 g, 47.1 mmol) was dissolved in anhydrous ethanol (100 mL) under argon in a 500 mL three-necked flask equipped with a magnetic stir bar, an addition funnel, and a gas inlet. After the solution was cooled to 0 °C with an ice/water bath, a solution of hydrogen chloride in ether (10.0 M, 47.1 mmol) was added dropwise through the addition funnel over a period of 60 minutes. The reaction was exothermic and a white precipitate formed immediately. The reaction mixture was allowed to warm up to room temperature and stirring was continued overnight. The precipitate was collected by filtration, washed with dry Et_2_O (ca. 100 mL), and dried in vacuo to afford M2 (10.78 g, 92 %) as a colorless powder.

To a solution of M2 (5.83 g, 23.44 mmol, 1.0 equiv.) in EtOH (100 mL) was added 3,5-di-tert-butyl-2-hydroxybenzaldehyde (5.49 g, 23.44 mmol, 1.0 equiv.) in one portion at 20 °C. The mixture was stirred for 4 hours at 20 °C, then cooled to 0 °C with an ice bath, and sodium borohydride (1.78 g, 47.13 mmol, 2.01 equiv.) was added portionwise over 1 hour. The ice bath was removed, and the suspension was stirred at 20 °C for 6 hours. The reaction was quenched with saturated sodium bicarbonate (150 mL) and the resulting mixture was extracted with Et_2_O (3 x 100 mL). The combined organic layers were washed with H_2_O (3 x 50 mL) and brine (50 mL), dried over sodium sulfate, filtered and the solvent removed under reduced pressure to yield M3 that were used in the next step without further purification.

Synthesis of Ligand intermediates I1-I31

Synthesis of Ligands L1-L25^2c, 3^

A tetrahydrofuran solution of intermediate I (2.5 mmol, 1.0 equiv.) was added to anhydrous ethanol (50 mL) solution of M3 (2.5 mmol, 1.0 equiv.) under nitrogen protection. The reaction was carried out at reflux, and the reaction progress was monitored by TLC at the end of the reaction. The temperature was lowered to room temperature, and the solvent was removed under reduced pressure. Anhydrous methanol was added, the temperature was lowered to 0 °C, after nitrogen purging, NaBH_4_ (5.0 mmol, 2.0 equiv.) was added in batches, stirring was continued at 0 °C for 30 min, then transferred to room temperature, the reaction progress was monitored by TLC, and at the end of the reaction, the reaction was quenched by adding deionized water. Dichloromethane was extracted three times (3×100 ml), all organic phases were collected, the Organic layer was washed with brine, and finally dried over anhydrous Na_2_SO_4_. Dichloromethane was removed under reduced pressure, followed by purification by silica gel chromatography (PE: EA=5:1).

2-**((((1S,2S)-2-amino-1,2-diphenylethyl)amino)methyl)-4,6-bis(2-phenylpropan-2-yl)phenol**

**(M3)**

The general procedure was followed using M2 (5.83 g, 23.44 mmol), 3,5-di-tert-butyl-2-hydroxybenzaldehyde (5.49 g, 23.44 mmol) (10.96 g, 40 mmol) and sodium borohydride (1.78 g, 47.13 mmol). The product **M3** was obtained as a white solid (9.08 g, 21.1 mmol, 90% yield). [α]_D_^25^ = -21 (c 0.10, CHCl_3_); m.p. 163-165 °C; ^1^H NMR (400 MHz, Chloroform-d) δ 7.29 – 7.19 (m, 9H), 7.14 (tq, J = 6.6, 3.8 Hz, 8H), 6.95 (dd, J = 6.6, 3.0 Hz, 2H), 6.90 (dd, J = 6.6, 2.9 Hz, 2H), 6.50 (d, J = 2.3 Hz, 1H), 3.96 (d, J = 7.0 Hz, 1H), 3.66 (d, J = 13.4 Hz, 1H), 3.57 (d, J = 7.0 Hz, 1H), 3.40 (d, J = 13.4 Hz, 1H), 1.67 (s, 3H), 1.64 (s, 3H), 1.62 (d, J = 4.3 Hz, 6H).

^13^C{^1^H} NMR (101 MHz, CDCl_3_) δ 154.4, 143.0, 140.4, 139.5, 135.9, 128.5, 128.3, 127.9, 127.5, 127.3, 127.0, 123.4, 122.9, 122.6, 69.0, 61.2, 51.4, 35.0, 34.1, 31.7, 29.7.

HRMS (ESI) m/z: [M + H]^+^ Calcd for C_29_H_39_ON_2_ 431.3057, Found: 431.3059.

**2-((((1S,2S)-2-aminocyclohexyl)amino)methyl)-4,6-di-tert-butylphenol (M4)**

**M4** is synthesized based on the preparation process of **M3**. The product **M4** was obtained as a white solid (8.0 g, 24.1 mmol, 90% yield). [α]_D_^25^ = +43 (c 0.23, CHCl_3_); m.p. 89-93 °C; ^1^H NMR (400 MHz, CDCl_3_) δ 7.21 (d, *J* = 2.4 Hz, 1H), 6.87 (d, *J* = 2.4 Hz, 1H), 4.08 (d, *J* = 13.3 Hz, 1H), 3.82 (d, *J* = 13.3 Hz, 1H), 2.44 (ddd, *J* = 10.7, 9.1, 4.1 Hz, 1H), 2.17 (td, *J* = 10.8, 4.0 Hz, 2H), 1.90 (ddt, *J* = 12.6, 5.0, 2.5 Hz, 1H), 1.77 – 1.63 (m, 2H), 1.42 (s, 9H), 1.28 (s, 9H), 1.25 – 1.09 (m, 4H).

^13^C{^1^H} NMR (101 MHz, CDCl_3_) δ 154.6, 140.4, 135.9, 123.1, 123.0, 122.8, 63.6, 54.9, 50.8, 36.6, 34.9, 34.2, 31.8, 30.9, 29.7, 25.2, 24.9.

HRMS (ESI) m/z: [M + H]^+^ Calcd for C_21_H_37_ON_2_ 333.2900, Found: 333.2910.

**2-((((1S,2S)-2-aminocyclohexyl)amino)methyl)-6-(tert-butyl)phenol (M5)**

**M5** is synthesized based on the preparation process of **M5**. The product **M5** was obtained as a white solid (6.5 g, 23.5 mmol, 87% yield). [α]_D_^25^ = +37 (c 0.20, CHCl_3_); m.p. 79-83 °C; ^1^H NMR (500 MHz, CDCl_3_) δ 7.17 (d, *J* = 7.7 Hz, 1H), 6.86 (d, *J* = 7.3 Hz, 1H), 6.70 (t, *J* = 7.6 Hz, 1H), 4.06 (d, *J* = 13.5 Hz, 1H), 3.86 (d, *J* = 13.4 Hz, 1H), 2.44 (td, *J* = 10.9, 3.7 Hz, 1H), 2.14 (q, *J* = 6.1 Hz, 2H), 1.90 (d, *J* = 12.4 Hz, 1H), 1.70 (t, *J* = 13.5 Hz, 2H), 1.41 (d, *J* = 2.2 Hz, 9H), 1.26 – 1.03 (m, 4H).

^13^C{^1^H} NMR (126 MHz, CDCl_3_) δ 157.2, 136.8, 126.3, 125.8, 123.9, 118.2, 63.6, 54.8, 50.5, 36.6, 34.7, 30.8, 29.6, 25.2, 24.9.

HRMS (ESI) m/z: [M + H]^+^ Calcd for C_17_H_29_ON_2_ 277.2274, Found: 277.2280.

**2,4-di-tert-butyl-6-((((1S,2S)-2-((3-(tert-butyl)-2-hydroxybenzyl)amino)-1,2-diphenylethyl)amino)methyl)phenol (L1)**

A tetrahydrofuran solution of intermediate **I1** was added to an anhydrous ethanol solution of **M3** under a nitrogen atmosphere. The reaction mixture was heated under reflux, with progress monitored by TLC. Upon completion, the mixture was cooled to room temperature and concentrated under reduced pressure. The residue was dissolved in anhydrous methanol and cooled to 0 °C. After purging with nitrogen, NaBH₄ was added portionwise, and the mixture was stirred at 0 °C for 30 min. The reaction was warmed to room temperature (monitored by TLC). After completion, the reaction was quenched with deionized water and extracted with dichloromethane (3 × 100 mL). The combined organic layers were washed with brine, dried over anhydrous sodium sulfate (Na₂SO₄), filtered, and concentrated. The crude product was purified by silica gel chromatography (PE/EA = 5:1) to afford **L1.** (1.42 g, 2.40 mmol, 96% yield); [α]_D_^25^ = -16 (c 0.10, CHCl_3_); m.p. 78-80 °C; ^1^H NMR (400 MHz, CDCl_3_) δ 7.23 (ddt, *J* = 6.8, 4.4, 2.2 Hz, 7H), 7.18 (dd, *J* = 7.0, 2.5 Hz, 1H), 6.94 (dd, *J* = 6.6, 2.9 Hz, 4H), 6.70 – 6.61 (m, 3H), 4.02 (s, 2H), 3.82 (dd, *J* = 13.4, 11.5 Hz, 2H), 3.61 (d, *J* = 13.4 Hz, 2H), 1.43 (d, *J* = 3.7 Hz, 18H), 1.22 (s, 9H).

^13^C{^1^H} NMR (101 MHz, CDCl_3_) δ 156.9, 154.3, 140.7, 137.7, 137.6, 137.0, 136.1, 128.5, 128.46, 128.2, 128.15, 128.0, 126.8, 126.2, 123.5, 123.2, 122.6, 121.7, 118.5, 66.5, 66.3, 51.2, 50.7, 35.0, 34.8, 34.1, 31.6, 29.7, 29.6.

HRMS (ESI) *m/z*: [M + H]^+^ Calcd for C_40_H_53_O_2_N_2_ 593.4102, Found: 593.4110.

**2,4-di-tert-butyl-6-((((1S,2S)-2-((3-(tert-butyl)-2-hydroxy-5-methoxybenzyl)amino)-1,2-diphenylethyl)amino)methyl)phenol (L2)**

A tetrahydrofuran solution of intermediate **I2** was added to an anhydrous ethanol solution of **M3** under a nitrogen atmosphere. The reaction mixture was heated under reflux, with progress monitored by TLC. Upon completion, the mixture was cooled to room temperature and concentrated under reduced pressure. The residue was dissolved in anhydrous methanol and cooled to 0 °C. After purging with nitrogen, NaBH₄ was added portionwise, and the mixture was stirred at 0 °C for 30 min. The reaction was warmed to room temperature (monitored by TLC). After completion, the reaction was quenched with deionized water and extracted with dichloromethane (3 × 100 mL). The combined organic layers were washed with brine, dried over anhydrous sodium sulfate (Na₂SO₄), filtered, and concentrated. The crude product was purified by silica gel chromatography (PE/EA = 5:1) to afford **L2** (1.46 g, 2.35 mmol, 94% yield). [α]_D_^25^ = -19 (c 0.10, CHCl_3_); m.p. 80-82 °C; ^1^H NMR (400 MHz, CDCl_3_) δ 7.22 (dq, *J* = 6.0, 2.0 Hz, 7H), 7.01 – 6.87 (m, 4H), 6.79 (d, *J* = 3.0 Hz, 1H), 6.63 (d, *J* = 2.4 Hz, 1H), 6.20 (d, *J* = 3.0 Hz, 1H), 4.00 (s, 2H), 3.81 (dd, *J* = 13.3, 3.7 Hz, 2H), 3.66 (s, 3H), 3.63 – 3.52 (m, 2H), 1.42 (d, *J* = 7.9 Hz, 18H), 1.22 (s, 9H).

^13^C{^1^H} NMR (101 MHz, CDCl_3_) δ 154.3, 151.8, 150.6, 140.7, 138.3, 137.8, 137.8, 136.1, 128.5, 128.45, 128.2, 127.9, 123.5, 123.2, 122.9, 121.7, 113.0, 111.2, 66.6, 66.4, 55.7, 51.2, 50.9, 35.0, 34.9, 34.1, 31.7, 29.7, 29.5.

HRMS (ESI) *m/z*: [M + H]^+^ Calcd for C_41_H_55_O_3_N_2_ 623.4207, Found: 623.4211.

**2-((((1S,2S)-2-((3-bromo-2-hydroxybenzyl)amino)-1,2-diphenylethyl)amino)methyl)-4,6-di-tert-butylphenol (L3)**

A tetrahydrofuran solution of intermediate **I3** was added to an anhydrous ethanol solution of **M3** under a nitrogen atmosphere. The reaction mixture was heated under reflux, with progress monitored by TLC. Upon completion, the mixture was cooled to room temperature and concentrated under reduced pressure. The residue was dissolved in anhydrous methanol and cooled to 0 °C. After purging with nitrogen, NaBH₄ was added portionwise, and the mixture was stirred at 0 °C for 30 min. The reaction was warmed to room temperature (monitored by TLC). After completion, the reaction was quenched with deionized water and extracted with dichloromethane (3 × 100 mL). The combined organic layers were washed with brine, dried over anhydrous sodium sulfate (Na₂SO₄), filtered, and concentrated. The crude product was purified by silica gel chromatography (PE/EA = 5:1) to afford **L3.** (1.43 g, 2.33 mmol, 93% yield); [α]_D_^25^ = 4.0 (c 0.10, CHCl_3_); m.p. 81-83 °C; ^1^H NMR (400 MHz, CDCl_3_) δ 7.39 (dd, *J* = 8.0, 1.6 Hz, 1H), 7.25 (dd, *J* = 5.1, 1.9 Hz, 3H), 7.22 (dt, *J* = 4.6, 2.0 Hz, 4H), 6.94 (ddd, *J* = 8.0, 4.6, 1.7 Hz, 4H), 6.76 – 6.70 (m, 1H), 6.66 – 6.57 (m, 2H), 4.01 (s, 2H), 3.83 (dd, *J* = 16.4, 13.6 Hz, 2H), 3.59 (d, *J* = 13.6 Hz, 2H), 1.42 (s, 9H), 1.23 (s, 9H).

^13^C{^1^H} NMR (101 MHz, CDCl_3_) δ 154.5, 154.1, 140.8, 137.3, 137.0, 136.1, 132.3, 128.6, 128.5, 128.3, 128.1, 128.09, 127.6, 123.7, 123.6, 123.3, 121.5, 120.1, 110.5, 66.6, 66.0, 50.9, 50.1, 35.0, 34.2, 31.7, 29.7.

HRMS (ESI) *m/z*: [M + H]^+^ Calcd for C_36_H_44_O_2_N_2_Br 615.2581, Found: 615.2583.

**2,4-di-tert-butyl-6-((((1S,2S)-2-((3-(tert-butyl)-5-((dimethylamino)methyl)-2-hydroxybenzyl)amino)-1,2-diphenylethyl)amino)methyl)phenol (L4)**

A tetrahydrofuran solution of intermediate **I4** was added to an anhydrous ethanol solution of **M3** under a nitrogen atmosphere. The reaction mixture was heated under reflux, with progress monitored by TLC. Upon completion, the mixture was cooled to room temperature and concentrated under reduced pressure. The residue was dissolved in anhydrous methanol and cooled to 0 °C. After purging with nitrogen, NaBH₄ was added portionwise, and the mixture was stirred at 0 °C for 30 min. The reaction was warmed to room temperature (monitored by TLC). After completion, the reaction was quenched with deionized water and extracted with dichloromethane (3 × 100 mL). The combined organic layers were washed with brine, dried over anhydrous sodium sulfate (Na₂SO₄), filtered, and concentrated. The crude product was purified by silica gel chromatography (PE/EA = 5:1) to afford **L4.** (1.54 g, 2.37 mmol, 95% yield); [α]_D_^25^ = -23.0 (c 0.10, CHCl_3_); m.p. 97-99 °C; ^1^H NMR (400 MHz, CDCl_3_) δ 10.66 – 9.71 (m, 2H), 7.15 (ddd, *J* = 7.7, 4.8, 1.8 Hz, 7H), 6.97 (d, *J* = 2.1 Hz, 1H), 6.86 (dq, *J* = 5.1, 2.5 Hz, 4H), 6.55 (d, *J* = 2.4 Hz, 1H), 6.52 (d, *J* = 2.0 Hz, 1H), 3.93 (d, *J* = 3.7 Hz, 2H), 3.72 (d, *J* = 13.2 Hz, 2H), 3.53 (d, *J* = 13.4 Hz, 2H), 3.23 – 3.07 (m, 2H), 2.09 (s, 6H), 1.35 (d, *J* = 2.7 Hz, 18H), 1.15 (s, 9H).

^13^C{^1^H} NMR (101 MHz, CDCl_3_) δ 155.8, 154.3, 140.7, 137.7, 137.6, 136.5, 136.1, 128.5, 128.4, 128.2, 127.9, 127.4, 127.2, 123.5, 123.2, 122.4, 121.7, 66.6, 66.56, 64.3, 51.2, 50.80, 45.3, 34.98, 34.7, 34.1, 31.7, 29.7, 29.66.

HRMS (ESI) *m/z*: [M + 2H]^2+^/2 Calcd for C_21.5_H_30.5_ON_1.5_ 325.7376, Found: 325.7380.

**2,4-di-tert-butyl-6-((((1S,2S)-2-((3-(tert-butyl)-5-((diethylamino)methyl)-2-hydroxybenzyl)amino)-1,2-diphenylethyl)amino)methyl)phenol (L5)**

A tetrahydrofuran solution of intermediate **I5** was added to an anhydrous ethanol solution of **M3** under a nitrogen atmosphere. The reaction mixture was heated under reflux, with progress monitored by TLC. Upon completion, the mixture was cooled to room temperature and concentrated under reduced pressure. The residue was dissolved in anhydrous methanol and cooled to 0 °C. After purging with nitrogen, NaBH₄ was added portionwise, and the mixture was stirred at 0 °C for 30 min. The reaction was warmed to room temperature (monitored by TLC). After completion, the reaction was quenched with deionized water and extracted with dichloromethane (3 × 100 mL). The combined organic layers were washed with brine, dried over anhydrous sodium sulfate (Na₂SO₄), filtered, and concentrated. The crude product was purified by silica gel chromatography (PE/EA = 5:1) to afford **L5.** (1.47 g, 2.16 mmol, 87% yield); [α]_D_^25^ = -25 (c 0.10, CHCl_3_); m.p. 70-72 °C; ^1^H NMR (400 MHz, CDCl_3_) δ 10.63 – 9.99 (m, 2H), 7.16 (t, *J* = 3.4 Hz, 4H), 7.14 (d, *J* = 2.1 Hz, 3H), 7.00 (d, *J* = 2.0 Hz, 1H), 6.91 – 6.82 (m, 4H), 6.55 (d, *J* = 2.3 Hz, 2H), 3.94 (s, 2H), 3.72 (d, *J* = 13.3 Hz, 2H), 3.53 (d, *J* = 13.3 Hz, 2H), 3.33 (s, 2H), 2.42 – 2.36 (m, 4H), 1.35 (d, *J* = 2.8 Hz, 18H), 1.15 (d, *J* = 1.6 Hz, 9H), 0.92 (t, *J* = 7.1 Hz, 6H).

^13^C{^1^H} NMR (101 MHz, CDCl_3_) δ 155.5, 154.3, 140.7, 137.7, 137.65, 136.4, 136.0, 128.5, 128.4, 128.3, 128.2, 127.9, 127.2, 127.0, 123.5, 123.3, 122.4, 121.7, 66.7, 57.1, 51.2, 50.8, 46.5, 46.4, 34.97, 34.7, 34.1, 31.65, 3.63, 29.7, 29.66, 29.62, 29.6, 29.55, 29.5, 11.6.

HRMS (ESI) *m/z*: [M + 2H]^2+^/2 Calcd for C_22.5_H_32.5_ON_1.5_ 339.7533, Found: 339.7541.

**2,4-di-tert-butyl-6-((((1S,2S)-2-((3-(tert-butyl)-2-hydroxy-5-(morpholinomethyl)benzyl)amino)-1,2-diphenylethyl)amino)methyl)phenol (L6)**

A tetrahydrofuran solution of intermediate **I6** was added to an anhydrous ethanol solution of **M3** under a nitrogen atmosphere. The reaction mixture was heated under reflux, with progress monitored by TLC. Upon completion, the mixture was cooled to room temperature and concentrated under reduced pressure. The residue was dissolved in anhydrous methanol and cooled to 0 °C. After purging with nitrogen, NaBH₄ was added portionwise, and the mixture was stirred at 0 °C for 30 min. The reaction was warmed to room temperature (monitored by TLC). After completion, the reaction was quenched with deionized water and extracted with dichloromethane (3 × 100 mL). The combined organic layers were washed with brine, dried over anhydrous sodium sulfate (Na₂SO₄), filtered, and concentrated. The crude product was purified by silica gel chromatography (PE/EA = 5:1) to afford **L6.** (1.68 g, 2.43 mmol, 97% yield). [α]_D_^25^ = -27.0 (c 0.10, CHCl_3_); m.p. 98-101 °C; ^1^H NMR (400 MHz, CDCl_3_) δ 7.21 (ddd, *J* = 6.9, 5.3, 2.2 Hz, 7H), 7.06 (d, *J* = 2.1 Hz, 1H), 6.94 (dt, *J* = 6.7, 2.3 Hz, 4H), 6.62 (dd, *J* = 8.3, 2.2 Hz, 2H), 4.00 (s, 2H), 3.80 (d, *J* = 13.3 Hz, 2H), 3.65 (t, *J* = 4.6 Hz, 4H), 3.60 (d, *J* = 13.3 Hz, 2H), 3.32 (s, 2H), 2.36 (t, *J* = 4.7 Hz, 4H), 1.42 (d, *J* = 3.2 Hz, 19H), 1.22 (s, 9H).

^13^C{^1^H} NMR (101 MHz, CDCl_3_) δ 156.0, 154.3, 140.7, 137.8, 137.7, 136.6, 136.1, 128.5, 128.2, 128.0, 127.5, 127.4, 123.6, 123.2, 122.5, 121.8, 67.1, 66.7, 66.6, 63.4, 53.6, 51.2, 50.8, 35.0, 34.7, 34.2, 31.7, 29.8, 29.7.

HRMS (ESI) *m/z*: [M + 2H]^2+^/2 Calcd for C_22.5_H_31.5_O_1.5_N_1.5_ 346.7429, Found: 346.7432.

**2,4-di-tert-butyl-6-((((1S,2S)-2-((5-(tert-butyl)-2-hydroxy-3-(morpholinomethyl)benzyl)amino)-1,2-diphenylethyl)amino)methyl)phenol (L7)**

A tetrahydrofuran solution of intermediate **I7** was added to an anhydrous ethanol solution of **M3** under a nitrogen atmosphere. The reaction mixture was heated under reflux, with progress monitored by TLC. Upon completion, the mixture was cooled to room temperature and concentrated under reduced pressure. The residue was dissolved in anhydrous methanol and cooled to 0 °C. After purging with nitrogen, NaBH₄ was added portionwise, and the mixture was stirred at 0 °C for 30 min. The reaction was warmed to room temperature (monitored by TLC). After completion, the reaction was quenched with deionized water and extracted with dichloromethane (3 × 100 mL). The combined organic layers were washed with brine, dried over anhydrous sodium sulfate (Na₂SO₄), filtered, and concentrated. The crude product was purified by silica gel chromatography (PE/EA = 5:1) to afford **L7.** (1.67 g, 2.43 mmol, 97% yield); [α]_D_^25^ = -15 (c 0.10, CHCl_3_); m.p. 90-92 °C; ^1^H NMR (400 MHz, CDCl_3_) δ 7.19 – 7.15 (m, 4H), 7.15 – 7.09 (m, 3H), 7.03 (dt, *J* = 7.7, 1.6 Hz, 4H), 6.95 (d, *J* = 2.4 Hz, 1H), 6.85 (d, *J* = 2.4 Hz, 1H), 6.68 (d, *J* = 2.4 Hz, 1H), 3.86 – 3.77 (m, 2H), 3.73 (dd, *J* = 13.2, 3.4 Hz, 2H), 3.67 (d, *J* = 5.1 Hz, 4H), 3.64 – 3.48 (m, 4H), 2.48 (t, *J* = 4.7 Hz, 4H), 1.42 (s, 9H), 1.22 (d, *J* = 2.3 Hz, 18H).

^13^C{^1^H} NMR (101 MHz, CDCl_3_) δ 154.5, 153.4, 141.4, 140.6, 140.3, 139.5, 135.7, 128.2, 128.15, 128.1, 128.0, 127.3, 127.1, 126.4, 125.7, 124.6, 123.3, 122.8, 122.7, 119.7, 69.0, 67.9, 66.8, 62.0, 53.0, 51.6, 48.3, 35.0, 34.1, 34.0, 31.7, 31.6, 29.8.

HRMS (ESI) *m/z*: [M + 2H]^2+^/2 Calcd for C_22.5_H_32.5_O_1.5_N_1.5_ 346.7429, Found: 346.7435.

**2,4-di-tert-butyl-6-((((1S,2S)-2-((3-(tert-butyl)-5-((diphenylamino)methyl)-2-hydroxybenzyl)amino)-1,2-diphenylethyl)amino)methyl)phenol (L8)**

A tetrahydrofuran solution of intermediate **I8** was added to an anhydrous ethanol solution of **M3** under a nitrogen atmosphere. The reaction mixture was heated under reflux, with progress monitored by TLC. Upon completion, the mixture was cooled to room temperature and concentrated under reduced pressure. The residue was dissolved in anhydrous methanol and cooled to 0 °C. After purging with nitrogen, NaBH₄ was added portionwise, and the mixture was stirred at 0 °C for 30 min. The reaction was warmed to room temperature (monitored by TLC). After completion, the reaction was quenched with deionized water and extracted with dichloromethane (3 × 100 mL). The combined organic layers were washed with brine, dried over anhydrous sodium sulfate (Na₂SO₄), filtered, and concentrated. The crude product was purified by silica gel chromatography (PE/EA = 5:1) to afford **L8.** (1.89 g, 2.45 mmol, 98% yield); [α]_D_^25^ = -12 (c 0.10, CHCl_3_); m.p. 87-89 °C; ^1^H NMR (400 MHz, CDCl_3_) δ 7.22 – 7.19 (m, 7H), 7.18 – 7.15 (m, 4H), 7.08 (d, *J* = 2.1 Hz, 1H), 7.02 – 6.98 (m, 4H), 6.92 – 6.88 (m, 3H), 6.85 (ddd, *J* = 7.8, 4.3, 2.1 Hz, 3H), 6.61 (dt, *J* = 7.1, 2.0 Hz, 2H), 4.82 (s, 2H), 3.97 (d, *J* = 7.1 Hz, 1H), 3.91 (d, *J* = 7.1 Hz, 1H), 3.79 (dd, *J* = 13.2, 2.2 Hz, 2H), 3.58 (d, *J* = 13.3 Hz, 1H), 3.49 (d, *J* = 13.4 Hz, 1H), 1.43 (d, *J* = 1.8 Hz, 9H), 1.36 (d, *J* = 1.8 Hz, 9H), 1.22 (d, *J* = 1.7 Hz, 9H).

^13^C{^1^H} NMR (101 MHz, CDCl_3_) δ 155.5, 154.3, 148.3, 140.7, 137.8, 137.7, 137.0, 136.0, 129.2, 128.4, 128.2, 127.9, 127.9, 124.9, 124.7, 123.5, 123.2, 122.5, 121.7, 121.2, 120.8, 66.5, 66.2, 56.1, 51.2, 50.7, 35.0, 34.8, 34.1, 31.7, 29.7, 29.6.

HRMS (ESI) *m/z*: [M + 2H]^2+^ Calcd for C_26.5_H_32.5_ON 387.7533, Found: 387.7540.

**3-((((1S,2S)-2-((3,5-di-tert-butyl-2-hydroxybenzyl)amino)-1,2-diphenylethyl)amino)methyl)-[1,1'-biphenyl]-2-ol (L9)**

A tetrahydrofuran solution of intermediate **I9** was added to an anhydrous ethanol solution of **M3** under a nitrogen atmosphere. The reaction mixture was heated under reflux, with progress monitored by TLC. Upon completion, the mixture was cooled to room temperature and concentrated under reduced pressure. The residue was dissolved in anhydrous methanol and cooled to 0 °C. After purging with nitrogen, NaBH₄ was added portionwise, and the mixture was stirred at 0 °C for 30 min. The reaction was warmed to room temperature (monitored by TLC). After completion, the reaction was quenched with deionized water and extracted with dichloromethane (3 × 100 mL). The combined organic layers were washed with brine, dried over anhydrous sodium sulfate (Na₂SO₄), filtered, and concentrated. The crude product was purified by silica gel chromatography (PE/EA = 5:1) to afford **L9.** (1.40 g, 2.28mmol, 91% yield); [α]_D_^25^ = -17.0 (c 0.10, CHCl_3_); m.p. 88-90 °C; ^1^H NMR (500 MHz, CDCl_3_) δ 7.57 – 7.51 (m, 2H), 7.34 (t, *J* = 7.6 Hz, 2H), 7.24 (t, *J* = 7.4 Hz, 1H), 7.15 (dd, *J* = 5.0, 1.9 Hz, 5H), 7.13 (dd, *J* = 6.5, 2.3 Hz, 3H), 6.88 (dd, *J* = 6.6, 2.9 Hz, 2H), 6.84 (dd, *J* = 6.5, 2.9 Hz, 2H), 6.72 – 6.68 (m, 2H), 6.53 (d, *J* = 2.4 Hz, 1H), 3.97 (d, *J* = 6.9 Hz, 1H), 3.93 (d, *J* = 6.9 Hz, 1H), 3.81 (d, *J* = 13.5 Hz, 1H), 3.69 (d, *J* = 13.4 Hz, 1H), 3.57 (d, *J* = 13.6 Hz, 1H), 3.50 (d, *J* = 13.4 Hz, 1H), 1.33 (s, 9H), 1.14 (s, 9H).

^13^C{^1^H} NMR (126 MHz, CDCl_3_) δ 154.9, 154.2, 140.7, 138.5, 137.5, 137.4, 136.0, 130.2, 129.5, 129.2, 128.52, 128.50, 128.3, 128.2, 128.1, 128.4, 128.01, 127.98, 126.9, 123.6, 123.3, 122.9, 121.6, 119.3, 66.7, 66.3, 51.0, 50.5, 35.0, 34.2, 31.7, 31.67, 29.7.

HRMS (ESI) *m/z*: [M + H]^+^ Calcd for C_42_H_49_O_2_N_2_ 613.3789, Found: 613.3792.

**3-((((1S,2S)-2-((3,5-di-tert-butyl-2-hydroxybenzyl)amino)-1,2-diphenylethyl)amino)methyl)-4'-methoxy-[1,1'-biphenyl]-2-ol (L10)**

A tetrahydrofuran solution of intermediate **I10** was added to an anhydrous ethanol solution of **M3** under a nitrogen atmosphere. The reaction mixture was heated under reflux, with progress monitored by TLC. Upon completion, the mixture was cooled to room temperature and concentrated under reduced pressure. The residue was dissolved in anhydrous methanol and cooled to 0 °C. After purging with nitrogen, NaBH₄ was added portionwise, and the mixture was stirred at 0 °C for 30 min. The reaction was warmed to room temperature (monitored by TLC). After completion, the reaction was quenched with deionized water and extracted with dichloromethane (3 × 100 mL). The combined organic layers were washed with brine, dried over anhydrous sodium sulfate (Na₂SO₄), filtered, and concentrated. The crude product was purified by silica gel chromatography (PE/EA = 5:1) to afford **L10.** (1.50 g, 2.33 mmol, 93% yield); [α]_D_^25^ = -9.0 (c 0.10, CHCl_3_); m.p. 74-76 °C; ^1^H NMR (400 MHz, CDCl_3_) δ 7.55 (dd, *J* = 8.7, 1.9 Hz, 2H), 7.21 (td, *J* = 4.2, 2.0 Hz, 8H), 6.96 (td, *J* = 6.1, 3.1 Hz, 4H), 6.94 – 6.90 (m, 2H), 6.80 – 6.71 (m, 2H), 6.65 – 6.59 (m, 1H), 4.05 – 3.97 (m, 2H), 3.88 (d, *J* = 13.7 Hz, 1H), 3.82 (d, *J* = 6.5 Hz, 3H), 3.80 – 3.72 (m, 1H), 3.66 – 3.53 (m, 2H), 1.42 (d, *J* = 5.7 Hz, 9H), 1.22 (d, *J* = 1.8 Hz, 9H).

^13^C{^1^H} NMR (101 MHz, CDCl_3_) δ 158.7, 154.8, 154.3, 140.8, 137.7, 137.6, 136.1, 131.0, 130.6, 130.0, 128.9, 128.6, 128.5, 128.3, 128.28, 128.2, 128.03, 128.00, 127.6, 123.6, 123.2, 122.9, 121.7, 119.3, 113.7, 66.8, 66.4, 55.4, 51.1, 50.5, 35.0, 34.2, 31.7, 29.8, 29.7.

HRMS (ESI) *m/z*: [M + H]^+^ Calcd for C_43_H_51_O_3_N_2_ 643.3894, Found:643.3899.

**3-((((1S,2S)-2-((3,5-di-tert-butyl-2-hydroxybenzyl)amino)-1,2-diphenylethyl)amino)methyl)-3',4'-dimethoxy-[1,1'-biphenyl]-2-ol (L11)**

A tetrahydrofuran solution of intermediate **I11** was added to an anhydrous ethanol solution of **M3** under a nitrogen atmosphere. The reaction mixture was heated under reflux, with progress monitored by TLC. Upon completion, the mixture was cooled to room temperature and concentrated under reduced pressure. The residue was dissolved in anhydrous methanol and cooled to 0 °C. After purging with nitrogen, NaBH₄ was added portionwise, and the mixture was stirred at 0 °C for 30 min. The reaction was warmed to room temperature (monitored by TLC). After completion, the reaction was quenched with deionized water and extracted with dichloromethane (3 × 100 mL). The combined organic layers were washed with brine, dried over anhydrous sodium sulfate (Na₂SO₄), filtered, and concentrated. The crude product was purified by silica gel chromatography (PE/EA = 5:1) to afford **L11.** (1.58 g, 2.35 mmol, 94% yield); [α]_D_^25^ = -6 (c 0.10, CHCl_3_); m.p. 90-92 °C; ^1^H NMR (400 MHz, CDCl_3_) δ 7.55 (d, *J* = 8.8 Hz, 2H), 7.26 – 7.21 (m, 6H), 7.20 (d, *J* = 2.5 Hz, 1H), 6.97 (dt, *J* = 5.9, 2.0 Hz, 4H), 6.93 (dd, *J* = 6.5, 3.0 Hz, 2H), 6.79 (d, *J* = 3.1 Hz, 1H), 6.61 (d, *J* = 2.4 Hz, 1H), 6.35 (d, *J* = 3.1 Hz, 1H), 4.06 – 3.95 (m, 2H), 3.88 (s, 1H), 3.85 (s, 3H), 3.77 (d, *J* = 13.3 Hz, 1H), 3.71 (s, 3H), 3.58 (dd, *J* = 13.5, 1.9 Hz, 2H), 1.41 (s, 9H), 1.22 (s, 9H).

^13^C{^1^H} NMR (101 MHz, CDCl_3_) δ 158.7, 154.2, 152.4, 148.6, 140.7, 137.6, 137.56, 136.0, 130.9, 130.5, 129.3, 128.5, 128.46, 128.3, 128.2, 128.0, 127.9, 123.6, 123.5, 123.2, 121.7, 114.8, 113.7, 113.6, 66.7, 66.4, 55.8, 55.3, 51.1, 50.6, 34.9, 34.1, 31.6, 29.7.

HRMS (ESI) *m/z*: [M + H]^+^ Calcd for C_44_H_53_O_4_N_2_ 673.4000, Found: 373.4005.

**3-((((1S,2S)-2-((3,5-di-tert-butyl-2-hydroxybenzyl)amino)-1,2-diphenylethyl)amino)methyl)-2',5,6'-trimethoxy-[1,1'-biphenyl]-2-ol (L12)**

A tetrahydrofuran solution of intermediate **I12** was added to an anhydrous ethanol solution of **M3** under a nitrogen atmosphere. The reaction mixture was heated under reflux, with progress monitored by TLC. Upon completion, the mixture was cooled to room temperature and concentrated under reduced pressure. The residue was dissolved in anhydrous methanol and cooled to 0 °C. After purging with nitrogen, NaBH₄ was added portionwise, and the mixture was stirred at 0 °C for 30 min. The reaction was warmed to room temperature (monitored by TLC). After completion, the reaction was quenched with deionized water and extracted with dichloromethane (3 × 100 mL). The combined organic layers were washed with brine, dried over anhydrous sodium sulfate (Na₂SO₄), filtered, and concentrated. The crude product was purified by silica gel chromatography (PE/EA = 5:1) to afford **L12.** (1.61 g, 2.40 mmol, 96% yield); [α]_D_^25^ = -9 (c 0.10, CHCl_3_); m.p. 83-85 °C; ^1^H NMR (400 MHz, CDCl_3_) δ 7.28 (d, *J* = 8.3 Hz, 1H), 7.21 (dd, *J* = 4.1, 2.2 Hz, 6H), 7.18 (d, *J* = 2.4 Hz, 1H), 6.95 (dd, *J* = 6.6, 2.9 Hz, 2H), 6.91 (dd, *J* = 6.5, 3.0 Hz, 2H), 6.70 – 6.66 (m, 2H), 6.65 (d, *J* = 1.8 Hz, 1H), 6.60 (d, *J* = 2.4 Hz, 1H), 6.43 (d, *J* = 3.1 Hz, 1H), 4.07 (d, *J* = 6.8 Hz, 1H), 3.99 (d, *J* = 6.8 Hz, 1H), 3.83 (d, *J* = 6.4 Hz, 1H), 3.76 (s, 3H), 3.74 – 3.72 (m, 3H), 3.72 (s, 1H), 3.67 (s, 3H), 3.63 – 3.56 (m, 2H), 1.41 (s, 9H), 1.21 (s, 9H).

^13^C{^1^H} NMR (101 MHz, CDCl_3_) δ 158.2, 158.15, 154.4, 151.9, 148.96, 140.6, 137.9, 137.8, 135.9, 129.1, 128.4, 128.3, 128.28, 127.9, 127.8, 123.5, 123.1, 122.6, 121.8, 116.4, 115.9, 114.1, 104.6, 104.6, 66.6, 66.5, 58.4, 56.2, 56.2, 55.7, 51.3, 50.2, 35.0, 34.1, 31.77, 29.7.

HRMS (ESI) *m/z*: [M + H]^+^ Calcd for C_45_H_55_O_5_N_2_ 703.4105, Found: 703.4111.

**3-((((1S,2S)-2-((3,5-di-tert-butyl-2-hydroxybenzyl)amino)-1,2-diphenylethyl)amino)methyl)-3',5'-dimethoxy-[1,1'-biphenyl]-2-ol (L13)**

A tetrahydrofuran solution of intermediate **I13** was added to an anhydrous ethanol solution of **M3** under a nitrogen atmosphere. The reaction mixture was heated under reflux, with progress monitored by TLC. Upon completion, the mixture was cooled to room temperature and concentrated under reduced pressure. The residue was dissolved in anhydrous methanol and cooled to 0 °C. After purging with nitrogen, NaBH₄ was added portionwise, and the mixture was stirred at 0 °C for 30 min. The reaction was warmed to room temperature (monitored by TLC). After completion, the reaction was quenched with deionized water and extracted with dichloromethane (3 × 100 mL). The combined organic layers were washed with brine, dried over anhydrous sodium sulfate (Na₂SO₄), filtered, and concentrated. The crude product was purified by silica gel chromatography (PE/EA = 5:1) to afford **L13.** (1.65 g, 2.45 mmol, 98% yield); [α]_D_^25^ = +19.0 (c 0.10, CHCl_3_); m.p. 105-107 °C; ^1^H NMR (400 MHz, CDCl_3_) δ 7.26 – 7.17 (m, 8H), 6.99 – 6.93 (m, 2H), 6.94 – 6.87 (m, 2H), 6.81 – 6.68 (m, 4H), 6.63 – 6.56 (m, 1H), 6.46 (q, *J* = 2.4 Hz, 1H), 4.06 (d, *J* = 6.7 Hz, 1H), 4.01 (d, *J* = 6.9 Hz, 1H), 3.92 – 3.85 (m, 1H), 3.84 – 3.80 (m, 6H), 3.76 (dd, *J* = 13.5, 2.3 Hz, 1H), 3.70 – 3.63 (m, 1H), 3.58 (dd, *J* = 13.4, 1.7 Hz, 1H), 1.40 (d, *J* = 2.6 Hz, 9H), 1.23 – 1.16 (m, 9H).

^13^C{^1^H} NMR (101 MHz, CDCl_3_) δ 160.5, 154.8, 154.2, 140.7, 140.5, 137.5, 137.4, 136.0, 130.0, 129.1, 128.5, 128.47, 128.3, 128.2, 128.1, 128.0, 123.6, 123.2, 123.0, 121.7, 119.2, 107.7, 99.5, 66.7, 66.2, 55.4, 51.0, 50.5, 34.9, 34.1, 31.7, 29.7.

HRMS (ESI) *m/z*: [M + H]^+^ Calcd for C_44_H_53_O_4_N_2_ 673.4000, Found: 673.4009.

**3',5'-di-tert-butyl-3-((((1S,2S)-2-((3,5-di-tert-butyl-2-hydroxybenzyl)amino)-1,2-diphenylethyl)amino)methyl)-[1,1'-biphenyl]-2-ol (L14)**

A tetrahydrofuran solution of intermediate **I14** was added to an anhydrous ethanol solution of **M3** under a nitrogen atmosphere. The reaction mixture was heated under reflux, with progress monitored by TLC. Upon completion, the mixture was cooled to room temperature and concentrated under reduced pressure. The residue was dissolved in anhydrous methanol and cooled to 0 °C. After purging with nitrogen, NaBH₄ was added portionwise, and the mixture was stirred at 0 °C for 30 min. The reaction was warmed to room temperature (monitored by TLC). After completion, the reaction was quenched with deionized water and extracted with dichloromethane (3 × 100 mL). The combined organic layers were washed with brine, dried over anhydrous sodium sulfate (Na₂SO₄), filtered, and concentrated. The crude product was purified by silica gel chromatography (PE/EA = 5:1) to afford **L14.**(1.78 g, 2.45 mmol, 98% yield); [α]_D_^25^ = -12 (c 0.10, CHCl_3_); m.p. 104-106 °C; ^1^H NMR (400 MHz, CDCl_3_) δ 7.45 (d, *J* = 1.9 Hz, 2H), 7.41 (t, *J* = 1.9 Hz, 1H), 7.29 – 7.21 (m, 7H), 7.19 (d, *J* = 2.4 Hz, 1H), 6.99 – 6.95 (m, 2H), 6.93 (dd, *J* = 6.9, 2.7 Hz, 2H), 6.81 – 6.75 (m, 2H), 6.60 (d, *J* = 2.3 Hz, 1H), 4.13 – 4.07 (m, 1H), 4.07 – 4.00 (m, 1H), 3.89 (d, *J* = 13.8 Hz, 1H), 3.76 (dd, *J* = 13.4, 2.1 Hz, 1H), 3.68 (dd, *J* = 13.5, 1.9 Hz, 1H), 3.60 (d, *J* = 13.5 Hz, 1H), 1.45 – 1.35 (m, 27H), 1.20 (d, *J* = 2.1 Hz, 9H).

^13^C{^1^H} NMR (101 MHz, CDCl_3_) δ 154.8, 154.3, 150.2, 140.7, 137.5, 137.4, 136.0, 130.3, 130.3, 128.4, 128.39, 128.3, 128.0, 127.99, 127.8, 124.0, 123.6, 123.2, 122.9, 121.7, 121.1, 119.2, 66.7, 66.1, 51.1, 50.5, 35.0, 34.95, 34.1, 31.7, 29.7.

HRMS (ESI) *m/z*: [M + H]^+^ Calcd for C_50_H_65_O_2_N_2_ 725.5041, Found: 725.5045.

**2,4-di-tert-butyl-6-((((1S,2S)-2-((3-(dibenzo[b,d]furan-4-yl)-2-hydroxybenzyl)amino)-1,2-diphenylethyl)amino)methyl)phenol (L15)**

A tetrahydrofuran solution of intermediate **I15** was added to an anhydrous ethanol solution of **M3** under a nitrogen atmosphere. The reaction mixture was heated under reflux, with progress monitored by TLC. Upon completion, the mixture was cooled to room temperature and concentrated under reduced pressure. The residue was dissolved in anhydrous methanol and cooled to 0 °C. After purging with nitrogen, NaBH₄ was added portionwise, and the mixture was stirred at 0 °C for 30 min. The reaction was warmed to room temperature (monitored by TLC). After completion, the reaction was quenched with deionized water and extracted with dichloromethane (3 × 100 mL). The combined organic layers were washed with brine, dried over anhydrous sodium sulfate (Na₂SO₄), filtered, and concentrated. The crude product was purified by silica gel chromatography (PE/EA = 5:1) to afford **L15.** (1.65 g, 2.35 mmol, 94% yield); [α]_D_^25^ = +9.0 (c 0.10, CHCl_3_); m.p. 100-102 °C; ^1^H NMR (400 MHz, CDCl_3_) δ 7.94 – 7.84 (m, 2H), 7.58 (dd, *J* = 7.6, 1.3 Hz, 1H), 7.47 (dt, *J* = 8.2, 0.9 Hz, 1H), 7.42 (dd, *J* = 5.2, 4.2 Hz, 1H), 7.38 – 7.31 (m, 2H), 7.25 (td, *J* = 7.5, 1.0 Hz, 1H), 7.15 (ddd, *J* = 6.4, 4.2, 2.7 Hz, 6H), 7.11 (d, *J* = 2.4 Hz, 1H), 6.89 (dd, *J* = 6.5, 3.0 Hz, 2H), 6.86 – 6.80 (m, 4H), 6.51 (d, *J* = 2.4 Hz, 1H), 4.04 (d, *J* = 6.8 Hz, 1H), 3.98 – 3.84 (m, 2H), 3.66 (dd, *J* = 18.0, 13.5 Hz, 2H), 3.50 (d, *J* = 13.4 Hz, 1H), 1.31 (s, 9H), 1.12 (s, 9H).

^13^C{^1^H} NMR (101 MHz, CDCl_3_) δ 156.2, 155.4, 154.3, 154.0, 140.7, 137.4, 137.3, 136.0, 131.0, 129.1, 128.7, 128.5, 128.3, 128.2, 128.04, 128.00, 127.0, 124.6, 124.5, 124.0, 123.6, 123.2, 123.0, 122.9, 122.6, 122.57, 121.6, 120.7, 119.6, 119.1, 111.8, 77.3, 66.4, 66.1, 51.0, 50.4, 34.9, 34.1, 31.6, 29.6, 27.0.

HRMS (ESI) *m/z*: [M + H]^+^ Calcd for C_48_H_51_O_3_N_2_ 703.3894, Found: 703.3897.

**2,4-di-tert-butyl-6-((((1S,2S)-2-((2-hydroxy-3-(naphthalen-1-yl)benzyl)amino)-1,2-diphenylethyl)amino)methyl)phenol (L16)**

A tetrahydrofuran solution of intermediate **I16** was added to an anhydrous ethanol solution of **M3** under a nitrogen atmosphere. The reaction mixture was heated under reflux, with progress monitored by TLC. Upon completion, the mixture was cooled to room temperature and concentrated under reduced pressure. The residue was dissolved in anhydrous methanol and cooled to 0 °C. After purging with nitrogen, NaBH₄ was added portionwise, and the mixture was stirred at 0 °C for 30 min. The reaction was warmed to room temperature (monitored by TLC). After completion, the reaction was quenched with deionized water and extracted with dichloromethane (3 × 100 mL). The combined organic layers were washed with brine, dried over anhydrous sodium sulfate (Na₂SO₄), filtered, and concentrated. The crude product was purified by silica gel chromatography (PE/EA = 5:1) to afford **L16.** (1.61 g, 2.43 mmol, 97% yield); [α]_D_^25^ = +16.0 (c 0.10, CHCl_3_); m.p. 92-94 °C; ^1^H NMR (400 MHz, CDCl_3_) δ 7.90 – 7.83 (m, 2H), 7.79 – 7.65 (m, 1H), 7.55 – 7.49 (m, 1H), 7.49 – 7.31 (m, 3H), 7.23 – 7.16 (m, 8H), 6.93 (ddd, *J* = 9.7, 6.5, 3.1 Hz, 2H), 6.90 – 6.80 (m, 4H), 6.58 (dd, *J* = 4.6, 2.4 Hz, 1H), 4.07 (dd, *J* = 13.9, 6.9 Hz, 1H), 3.99 – 3.89 (m, 2H), 3.71 (ddd, *J* = 13.7, 10.9, 5.4 Hz, 2H), 3.56 (dd, *J* = 13.3, 10.7 Hz, 1H), 1.40 (d, *J* = 5.1 Hz, 9H), 1.20 (d, *J* = 1.9 Hz, 9H).

^13^C{^1^H} NMR (101 MHz, CDCl_3_) δ 155.4, 155.3, 154.35, 154.29, 140.8, 140.7, 137.60, 137.55, 137.55, 137.51, 136.8, 136.7, 136.1, 133.79, 133.77, 132.22, 132.19, 131.4, 131.3, 128.5, 128.45, 128.41, 128.4, 128.33, 128.30, 128.22, 128.18, 128.15, 128.1, 128.0, 127.9, 127.8, 127.8, 127.6, 126.7, 126.6, 125.9, 125.8, 125.8, 125.7, 125.6, 123.6, 123.3, 123.2, 122.9, 122.7, 121.7, 119.2, 119.1, 66.5, 66.5, 66.3, 66.2, 51.1, 51.0, 50.4, 50.3, 35.0, 34.2, 31.7, 29.8, 29.7, 27.0.

HRMS (ESI) *m/z*: [M + H]^+^ Calcd for C_46_H_51_O_2_N_2_ 663.3945, Found: 663.3943.

**2,4-di-tert-butyl-6-((((1S,2S)-2-((2-hydroxy-5-methoxy-3-(naphthalen-1-yl)benzyl)amino)-1,2-diphenylethyl)amino)methyl)phenol (L17)**

A tetrahydrofuran solution of intermediate **I17** was added to an anhydrous ethanol solution of **M3** under a nitrogen atmosphere. The reaction mixture was heated under reflux, with progress monitored by TLC. Upon completion, the mixture was cooled to room temperature and concentrated under reduced pressure. The residue was dissolved in anhydrous methanol and cooled to 0 °C. After purging with nitrogen, NaBH₄ was added portionwise, and the mixture was stirred at 0 °C for 30 min. The reaction was warmed to room temperature (monitored by TLC). After completion, the reaction was quenched with deionized water and extracted with dichloromethane (3 × 100 mL). The combined organic layers were washed with brine, dried over anhydrous sodium sulfate (Na₂SO₄), filtered, and concentrated. The crude product was purified by silica gel chromatography (PE/EA = 5:1) to afford **L17.** (1.63 g, 2.35 mmol, 94%yield); [α]_D_^25^ = +11.0 (c 0.10, CHCl_3_); m.p. 98-100 °C; ^1^H NMR (400 MHz, CDCl_3_) δ 7.91 – 7.84 (m, 2H), 7.81 – 7.66 (m, 1H), 7.58 – 7.47 (m, 2H), 7.46 – 7.36 (m, 2H), 7.24 – 7.16 (m, 7H), 6.98 – 6.91 (m, 2H), 6.88 (td, *J* = 5.9, 2.4 Hz, 2H), 6.78 (t, *J* = 3.0 Hz, 1H), 6.59 (dd, *J* = 4.2, 2.4 Hz, 1H), 6.50 (dd, *J* = 3.1, 1.6 Hz, 1H), 4.07 (dd, *J* = 13.8, 6.9 Hz, 1H), 3.99 – 3.89 (m, 2H), 3.74 (dd, *J* = 13.1, 6.7 Hz, 1H), 3.70 (s, 3H), 3.65 (dd, *J* = 13.7, 5.5 Hz, 1H), 3.56 (dd, *J* = 13.4, 9.8 Hz, 1H), 1.39 (d, *J* = 5.0 Hz, 9H), 1.20 (d, *J* = 2.1 Hz, 10H).

^13^C{^1^H} NMR (101 MHz, CDCl_3_) δ 154.3, 154.24, 152.25, 152.19, 149.03, 149.00, 140.69, 140.66, 137.57, 137.5, 137.46, 136.7, 136.6, 136.0, 133.70, 133.69, 132.01, 131.99, 128.54, 128.52, 128.46, 128.44, 128.36, 128.33, 128.28, 128.26, 128.2, 128.0, 127.97, 127.95, 127.92, 127.89, 127.6, 127.4, 126.6, 126.5, 126.0, 125.9, 125.8, 125.7, 125.5, 123.7, 123.5, 123.2, 123.17, 121.7, 115.96, 115.92, 114.53, 114.52, 77.3, 66.4, 66.3, 66.2, 55.8, 51.08, 51.01, 50.4, 50.33, 35.0, 34.1, 31.7, 29.7, 29.66, 22.7, 14.2.

HRMS (ESI) *m/z*: [M + H]^+^ Calcd for C_47_H_53_O_3_N_2_ 693.4051, Found: 693.4055.

**2,4-di-tert-butyl-6-((((1S,2S)-2-((5-(tert-butyl)-2-hydroxy-3-(naphthalen-1-yl)benzyl)amino)-1,2-diphenylethyl)amino)methyl)phenol (L18)**

A tetrahydrofuran solution of intermediate **I18** was added to an anhydrous ethanol solution of **M3** under a nitrogen atmosphere. The reaction mixture was heated under reflux, with progress monitored by TLC. Upon completion, the mixture was cooled to room temperature and concentrated under reduced pressure. The residue was dissolved in anhydrous methanol and cooled to 0 °C. After purging with nitrogen, NaBH₄ was added portionwise, and the mixture was stirred at 0 °C for 30 min. The reaction was warmed to room temperature (monitored by TLC). After completion, the reaction was quenched with deionized water and extracted with dichloromethane (3 × 100 mL). The combined organic layers were washed with brine, dried over anhydrous sodium sulfate (Na₂SO₄), filtered, and concentrated. The crude product was purified by silica gel chromatography (PE/EA = 5:1) to afford **L18.** (1.72 g, 2.40 mmol, 96% yield); [α]_D_^25^ = -14.0 (c 0.10, CHCl_3_); m.p. 93-95 °C; ^1^H NMR (400 MHz, CDCl_3_) δ 7.85 – 7.76 (m, 2H), 7.73 – 7.59 (m, 1H), 7.51 – 7.45 (m, 1H), 7.41 – 7.36 (m, 2H), 7.32 (ddd, *J* = 8.2, 6.7, 1.4 Hz, 1H), 7.18 – 7.09 (m, 8H), 6.88 (ddd, *J* = 11.4, 6.6, 3.0 Hz, 2H), 6.84 – 6.69 (m, 3H), 6.51 (t, *J* = 2.9 Hz, 1H), 4.02 (t, *J* = 7.2 Hz, 1H), 3.91 (dd, *J* = 6.8, 1.8 Hz, 1H), 3.84 (dd, *J* = 13.6, 4.6 Hz, 1H), 3.69 – 3.59 (m, 2H), 3.49 (dd, *J* = 13.4, 7.1 Hz, 1H), 1.31 (d, *J* = 5.4 Hz, 9H), 1.18 (s, 9H), 1.12 (d, *J* = 1.6 Hz, 9H).

^13^C{^1^H} NMR (101 MHz, CDCl_3_) δ 153.2, 153.1, 151.7, 140.6, 140.5, 139.62, 139.60, 136.5, 136.4, 136.4, 136.2, 136.1, 134.9, 132.7, 131.13, 131.9, 127.35, 127.31, 127.27, 127.21, 127.18, 127.1, 126.9, 126.8, 126.7, 126.62, 126.59, 126.5, 126.3, 126.2, 125.63, 125.58, 124.7, 124.6, 124.5, 124.4, 124.2, 124.1, 122.4, 122.12, 122.10, 121.0, 120.9, 120.6, 65.7, 65.6, 65.3, 65.2, 50.0, 49.95, 49.8, 33.9, 33.04, 33.00, 30.6, 30.5, 28.64, 28.60, 28.58.

HRMS (ESI) *m/z*: [M + H]^+^ Calcd for C_50_H_59_O_2_N_2_ 719.4571, Found: 719.4575.

**2,4-di-tert-butyl-6-((((1S,2S)-2-((2-hydroxy-3,5-di(naphthalen-1-yl)benzyl)amino)-1,2-diphenylethyl)amino)methyl)phenol (L19)**

A tetrahydrofuran solution of intermediate **I19** was added to an anhydrous ethanol solution of **M3** under a nitrogen atmosphere. The reaction mixture was heated under reflux, with progress monitored by TLC. Upon completion, the mixture was cooled to room temperature and concentrated under reduced pressure. The residue was dissolved in anhydrous methanol and cooled to 0 °C. After purging with nitrogen, NaBH₄ was added portionwise, and the mixture was stirred at 0 °C for 30 min. The reaction was warmed to room temperature (monitored by TLC). After completion, the reaction was quenched with deionized water and extracted with dichloromethane (3 × 100 mL). The combined organic layers were washed with brine, dried over anhydrous sodium sulfate (Na₂SO₄), filtered, and concentrated. The crude product was purified by silica gel chromatography (PE/EA = 5:1) to afford **L19.** (1.91 g, 2.43 mmol, 97% yield); [α]_D_^25^ = 8 (c 0.11, CHCl_3_); m.p. 131-133 °C; ^1^H NMR (400 MHz, CDCl_3_) δ 8.07 – 7.99 (m, 1H), 7.88 (dddd, *J* = 18.0, 11.1, 6.1, 2.6 Hz, 4H), 7.77 (dt, *J* = 8.0, 1.1 Hz, 1H), 7.61 – 7.52 (m, 2H), 7.50 – 7.44 (m, 3H), 7.44 – 7.39 (m, 3H), 7.38 (d, *J* = 2.2 Hz, 1H), 7.23 – 7.15 (m, 7H), 7.05 (t, *J* = 1.8 Hz, 1H), 6.97 (dq, *J* = 8.2, 2.9 Hz, 2H), 6.90 (p, *J* = 3.0 Hz, 2H), 6.61 (dd, *J* = 4.9, 2.4 Hz, 1H), 4.13 (dd, *J* = 10.3, 7.0 Hz, 1H), 4.05 – 3.93 (m, 2H), 3.84 – 3.70 (m, 2H), 3.59 (dd, *J* = 13.4, 11.5 Hz, 1H), 1.41 (d, *J* = 5.3 Hz, 9H), 1.21 (d, *J* = 2.1 Hz, 9H).

^13^C{^1^H} NMR (101 MHz, CDCl_3_) δ 154.69, 154.3, 154.2, 140.81, 140.79, 139.9, 137.6, 137.5, 137.4, 136.6, 136.5, 136.1, 134.0, 133.8, 132.94, 132.85, 132.2, 131.8, 131.6, 131.5, 129.93, 129.90, 128.51, 128.47, 128.39, 128.37, 128.33, 128.26, 128.2, 128.1, 128.05, 128.02, 128.00, 127.94, 127.90, 127.86, 127.74, 127.3, 127.0, 126.7, 126.6, 126.2, 126.18, 126.0, 125.8, 125.76, 125.7, 125.68, 125.54, 125.45, 123.6, 123.3, 123.26, 122.7, 122.6, 121.7, 66.9, 66.7, 66.4, 66.3, 51.08, 51.00, 50.55, 50.52, 35.0, 34.2, 31.7, 29.7.

HRMS (ESI) *m/z*: [M + H]^+^ Calcd for C_56_H_57_O_2_N_2_ 789.4415, Found: 789.4419.

**2,4-di-tert-butyl-6-((((1S,2S)-2-((2-hydroxy-3-(naphthalen-2-yl)benzyl)amino)-1,2-diphenylethyl)amino)methyl)phenol (L20)**

A tetrahydrofuran solution of intermediate **I20** was added to an anhydrous ethanol solution of **M3** under a nitrogen atmosphere. The reaction mixture was heated under reflux, with progress monitored by TLC. Upon completion, the mixture was cooled to room temperature and concentrated under reduced pressure. The residue was dissolved in anhydrous methanol and cooled to 0 °C. After purging with nitrogen, NaBH₄ was added portionwise, and the mixture was stirred at 0 °C for 30 min. The reaction was warmed to room temperature (monitored by TLC). After completion, the reaction was quenched with deionized water and extracted with dichloromethane (3 × 100 mL). The combined organic layers were washed with brine, dried over anhydrous sodium sulfate (Na₂SO₄), filtered, and concentrated. The crude product was purified by silica gel chromatography (PE/EA = 5:1) to afford **L20.** (1.59 g, 2.40 mmol, 96% yield); [α]_D_^25^ = -4.0 (c 0.10, CHCl_3_); m.p. 100-103 °C; ^1^H NMR (400 MHz, CDCl_3_) δ 7.93 – 7.84 (m, 2H), 7.80 – 7.64 (m, 1H), 7.55 (td, *J* = 7.7, 2.5 Hz, 1H), 7.51 – 7.40 (m, 3H), 7.28 – 7.18 (m, 8H), 6.94 (ddd, *J* = 9.7, 6.5, 3.0 Hz, 2H), 6.86 (ddd, *J* = 14.7, 7.2, 2.7 Hz, 4H), 6.58 (dd, *J* = 4.3, 2.4 Hz, 1H), 4.08 (dd, *J* = 13.0, 6.9 Hz, 1H), 4.02 – 3.91 (m, 2H), 3.80 – 3.66 (m, 2H), 3.56 (dd, *J* = 13.3, 9.3 Hz, 1H), 1.39 (d, *J* = 4.9 Hz, 9H), 1.20 (d, *J* = 1.9 Hz, 9H).

^13^C{^1^H} NMR (101 MHz, CDCl_3_) δ 155.34, 155.32, 154.30, 154.25, 140.71, 140.68, 137.5, 137.38, 137.36, 137.3, 136.8, 136.7, 136.0, 133.72, 133.70, 132.14, 132.10, 131.4, 131.3, 128.5, 128.39, 128.37, 128.3, 128.28, 128.2, 128.13, 128.11, 128.04, 128.02, 128.00, 127.83, 127.80, 127.7, 127.5, 126.6, 126.5, 125.9, 125.8, 125.7, 125.66, 125.53, 123.56, 123.23, 123.20, 122.8, 122.62, 121.63, 119.12, 119.05, 66.38, 66.36, 66.2, 66.1, 51.1, 51.0, 50.32, 50.27, 35.0, 34.2, 31.7, 31.67, 29.69, 29.67.

HRMS (ESI) *m/z*: [M + H]^+^ Calcd for C_46_H_51_O_2_N_2_ 663.3945, Found: 663.3947.

**2,4-di-tert-butyl-6-((((1S,2S)-2-((2-hydroxy-3-(phenanthren-9-yl)benzyl)amino)-1,2-diphenylethyl)amino)methyl)phenol (L21)**

A tetrahydrofuran solution of intermediate **I21** was added to an anhydrous ethanol solution of **M3** under a nitrogen atmosphere. The reaction mixture was heated under reflux, with progress monitored by TLC. Upon completion, the mixture was cooled to room temperature and concentrated under reduced pressure. The residue was dissolved in anhydrous methanol and cooled to 0 °C. After purging with nitrogen, NaBH₄ was added portionwise, and the mixture was stirred at 0 °C for 30 min. The reaction was warmed to room temperature (monitored by TLC). After completion, the reaction was quenched with deionized water and extracted with dichloromethane (3 × 100 mL). The combined organic layers were washed with brine, dried over anhydrous sodium sulfate (Na₂SO₄), filtered, and concentrated. The crude product was purified by silica gel chromatography (PE/EA = 5:1) to afford **L21.** (1.72 g, 2.43 mmol, 97% yield); [α]_D_^25^ = -12.0 (c 0.10, CHCl_3_); m.p. 111-113 °C; ^1^H NMR (400 MHz, CDCl_3_) δ 8.82 – 8.65 (m, 2H), 7.95 – 7.85 (m, 1H), 7.84 – 7.75 (m, 1H), 7.70 (d, *J* = 8.5 Hz, 1H), 7.68 – 7.54 (m, 4H), 7.27 (dt, *J* = 7.3, 2.2 Hz, 1H), 7.25 – 7.13 (m, 7H), 6.98 – 6.91 (m, 3H), 6.90 – 6.80 (m, 3H), 6.58 (dd, *J* = 7.6, 2.4 Hz, 1H), 4.13 – 4.05 (m, 1H), 4.01 – 3.91 (m, 2H), 3.72 (dt, *J* = 13.4, 5.2 Hz, 2H), 3.56 (t, *J* = 12.9 Hz, 1H), 1.38 (s, 9H), 1.19 (d, *J* = 5.8 Hz, 9H).

^13^C{^1^H} NMR (101 MHz, CDCl_3_) δ 155.5, 154.3, 154.2, 140.7, 140.68, 137.4, 137.3, 137.27, 136.0, 135.5, 131.9, 131.4, 131.35, 131.3, 131.2, 130.5, 130.47, 130.3, 128.8, 128.7, 128.5, 128.47, 128.45, 128.4,0 128.36, 128.3, 128.2, 128.1, 128.07, 128.03, 127.4, 127.2, 126.6, 126.5, 126.44, 126.39, 126.36, 123.5, 123.21, 123.19, 122.9, 122.8, 122.7, 122.61, 122.57, 121.6, 119.3, 119.2, 66.4, 66.3, 66.2, 66.0, 51.1, 51.0, 50.3, 50.2, 34.9, 34.1, 31.6, 31.6, 29.7, 29.6.

HRMS (ESI) *m/z*: [M + H]^+^ Calcd for C_50_H_53_O_2_N_2_ 713.4102, Found: 713.4101.

**2,4-di-tert-butyl-6-((((1S,2S)-2-((2-hydroxy-5-methoxy-3-(phenanthren-9-yl)benzyl)amino)-1,2-diphenylethyl)amino)methyl)phenol (L22)**

A tetrahydrofuran solution of intermediate **I22** was added to an anhydrous ethanol solution of **M3** under a nitrogen atmosphere. The reaction mixture was heated under reflux, with progress monitored by TLC. Upon completion, the mixture was cooled to room temperature and concentrated under reduced pressure. The residue was dissolved in anhydrous methanol and cooled to 0 °C. After purging with nitrogen, NaBH₄ was added portionwise, and the mixture was stirred at 0 °C for 30 min. The reaction was warmed to room temperature (monitored by TLC). After completion, the reaction was quenched with deionized water and extracted with dichloromethane (3 × 100 mL). The combined organic layers were washed with brine, dried over anhydrous sodium sulfate (Na₂SO₄), filtered, and concentrated. The crude product was purified by silica gel chromatography (PE/EA = 5:1) to afford **L22.** (1.76 g, 2.38 mmol, 95% yield); [α]_D_^25^ = +8.0 (c 0.10, CHCl_3_); m.p. 127-129 °C; ^1^H NMR (400 MHz, CDCl_3_) δ 8.78 – 8.69 (m, 2H), 7.93 – 7.87 (m, 1H), 7.83 – 7.76 (m, 1H), 7.72 (t, *J* = 4.2 Hz, 1H), 7.68 – 7.60 (m, 3H), 7.55 – 7.47 (m, 1H), 7.25 – 7.21 (m, 3H), 7.20 – 7.15 (m, 4H), 7.04 – 6.95 (m, 2H), 6.92 (dq, *J* = 6.0, 2.9 Hz, 2H), 6.84 (dt, *J* = 3.0, 1.4 Hz, 1H), 6.61 – 6.52 (m, 2H), 4.12 (t, *J* = 7.8 Hz, 1H), 4.02 (dd, *J* = 7.5, 3.1 Hz, 1H), 3.96 (dd, *J* = 13.7, 7.9 Hz, 1H), 3.79 – 3.73 (m, 1H), 3.71 (s, 3H), 3.67 (d, *J* = 1.7 Hz, 1H), 3.60 (dd, *J* = 13.4, 9.6 Hz, 1H), 1.41 – 1.33 (m, 9H), 1.18 (dd, *J* = 5.8, 1.2 Hz, 9H).

^13^C{^1^H} NMR (101 MHz, CDCl_3_) δ 154.0, 153.9, 152.53, 152.50, 148.8, 140.9, 136.9, 136.3, 135.1, 131.7, 131.2, 131.1, 130.6, 130.5, 130.4, 128.85, 128.80, 128.6, 128.5, 128.42, 128.39, 128.3, 128.21, 128.18, 128.14, 128.11, 127.3, 127.1, 126.75, 126.70, 126.64, 126.61, 126.6, 123.9, 123.4, 123.2, 123.0, 122.9, 122.6, 121.7, 121.6, 116.1, 114.9, 114.8, 66.4, 66.3, 66.2, 66.0, 55.9, 50.7, 50.6, 49.9, 34.9, 34.1, 31.71, 31.65, 31.6, 31.5, 29.8, 29.71, 29.68.

HRMS (ESI) *m/z*: [M + H]^+^ Calcd for C_51_H_55_O_3_N_2_ 743.4207, Found: 743.4213.

**2,4-di-tert-butyl-6-((((1S,2S)-2-((5-(tert-butyl)-2-hydroxy-3-(phenanthren-9-yl)benzyl)amino)-1,2-diphenylethyl)amino)methyl)phenol (L23)**

A tetrahydrofuran solution of intermediate **I23** was added to an anhydrous ethanol solution of **M3** under a nitrogen atmosphere. The reaction mixture was heated under reflux, with progress monitored by TLC. Upon completion, the mixture was cooled to room temperature and concentrated under reduced pressure. The residue was dissolved in anhydrous methanol and cooled to 0 °C. After purging with nitrogen, NaBH₄ was added portionwise, and the mixture was stirred at 0 °C for 30 min. The reaction was warmed to room temperature (monitored by TLC). After completion, the reaction was quenched with deionized water and extracted with dichloromethane (3 × 100 mL). The combined organic layers were washed with brine, dried over anhydrous sodium sulfate (Na₂SO₄), filtered, and concentrated. The crude product was purified by silica gel chromatography (PE/EA = 5:1) to afford **L23.** (1.87 g, 2.43 mmol, 97% yield); [α]_D_^25^ = -6.0 (c 0.10, CHCl_3_); m.p. 128-131 °C; ^1^H NMR (400 MHz, CDCl_3_) δ 8.26 (dd, *J* = 7.8, 3.3 Hz, 1H), 8.19 – 8.13 (m, 2H), 8.11 – 8.03 (m, 4H), 8.02 – 7.97 (m, 2H), 7.35 (d, *J* = 2.4 Hz, 1H), 7.25 – 7.15 (m, 7H), 6.96 (tt, *J* = 6.4, 3.1 Hz, 3H), 6.88 (h, *J* = 2.4 Hz, 2H), 6.57 (t, *J* = 2.4 Hz, 1H), 4.11 (dd, *J* = 14.2, 6.8 Hz, 1H), 3.99 (q, *J* = 2.9 Hz, 1H), 3.98 – 3.89 (m, 1H), 3.83 – 3.76 (m, 1H), 3.75 – 3.69 (m, 1H), 3.56 (dd, *J* = 13.3, 9.4 Hz, 1H), 1.39 (d, *J* = 10.4 Hz, 9H), 1.30 (s, 9H), 1.18 (s, 9H).

^13^C{^1^H} NMR (101 MHz, CDCl_3_) δ 154.19, 154.16, 152.8, 141.9, 140.8, 137.33, 137.27, 136.0, 131.5, 131.17, 131.15, 130.8, 130.0, 129.2, 128.9, 128.8, 128.5, 128.4, 128.35, 128.29, 128.2, 128.1, 128.03, 128.00, 127.74, 127.66, 127.6, 127.32, 127.28, 127.2, 126.1, 125.9, 125.5, 125.04, 125.00, 124.97, 124.95, 124.87, 124.8, 124.7, 123.6, 123.3, 122.0, 121.69, 121.67, 66.8, 66.7, 66.2, 66.1, 50.9, 34.94, 34.92, 34.2, 34.1, 31.8, 31.7, 31.6, 29.7, 29.68, 29.65.

HRMS (ESI) *m/z*: [M + H]^+^ Calcd for C_54_H_61_O_2_N_2_ 769.4728, Found: 769.4733.

**2,4-di-tert-butyl-6-((((1S,2S)-2-((2-hydroxy-3-(pyren-1-yl)benzyl)amino)-1,2-diphenylethyl)amino)methyl)phenol (L24)**

A tetrahydrofuran solution of intermediate **I24** was added to an anhydrous ethanol solution of **M3** under a nitrogen atmosphere. The reaction mixture was heated under reflux, with progress monitored by TLC. Upon completion, the mixture was cooled to room temperature and concentrated under reduced pressure. The residue was dissolved in anhydrous methanol and cooled to 0 °C. After purging with nitrogen, NaBH₄ was added portionwise, and the mixture was stirred at 0 °C for 30 min. The reaction was warmed to room temperature (monitored by TLC). After completion, the reaction was quenched with deionized water and extracted with dichloromethane (3 × 100 mL). The combined organic layers were washed with brine, dried over anhydrous sodium sulfate (Na₂SO₄), filtered, and concentrated. The crude product was purified by silica gel chromatography (PE/EA = 5:1) to afford **L24.** (1.75 g, 2.38 mmol, 95% yield); [α]_D_^25^ = +9.0 (c 0.10, CHCl_3_); m.p. 107-110 °C; ^1^H NMR (400 MHz, CDCl_3_) δ 8.17 (dd, *J* = 7.9, 1.1 Hz, 1H), 8.11 – 8.04 (m, 2H), 8.03 – 7.96 (m, 3H), 7.96 – 7.89 (m, 2H), 7.26 (dt, *J* = 7.2, 1.9 Hz, 1H), 7.15 (td, *J* = 5.2, 3.0 Hz, 6H), 7.10 (dd, *J* = 4.0, 2.5 Hz, 1H), 6.92 – 6.84 (m, 4H), 6.80 (ddt, *J* = 6.0, 4.5, 2.3 Hz, 2H), 6.49 (t, *J* = 2.6 Hz, 1H), 4.08 – 3.99 (m, 1H), 3.96 – 3.87 (m, 2H), 3.73 – 3.58 (m, 2H), 3.48 (dd, *J* = 13.4, 11.2 Hz, 1H), 1.31 (d, *J* = 8.8 Hz, 9H), 1.10 (s, 9H).

^13^C{^1^H} NMR (101 MHz, CDCl_3_) δ 155.53, 155.50, 154.3, 154.2, 140.69, 140.66, 137.4, 137.32, 137.25, 136.0, 134.19, 134.17, 131.82, 131.73, 131.5, 131.2, 131.1, 130.80, 130.77, 129.23, 129.20, 128.52, 128.47, 128.45, 128.41, 128.38, 128.35, 128.24, 128.21, 128.17, 128.04, 128.01, 127.98, 127.6, 127.54, 127.29, 127.27, 127.25, 127.11, 126.05, 126.0, 125.84, 125.82, 125.03, 124.96, 124.92, 124.88, 124.8, 124.7, 123.5, 123.22, 123.18, 122.9, 122.8, 121.6, 119.14, 119.09, 77.3, 66.5, 66.4, 66.2, 66.1, 51.1, 51.0, 50.4, 34.9, 34.1, 31.6, 29.8, 29.7, 29.6.

HRMS (ESI) *m/z*: [M + H]^+^ Calcd for C_52_H_53_O_2_N_2_ 737.4102, Found: 737.4106.

**2-((((1S,2S)-2-((3-(anthracen-9-yl)-2-hydroxybenzyl)amino)-1,2-diphenylethyl)amino)methyl)-4,6-di-tert-butylphenol (L25)**

A tetrahydrofuran solution of intermediate **I25** was added to an anhydrous ethanol solution of **M3** under a nitrogen atmosphere. The reaction mixture was heated under reflux, with progress monitored by TLC. Upon completion, the mixture was cooled to room temperature and concentrated under reduced pressure. The residue was dissolved in anhydrous methanol and cooled to 0 °C. After purging with nitrogen, NaBH₄ was added portionwise, and the mixture was stirred at 0 °C for 30 min. The reaction was warmed to room temperature (monitored by TLC). After completion, the reaction was quenched with deionized water and extracted with dichloromethane (3 × 100 mL). The combined organic layers were washed with brine, dried over anhydrous sodium sulfate (Na₂SO₄), filtered, and concentrated. The crude product was purified by silica gel chromatography (PE/EA = 5:1) to afford **L25.** (1.62 g, 2.28 mmol, 91% yield); [α]_D_^25^ = +33.0 (c 0.10, CHCl_3_); m.p. 118-120 °C; ^1^H NMR (400 MHz, CDCl_3_) δ 8.50 (s, 1H), 8.11 – 7.98 (m, 2H), 7.79 – 7.72 (m, 1H), 7.69 (d, *J* = 8.8 Hz, 1H), 7.43 (qd, *J* = 6.3, 3.5 Hz, 4H), 7.38 – 7.33 (m, 1H), 7.24 – 7.20 (m, 3H), 7.17 (d, *J* = 5.9 Hz, 4H), 6.98 – 6.88 (m, 4H), 6.83 (dd, *J* = 7.2, 2.4 Hz, 2H), 6.56 (d, *J* = 2.3 Hz, 1H), 4.12 – 3.99 (m, 2H), 3.93 (d, *J* = 7.0 Hz, 1H), 3.79 – 3.64 (m, 2H), 3.52 (d, *J* = 13.3 Hz, 1H), 1.39 (s, 9H), 1.19 (s, 9H).

^13^C{^1^H} NMR (101 MHz, CDCl_3_) δ 156.0, 154.3, 140.6, 137.5, 137.4, 136.0, 133.4, 132.2, 131.7, 130.5, 128.7, 128.64, 128.57, 128.47, 128.42, 128.38, 128.2, 128.1, 128.0, 127.97, 126.9, 126.83, 126.77, 125.8, 125.5, 125.4, 125.2, 125.1, 123.6, 123.2, 122.8, 121.6, 119.3, 66.1, 51.0, 50.1, 35.0, 34.14, 31.7, 29.7.

HRMS (ESI) *m/z*: [M + H]^+^ Calcd for C_50_H_53_O_2_N_2_ 713.4102, Found:713.4101.

**4-(butoxymethyl)-2-(tert-butyl)-6-((((1S,2S)-2-((3,5-di-tert-butyl-2-hydroxybenzyl)amino)-1,2-diphenylethyl)amino)methyl)phenol (L26)**

A tetrahydrofuran solution of intermediate **I27** was added to an anhydrous ethanol solution of **M3** under a nitrogen atmosphere. The reaction mixture was heated under reflux, with progress monitored by TLC. Upon completion, the mixture was cooled to room temperature and concentrated under reduced pressure. The residue was dissolved in anhydrous methanol and cooled to 0 °C. After purging with nitrogen, NaBH₄ was added portionwise, and the mixture was stirred at 0 °C for 30 min. The reaction was warmed to room temperature (monitored by TLC). After completion, the reaction was quenched with deionized water and extracted with dichloromethane (3 × 100 mL). The combined organic layers were washed with brine, dried over anhydrous sodium sulfate (Na₂SO₄), filtered, and concentrated. The crude product was purified by silica gel chromatography (PE/EA = 5:1) to afford **L26.** (1.61 g, 2.38 mmol, 95% yield); [α]_D_^25^ = +67.0 (c 0.10, CHCl_3_); m.p. 67-69 °C; ^1^H NMR (500 MHz, CDCl_3_) δ 7.22 (td, *J* = 4.5, 2.0 Hz, 7H), 7.14 (d, *J* = 2.1 Hz, 1H), 7.02 – 6.87 (m, 4H), 6.64 (dd, *J* = 6.5, 2.3 Hz, 2H), 4.31 (s, 2H), 4.02 (s, 2H), 3.81 (dd, *J* = 13.4, 3.6 Hz, 2H), 3.60 (dd, *J* = 13.4, 3.7 Hz, 2H), 3.41 (t, *J* = 6.6 Hz, 2H), 1.56 (dq, *J* = 8.6, 6.6 Hz, 2H), 1.43 (d, *J* = 7.1 Hz, 18H), 1.35 (dd, *J* = 8.7, 6.3 Hz, 2H), 1.22 (s, 9H), 0.89 (t, *J* = 7.4 Hz, 3H).

^13^C{^1^H} NMR (101 MHz, CDCl_3_) δ 156.5, 154.4, 140.7, 137.6, 137.56, 136.8, 136.1, 128.6, 128.5, 128.3, 128.2, 128.1, 128.0, 126.7, 126.4, 123.6, 123.2, 122.5, 121.7, 73.2, 70.1, 66.5, 66.4, 51.2, 50.8, 35.1, 34.8, 34.2, 31.9, 31.7, 29.8, 29.6, 19.5, 14.1.

HRMS (ESI) *m/z*: [M + H]^+^ Calcd for C_45_H_63_O_3_N_2_ 679.4833, Found:679.4835.

**2,4-di-tert-butyl-6-((((1S,2S)-2-((2-hydroxy-3-(2-phenylpropan-2-yl)benzyl)amino)-1,2-diphenylethyl)amino)methyl)phenol (L27)**

A tetrahydrofuran solution of intermediate **I28** was added to an anhydrous ethanol solution of **M3** under a nitrogen atmosphere. The reaction mixture was heated under reflux, with progress monitored by TLC. Upon completion, the mixture was cooled to room temperature and concentrated under reduced pressure. The residue was dissolved in anhydrous methanol and cooled to 0 °C. After purging with nitrogen, NaBH₄ was added portionwise, and the mixture was stirred at 0 °C for 30 min. The reaction was warmed to room temperature (monitored by TLC). After completion, the reaction was quenched with deionized water and extracted with dichloromethane (3 × 100 mL). The combined organic layers were washed with brine, dried over anhydrous sodium sulfate (Na₂SO₄), filtered, and concentrated. The crude product was purified by silica gel chromatography (PE/EA = 5:1) to afford **L27.** (1.52 g, 2.33 mmol, 93% yield); [α]_D_^25^ = +93.0 (c 0.10, CHCl_3_); m.p. 93-95 °C; ^1^H NMR (400 MHz, CDCl_3_) δ 7.23 (dt, *J* = 6.9, 1.9 Hz, 5H), 7.21 – 7.16 (m, 6H), 7.15 – 7.11 (m, 1H), 7.05 (dd, *J* = 8.4, 2.4 Hz, 1H), 6.95 – 6.91 (m, 2H), 6.90 – 6.85 (m, 2H), 6.76 (d, *J* = 8.4 Hz, 1H), 6.63 (d, *J* = 2.4 Hz, 1H), 6.59 (d, *J* = 2.4 Hz, 1H), 3.99 (q, *J* = 7.0 Hz, 2H), 3.78 (dd, *J* = 13.6, 2.7 Hz, 2H), 3.60 (d, *J* = 13.2 Hz, 1H), 3.50 (d, *J* = 13.6 Hz, 1H), 1.58 (d, *J* = 3.4 Hz, 6H), 1.44 (s, 9H), 1.23 (s, 9H).

^13^C{^1^H} NMR (101 MHz, CDCl_3_) δ 155.6, 154.3, 151.1, 141.5, 140.8, 137.54, 137.46, 136.1, 128.52, 128.47, 128.3, 128.2, 128.02, 127.98, 127.3, 127.1, 126.8, 125.5, 123.6, 123.3, 121.74, 121.70, 115.8, 66.6, 66.2, 51.1, 50.6, 42.2, 35.0, 34.2, 31.7, 31.0, 30.9, 29.7.

HRMS (ESI) *m/z*: [M + H]^+^ Calcd for C_45_H_55_O_2_N_2_ 655.4258, Found: 655.4260.

**2,4-di-tert-butyl-6-((((1S,2S)-2-((5-(tert-butyl)-2-hydroxy-3-tritylbenzyl)amino)-1,2-diphenylethyl)amino)methyl)phenol (L28)**

A tetrahydrofuran solution of intermediate **I29** was added to an anhydrous ethanol solution of **M3** under a nitrogen atmosphere. The reaction mixture was heated under reflux, with progress monitored by TLC. Upon completion, the mixture was cooled to room temperature and concentrated under reduced pressure. The residue was dissolved in anhydrous methanol and cooled to 0 °C. After purging with nitrogen, NaBH₄ was added portionwise, and the mixture was stirred at 0 °C for 30 min. The reaction was warmed to room temperature (monitored by TLC). After completion, the reaction was quenched with deionized water and extracted with dichloromethane (3 × 100 mL). The combined organic layers were washed with brine, dried over anhydrous sodium sulfate (Na₂SO₄), filtered, and concentrated. The crude product was purified by silica gel chromatography (PE/EA = 5:1) to afford **L28.** (1.96 g, 2.35 mmol, 94% yield); [α]_D_^25^ = +95.0 (c 0.10, CHCl_3_); m.p. 112-115 °C; ^1^H NMR (500 MHz, CDCl_3_) δ 10.60 (s, 1H), 9.85 (s, 1H), 7.31 – 7.26 (m, 5H), 7.22 – 7.11 (m, 18H), 6.72 (dd, *J* = 7.0, 2.8 Hz, 2H), 6.63 (dt, *J* = 8.1, 2.4 Hz, 3H), 6.59 (d, *J* = 2.4 Hz, 1H), 3.93 (d, *J* = 13.3 Hz, 1H), 3.70 (dd, *J* = 10.9, 7.8 Hz, 1H), 3.57 (dd, *J* = 13.6, 4.1 Hz, 1H), 3.49 (dq, *J* = 13.6, 8.1 Hz, 2H), 3.38 (d, *J* = 13.4 Hz, 1H), 1.39 (s, 9H), 1.23 (s, 9H), 1.10 (d, *J* = 1.7 Hz, 9H).

^13^C{^1^H} NMR (126 MHz, CDCl_3_) δ 154.6, 153.8, 146.9, 146.1, 140.5, 140.4, 137.7, 137.5, 135.95, 133.6, 131.3, 128.3, 128.2, 128.17, 128.0, 127.8, 127.7, 127.6, 127.3, 127.0, 125.4, 125.0, 123.5, 122.9, 121.8, 121.7, 65.8, 64.5, 63.5, 51.1, 49.7, 35.0, 34.2, 34.1, 31.7, 31.5, 29.7.

HRMS (ESI) *m/z*: [M + H]^+^ Calcd for C_59_H_67_O_2_N_2_ 835.5197, Found:835.5199.

**2,4-di-tert-butyl-6-((((1S,2S)-2-((2-hydroxy-3,5-bis(2-phenylpropan-2-yl)benzyl)amino)cyclohexyl)amino)methyl)phenol (L29)**

A tetrahydrofuran solution of intermediate **I26** was added to an anhydrous ethanol solution of **M4** under a nitrogen atmosphere. The reaction mixture was heated under reflux, with progress monitored by TLC. Upon completion, the mixture was cooled to room temperature and concentrated under reduced pressure. The residue was dissolved in anhydrous methanol and cooled to 0 °C. After purging with nitrogen, NaBH₄ was added portionwise, and the mixture was stirred at 0 °C for 30 min. The reaction was warmed to room temperature (monitored by TLC). After completion, the reaction was quenched with deionized water and extracted with dichloromethane (3 × 100 mL). The combined organic layers were washed with brine, dried over anhydrous sodium sulfate (Na₂SO₄), filtered, and concentrated. The crude product was purified by silica gel chromatography (PE/EA = 5:1) to afford **L29.** (1.65 g, 2.45 mmol, 98% yield); [α]_D_^25^ = +35.0 (c 0.10, CHCl_3_); m.p. 69-71 °C; ^1^H NMR (400 MHz, CDCl_3_) δ 7.26 (d, *J* = 3.0 Hz, 2H), 7.22 (dd, *J* = 4.2, 2.2 Hz, 3H), 7.18 – 6.98 (m, 7H), 6.81 (d, *J* = 2.4 Hz, 1H), 6.72 (d, *J* = 2.3 Hz, 1H), 3.95 (d, *J* = 13.6 Hz, 1H), 3.86 (d, *J* = 13.5 Hz, 1H), 3.79 (d, *J* = 13.6 Hz, 1H), 3.71 (d, *J* = 13.4 Hz, 1H), 2.22 (td, *J* = 9.6, 3.8 Hz, 1H), 2.15 – 2.03 (m, 2H), 1.96 (dd, *J* = 12.9, 3.8 Hz, 1H), 1.68 (s, 6H), 1.64 (s, 3H), 1.58 (s, 3H), 1.40 (s, 9H), 1.30 (s, 9H), 1.27 – 0.69 (m, 6H).

^13^C{^1^H} NMR (101 MHz, CDCl_3_) δ 154.6, 153.8, 151.5, 151.49, 140.4, 140.0, 135.9, 135.5, 127.9, 127.6, 126.8, 125.75, 125.6, 125.4, 124.8, 124.7, 123.2, 122.9, 122.5, 122.4, 59.8, 59.2, 50.9, 49.8, 42.5, 41.98, 34.9, 34.2, 31.8, 31.7, 31.2, 31.1, 30.9, 30.4, 29.9, 29.7, 29.68, 28.9, 24.3.

HRMS (ESI) *m/z*: [M + H]^+^ Calcd for C_50_H_53_O_2_N_2_ 675.4884, Found:675.4885.

**2,4-di-tert-butyl-6-((((1S,2S)-2-((3-(tert-butyl)-2-hydroxybenzyl)amino)cyclohexyl)amino)methyl)phenol (L30)**

A tetrahydrofuran solution of intermediate **I1** was added to an anhydrous ethanol solution of **M4** under a nitrogen atmosphere. The reaction mixture was heated under reflux, with progress monitored by TLC. Upon completion, the mixture was cooled to room temperature and concentrated under reduced pressure. The residue was dissolved in anhydrous methanol and cooled to 0 °C. After purging with nitrogen, NaBH₄ was added portionwise, and the mixture was stirred at 0 °C for 30 min. The reaction was warmed to room temperature (monitored by TLC). After completion, the reaction was quenched with deionized water and extracted with dichloromethane (3 × 100 mL). The combined organic layers were washed with brine, dried over anhydrous sodium sulfate (Na₂SO₄), filtered, and concentrated. The crude product was purified by silica gel chromatography (PE/EA = 5:1) to afford **L30.** (1.18 g, 2.38 mmol, 95% yield); [α]_D_^25^ = +10.0 (c 0.10, CHCl_3_); m.p. 79-81 °C; ^1^H NMR (400 MHz, CDCl_3_) δ 7.23 – 7.15 (m, 2H), 6.85 (dd, *J* = 7.9, 1.9 Hz, 2H), 6.71 (t, *J* = 7.6 Hz, 1H), 4.05 (dd, *J* = 13.5, 4.4 Hz, 2H), 3.92 (dd, *J* = 16.2, 13.4 Hz, 2H), 2.45 (p, *J* = 3.5 Hz, 2H), 2.17 (t, *J* = 14.0 Hz, 2H), 1.74 – 1.67 (m, 2H), 1.37 (d, *J* = 4.5 Hz, 18H), 1.28 (s, 9H), 1.27 – 1.13 (m, 4H).

^13^C{^1^H} NMR (101 MHz, CDCl_3_) δ 157.0, 154.4, 140.7, 136.9, 136.0, 126.5, 126.0, 123.2, 123.2, 123.1, 122.3, 118.4, 77.2, 59.9, 50.8, 50.5, 34.9, 34.7, 34.2, 31.7, 30.8, 30.75, 29.6, 29.5, 24.2.

HRMS (ESI) *m/z*: [M + H]^+^ Calcd for C_50_H_53_O_2_N_2_ 495.3945, Found:495.3950.

**2-((3S,5S,7S)-adamantan-1-yl)-4-(tert-butyl)-6-((((1S,2S)-2-((3,5-di-tert-butyl-2-hydroxybenzyl)amino)cyclohexyl)amino)methyl)phenol (L31)**

A tetrahydrofuran solution of intermediate **I31** was added to an anhydrous ethanol solution of **M4** under a nitrogen atmosphere. The reaction mixture was heated under reflux, with progress monitored by TLC. Upon completion, the mixture was cooled to room temperature and concentrated under reduced pressure. The residue was dissolved in anhydrous methanol and cooled to 0 °C. After purging with nitrogen, NaBH₄ was added portionwise, and the mixture was stirred at 0 °C for 30 min. The reaction was warmed to room temperature (monitored by TLC). After completion, the reaction was quenched with deionized water and extracted with dichloromethane (3 × 100 mL). The combined organic layers were washed with brine, dried over anhydrous sodium sulfate (Na₂SO₄), filtered, and concentrated. The crude product was purified by silica gel chromatography (PE/EA = 5:1) to afford **L31.** (1.44 g, 2.33 mmol, 93% yield); [α]_D_^25^ = +23.0 (c 0.10, CHCl_3_); m.p. 88-90 °C; ^1^H NMR (400 MHz, CDCl_3_) δ 7.21 (d, *J* = 2.4 Hz, 1H), 7.15 (d, *J* = 2.7 Hz, 1H), 6.85 (t, *J* = 2.8 Hz, 2H), 4.04 (d, *J* = 13.3 Hz, 2H), 3.90 (dd, *J* = 13.3, 4.0 Hz, 2H), 2.47 (dq, *J* = 6.2, 3.2 Hz, 2H), 2.21 – 2.14 (m, 3H), 2.12 (d, *J* = 2.9 Hz, 5H), 2.04 – 1.94 (m, 3H), 1.75 – 1.69 (m, 6H), 1.39 – 1.36 (m, 9H), 1.34 – 1.30 (m, 2H), 1.29 – 1.27 (m, 18H), 1.26 – 1.22 (m, 4H).

^13^C{^1^H} NMR (101 MHz, CDCl_3_) δ 154.6, 154.4, 140.9, 140.6, 136.4, 136.0, 123.1, 123.06, 122.97, 122.5, 122.3, 59.9, 59.8, 51.0, 50.8, 41.3, 40.5, 37.2, 37.1, 37.0, 34.9, 34.2, 34.17, 31.8, 31.7, 31.7, 31.66, 30.8, 30.7, 29.65, 29.2, 29.1, 24.2, 24.1.

HRMS (ESI) *m/z*: [M + H]^+^ Calcd for C_50_H_53_O_2_N_2_ 629.5041, Found:629.5038.

**2,4-di-tert-butyl-6-((((1S,2S)-2-((5-(tert-butyl)-2-hydroxy-3-tritylbenzyl)amino)cyclohexyl)amino)methyl)phenol (L32)**

A tetrahydrofuran solution of intermediate **I29** was added to an anhydrous ethanol solution of **M4** under a nitrogen atmosphere. The reaction mixture was heated under reflux, with progress monitored by TLC. Upon completion, the mixture was cooled to room temperature and concentrated under reduced pressure. The residue was dissolved in anhydrous methanol and cooled to 0 °C. After purging with nitrogen, NaBH₄ was added portionwise, and the mixture was stirred at 0 °C for 30 min. The reaction was warmed to room temperature (monitored by TLC). After completion, the reaction was quenched with deionized water and extracted with dichloromethane (3 × 100 mL). The combined organic layers were washed with brine, dried over anhydrous sodium sulfate (Na₂SO₄), filtered, and concentrated. The crude product was purified by silica gel chromatography (PE/EA = 5:1) to afford **L32.** (1.68 g, 2.28 mmol, 91% yield); [α]_D_^25^ = +5.0 (c 0.10, CHCl_3_); m.p. 93-95 °C; ^1^H NMR (400 MHz, CDCl_3_) δ 7.21 – 7.16 (m, 8H), 7.14 (s, 3H), 7.13 – 7.07 (m, 5H), 6.93 (d, *J* = 2.4 Hz, 1H), 6.79 (d, *J* = 2.4 Hz, 1H), 3.98 (q, *J* = 13.7 Hz, 2H), 3.83 (d, *J* = 13.5 Hz, 1H), 3.71 (d, *J* = 13.5 Hz, 1H), 2.16 (td, *J* = 9.6, 3.8 Hz, 1H), 2.02 (tt, *J* = 9.5, 4.1 Hz, 2H), 1.85 (dd, *J* = 13.5, 4.0 Hz, 1H), 1.62 (tt, *J* = 9.4, 2.1 Hz, 2H), 1.57 – 1.52 (m, 1H), 1.36 (s, 9H), 1.28 (s, 9H), 1.14 (s, 9H), 1.11 – 1.00 (m, 3H).

^13^C{^1^H} NMR (101 MHz, CDCl_3_) δ 154.6, 153.8, 146.1, 140.4, 140.4, 135.9, 133.5, 131.2, 127.6, 127.0, 125.4, 124.2, 123.1, 122.7, 122.66, 122.3, 63.4, 59.9, 59.2, 51.1, 49.9, 34.9, 34.2, 34.1, 31.8, 31.6, 30.9, 30.3, 29.7, 26.97, 24.3, 24.2.

HRMS (ESI) *m/z*: [M + H]^+^ Calcd for C_50_H_53_O_2_N_2_ 737.5041, Found:737.5039.

**3-(tert-butyl)-5-((((1S,2S)-2-((3,5-di-tert-butyl-2-hydroxybenzyl)amino)cyclohexyl)amino)methyl)-4-hydroxyphenyl pivalate (L33)**

A tetrahydrofuran solution of intermediate **I30** was added to an anhydrous ethanol solution of **M4** under a nitrogen atmosphere. The reaction mixture was heated under reflux, with progress monitored by TLC. Upon completion, the mixture was cooled to room temperature and concentrated under reduced pressure. The residue was dissolved in anhydrous methanol and cooled to 0 °C. After purging with nitrogen, NaBH₄ was added portionwise, and the mixture was stirred at 0 °C for 30 min. The reaction was warmed to room temperature (monitored by TLC). After completion, the reaction was quenched with deionized water and extracted with dichloromethane (3 × 100 mL). The combined organic layers were washed with brine, dried over anhydrous sodium sulfate (Na₂SO₄), filtered, and concentrated. The crude product was purified by silica gel chromatography (PE/EA = 5:1) to afford **L33.** (1.35 g, 2.27 mmol, 91% yield); [α]_D_^25^ = +13.0 (c 0.10, CHCl_3_); m.p. 63-65 °C; ^1^H NMR (400 MHz, CDCl_3_) δ 7.22 (dd, *J* = 2.5, 1.1 Hz, 1H), 6.87 (dd, *J* = 2.4, 1.1 Hz, 1H), 6.81 (dd, *J* = 2.9, 1.2 Hz, 1H), 6.64 – 6.57 (m, 1H), 4.06 (d, *J* = 13.4 Hz, 1H), 3.99 (d, *J* = 13.7 Hz, 1H), 3.91 (dd, *J* = 13.5, 6.5 Hz, 2H), 2.43 (h, *J* = 4.9 Hz, 2H), 2.29 – 2.04 (m, 2H), 1.82 – 1.61 (m, 2H), 1.38 (d, *J* = 1.2 Hz, 9H), 1.34 (dd, *J* = 3.3, 1.1 Hz, 18H), 1.29 (d, *J* = 1.2 Hz, 9H), 1.27 – 1.17 (m, 4H).

^13^C{^1^H} NMR (101 MHz, CDCl_3_) δ 177.7, 154.5, 154.4, 142.5, 140.7, 137.9, 136.0, 123.4, 123.2, 123.1, 122.3, 118.9, 118.8, 60.0, 59.8, 50.7, 50.4, 39.0, 34.9, 34.8, 34.2, 31.7, 30.8, 29.7, 29.3, 27.3, 27.2, 24.2.

HRMS (ESI) *m/z*: [M + H]^+^ Calcd for C_37_H_59_O_4_N_2_ 595.4469, Found:595.4461.

**2,4-di-tert-butyl-6-((((1S,2S)-2-((3-(tert-butyl)-5-((diphenylamino)methyl)-2-hydroxybenzyl)amino)cyclohexyl)amino)methyl)phenol (L34)**

A tetrahydrofuran solution of intermediate **I8** was added to an anhydrous ethanol solution of **M4** under a nitrogen atmosphere. The reaction mixture was heated under reflux, with progress monitored by TLC. Upon completion, the mixture was cooled to room temperature and concentrated under reduced pressure. The residue was dissolved in anhydrous methanol and cooled to 0 °C. After purging with nitrogen, NaBH₄ was added portionwise, and the mixture was stirred at 0 °C for 30 min. The reaction was warmed to room temperature (monitored by TLC). After completion, the reaction was quenched with deionized water and extracted with dichloromethane (3 × 100 mL). The combined organic layers were washed with brine, dried over anhydrous sodium sulfate (Na₂SO₄), filtered, and concentrated. The crude product was purified by silica gel chromatography (PE/EA = 5:1) to afford **L34.** (1.52 g, 2.25 mmol, 90% yield); [α]_D_^25^ = +23.0 (c 0.10, CHCl_3_); m.p. 69-71 °C; ^1^H NMR (400 MHz, CDCl_3_) δ 7.18 – 7.11 (m, 5H), 7.03 – 6.95 (m, 5H), 6.83 (tt, *J* = 7.2, 1.2 Hz, 2H), 6.78 (d, *J* = 2.4 Hz, 1H), 6.73 (d, *J* = 2.1 Hz, 1H), 4.80 (s, 2H), 3.99 – 3.87 (m, 2H), 3.79 (dd, *J* = 13.5, 9.1 Hz, 2H), 2.32 (dqd, *J* = 12.3, 8.8, 3.6 Hz, 2H), 2.18 – 1.98 (m, 2H), 1.67 – 1.57 (m, 2H), 1.30 (s, 9H), 1.22 (s, 9H), 1.20 (s, 9H), 1.17 – 1.05 (m, 4H).

^13^C{^1^H} NMR (101 MHz, CDCl_3_) δ 154.5, 153.3, 147.2, 139.5, 135.8, 134.9, 128.1, 127.2, 123.5, 123.4, 122.1, 122.06, 121.97, 121.3, 120.1, 119.7, 58.8, 54.9, 49.7, 49.5, 33.8, 33.6, 33.1, 30.7, 29.7, 29.6, 28.7, 28.6, 28.4, 23.1.

HRMS (ESI) *m/z*: [M + H]^+^ Calcd for C_45_H_62_O_2_N_3_ 676.4837, Found:676.4842.

**6,6'-((((1S,2S)-cyclohexane-1,2-diyl)bis(azanediyl))bis(methylene))bis(2,4-bis(2-phenylpropan-2-yl)phenol) (L35)**

A tetrahydrofuran solution of intermediate **I26** was added to an anhydrous ethanol solution of (1S,2S)-(+)-1,2-Diaminocyclohexane under a nitrogen atmosphere. The reaction mixture was heated under reflux, with progress monitored by TLC. Upon completion, the mixture was cooled to room temperature and concentrated under reduced pressure. The residue was dissolved in anhydrous methanol and cooled to 0 °C. After purging with nitrogen, NaBH₄ was added portionwise, and the mixture was stirred at 0 °C for 30 min. The reaction was warmed to room temperature (monitored by TLC). After completion, the reaction was quenched with deionized water and extracted with dichloromethane (3 × 100 mL). The combined organic layers were washed with brine, dried over anhydrous sodium sulfate (Na₂SO₄), filtered, and concentrated. The crude product was purified by silica gel chromatography (PE/EA = 5:1) to afford **L35.** (1.92 g, 2.40 mmol, 96% yield); [α]_D_^25^ = +43.0 (c 0.10, CHCl_3_); m.p. 77-79 °C; ^1^H NMR (400 MHz, CDCl_3_) δ 7.31 – 7.23 (m, 10H), 7.19 – 7.07 (m, 10H), 7.01 (dq, *J* = 6.6, 2.8 Hz, 2H), 6.63 (d, *J* = 2.3 Hz, 2H), 3.71 (d, *J* = 13.7 Hz, 2H), 3.54 (d, *J* = 13.8 Hz, 2H), 1.82 (d, *J* = 12.9 Hz, 2H), 1.69 (d, *J* = 6.1 Hz, 18H), 1.65 (d, *J* = 9.3 Hz, 2H), 1.56 (s, 6H), 1.51 (s, 2H), 0.93 – 0.85 (m, 2H), 0.66 – 0.56 (m, 2H).

^13^C{^1^H} NMR (101 MHz, CDCl_3_) δ 153.9, 152.0, 151.6, 139.7, 135.3, 127.9, 127.5, 126.8, 125.8, 125.6, 125.4, 124.5, 124.2, 122.0, 57.9, 49.2, 42.5, 41.9, 31.2, 31.1, 30.8, 30.0, 27.9, 24.5.

HRMS (ESI) *m/z*: [M + H]^+^ Calcd for C_50_H_53_O_2_N_2_ 799.5197, Found:799.5199.

**6,6'-((((1S,2S)-cyclohexane-1,2-diyl)bis(azanediyl))bis(methylene))bis(2-(tert-butyl)-4-methoxyphenol) (L36)**

A tetrahydrofuran solution of intermediate **I2** was added to an anhydrous ethanol solution of (1S,2S)-(+)-1,2-Diaminocyclohexane under a nitrogen atmosphere. The reaction mixture was heated under reflux, with progress monitored by TLC. Upon completion, the mixture was cooled to room temperature and concentrated under reduced pressure. The residue was dissolved in anhydrous methanol and cooled to 0 °C. After purging with nitrogen, NaBH₄ was added portionwise, and the mixture was stirred at 0 °C for 30 min. The reaction was warmed to room temperature (monitored by TLC). After completion, the reaction was quenched with deionized water and extracted with dichloromethane (3 × 100 mL). The combined organic layers were washed with brine, dried over anhydrous sodium sulfate (Na₂SO₄), filtered, and concentrated. The crude product was purified by silica gel chromatography (PE/EA = 5:1) to afford **L36.** (1.15 g, 2.30 mmol, 92% yield); [α]_D_^25^ = +40.0 (c 0.10, CHCl_3_); m.p. 87-89 °C; ^1^H NMR (400 MHz, CDCl_3_) δ 6.79 (d, *J* = 3.0 Hz, 2H), 6.43 (d, *J* = 3.0 Hz, 2H), 4.02 (d, *J* = 13.5 Hz, 2H), 3.89 (d, *J* = 13.5 Hz, 2H), 3.74 (s, 6H), 2.44 – 2.34 (m, 2H), 2.23 – 2.10 (m, 2H), 1.77 – 1.65 (m, 2H), 1.36 (s, 16H), 1.27 – 1.09 (m, 4H).

^13^C{^1^H} NMR (101 MHz, CDCl_3_) δ 151.7, 150.7, 138.2, 123.5, 112.8, 111.0, 59.9, 55.8, 50.6, 34.8, 31.0, 29.4, 24.3.

HRMS (ESI) *m/z*: [M + H]^+^ Calcd for C_30_H_47_O_4_N_2_ 499.3530, Found:499.3535.

**2-((((1S,2S)-2-((3-(tert-butyl)-2-hydroxybenzyl)amino)cyclohexyl)amino)methyl)-4,6-bis(2-phenylpropan-2-yl)phenol (L37)**

A tetrahydrofuran solution of intermediate **I26** was added to an anhydrous ethanol solution of **M5** under a nitrogen atmosphere. The reaction mixture was heated under reflux, with progress monitored by TLC. Upon completion, the mixture was cooled to room temperature and concentrated under reduced pressure. The residue was dissolved in anhydrous methanol and cooled to 0 °C. After purging with nitrogen, NaBH₄ was added portionwise, and the mixture was stirred at 0 °C for 30 min. The reaction was warmed to room temperature (monitored by TLC). After completion, the reaction was quenched with deionized water and extracted with dichloromethane (3 × 100 mL). The combined organic layers were washed with brine, dried over anhydrous sodium sulfate (Na₂SO₄), filtered, and concentrated. The crude product was purified by silica gel chromatography (PE/EA = 5:1) to afford **L37.** (1.42 g, 2.30 mmol, 92% yield); [α]_D_^25^ = +45.0 (c 0.10, CHCl_3_); m.p. 57-60 °C; ^1^H NMR (400 MHz, CDCl_3_) δ 7.30 – 7.23 (m, *J* = 2.1 Hz, 5H), 7.20 – 7.14 (m, 2H), 7.13 – 7.10 (m, 3H), 7.05 (ddt, *J* = 6.6, 5.0, 2.4 Hz, 1H), 6.78 (dt, *J* = 7.5, 2.1 Hz, 1H), 6.70 (dt, *J* = 9.9, 3.8 Hz, 2H), 3.97 (dd, *J* = 13.6, 1.9 Hz, 1H), 3.86 – 3.69 (m, 3H), 2.17 (td, *J* = 9.9, 3.6 Hz, 1H), 2.10 – 1.92 (m, 3H), 1.68 (d, *J* = 2.2 Hz, 6H), 1.65 (d, *J* = 2.4 Hz, 3H), 1.57 (d, *J* = 2.3 Hz, 3H), 1.42 – 1.37 (m, 9H), 1.15 – 1.04 (m, 2H), 0.96 – 0.81 (m, 2H).

^13^C{^1^H} NMR (101 MHz, CDCl_3_) δ 157.2, 153.8, 151.5, 151.51, 140.1, 136.8, 135.5, 127.9, 127.7, 126.8, 126.5, 125.9, 125.8, 125.6, 125.4, 124.8, 124.7, 123.2, 122.4, 118.2, 59.8, 59.1, 50.6, 49.7, 42.5, 42.0, 34.7, 31.2, 31.1, 31.0 , 30.6, 30.0, 29.6, 28.8, 24.3.

HRMS (ESI) *m/z*: [M + H]^+^ Calcd for C_42_H_55_O_2_N_2_ 619.4258, Found:619.4263.

## General procedure (A) for the reaction of α-hydroxylation of β-ketoester compounds 1a-1ak

A dried 10mL Schlenk tube equipped with a magnetic stir bar was charged with Zr(acac)_4_ (1.4928 mg, 0.003 mmol), the salan ligand **L16** (2.1876 mg, 0.0033 mmol), and cumene (2 mL). The mixture was stirred at 50°C for 30 min. Then, the β-keto ester **1a-1ag** (0.1 mmol), 80% CHP (37.26 mg, 0.15 mmol) were added. The resulting solution was stirred at 70°C for 20 min (monitored by TLC). Afterwards, the temperature was cooled to room temperature. The crude product solution was concentrated in vacuo, and the residue was purified by column chromatography on silica gel using petroleum ether/ethyl acetate mixtures (5:1, 3:1, or 2:1) to afford the products **1a-2ag**. The enantiomeric excess was determined by chiral HPLC analysis. The absolute configuration was determined by comparing the optical rotation and HPLC retention time of an authentic sample.

## General procedure (B) for the reaction of α-hydroxylation of β-keto amide compounds 1ah-1ak

A dried 10mL Schlenk tube equipped with a magnetic stir bar was charged with Zr(acac)4 (2.881 mg, 0.01 mmol), the salan ligand **L16** (3.6460 mg, 0.011 mmol), and cumene (2 mL). The mixture was stirred at 50°C for 30 min. β-Keto ester 3ac-3ag (0.1 mmol), 80% CHP (37.26 mg, 0.15 mmol), were added. The resulting solution was stirred at 50 °C for 60 min (monitored by TLC). Afterwards, the temperature was cooled to room temperature. The crude product solution was concentrated in vacuo, and the residue was purified by column chromatography on silica gel using petroleum ether/ethyl acetate mixtures (5:1, 3:1, or 2:1) to afford the products **2ah-2ak**. The enantiomeric excess was determined by chiral HPLC analysis. The absolute configuration was determined by comparison with the optical rotation and the HPLC retention time of an authentic sample.

## General procedure (C) for the reaction of α-hydroxylation of β-ketoester compounds

A dried 10mL Schlenk tube equipped with a magnetic stir bar was charged with Zr(acac)_4_ (0.9952 mg, 0.002 mmol), the salan ligand **L29** (1.4850 mg, 0.0022 mmol), and ethyl acetate (2 mL). The mixture was stirred at 50°C for 30 min. Then, the β-keto ester (0.1 mmol), 80% CHP (37.26 mg, 0.15 mmol) were added. The resulting solution was stirred at 50°C for 180 min (monitored by TLC). Afterwards, the temperature was cooled to room temperature. The solution of the crude product was concentrated in vacuo, and the residue was purified by column chromatography on silica gel (petroleum ether/ethyl acetate = 5/1, 3/1, or 2/1) to afford the products. The enantiomeric excess was determined by chiral HPLC analysis. The absolute configuration was determined by comparison with the optical rotation and the HPLC retention time of an authentic sample.

## General procedure (D) for the reaction of α-hydroxylation of β-ketoester compounds 1al-1ar

A dried 10mL Schlenk tube equipped with a magnetic stir bar was charged with Zr(acac)_4_ (2.4881 mg, 0.005 mmol), the salan ligand **L29** (3.7126 mg, 0.0055 mmol), and cumene (2 mL). The mixture was stirred at 50°C for 30 min. Then, cool down to 15℃. β-Keto ester **1al-1ar** (0.1 mmol), 80% CHP (37.26 mg, 0.15 mmol), were added. The resulting solution was stirred at 15°C for 24 h (monitored by TLC). Afterwards, the temperature was cooled to room temperature. The crude product solution was concentrated in vacuo, and the residue was purified by column chromatography on silica gel using petroleum ether/ethyl acetate mixtures (5:1, 3:1, or 2:1) to afford the products **2al-2ar**. The enantiomeric excess was determined by chiral HPLC analysis. The absolute configuration was determined by comparison with the optical rotation and the HPLC retention time of an authentic sample.

**(S)-methyl 5-chloro-2-hydroxy-1-oxo-2,3-dihydro-1H-indene-2-carboxylate (2a)^2c, 3-4^**

Prepared according to the general procedure with a reaction time of 1 h. After [column](app:ds:column) [chromatography](app:ds:chromatography) (silica gel, petroleum ether/ethyl acetate = 5/1 ) to give **2a** as white solid; 23.8 mg, 99% yield, 99% ee; [α]_D_^25^ = +109.7 (c 0.15, CHCl_3_); ^1^H NMR (500 MHz, CDCl_3_) δ 7.73 (d, *J* = 8.2 Hz, 1H), 7.50 (s, 1H), 7.41 (d, *J* = 8.2 Hz, 1H), 4.19 (s, 1H), 3.75 (s, 3H), 3.71 (d, *J* = 17.5 Hz, 1H), 3.24 (d, *J* = 17.4 Hz, 1H).

^13^C{^1^H} NMR (126 MHz, CDCl_3_) δ 199.5, 171.5, 153.6, 142.8, 132.0, 129.0, 126.8, 126.4, 80.4, 53.6, 39.0.

HPLC conditions: Chiralcel OD-H column (250×4.6 mm), hexane/*i*-PrOH = 98/2, 1 mL/min, 254 nm, τ_R_ (major) = 19.645, τ_R_ (minor) = 25.392 min.

**(S)-****Methyl 5-bromo-2-hydroxy-1-oxo-2,3-dihydro-1H-indene-2-carboxylate (2b)^2c, 3^**

Prepared according to the general procedure with a reaction time of 1 h. After [column](app:ds:column) [chromatography](app:ds:chromatography) (silica gel, petroleum ether/ethyl acetate = 5/1 ) to give **2b** as white solid; 28.2 mg, 99% yield, 99% ee; [α]_D_^25^ = +83.4 (c 0.20, CHCl_3_); ^1^H NMR (400 MHz, CDCl_3_) δ 7.73 – 7.63 (m, 2H), 7.63 – 7.53 (m, 1H), 3.97 (s, 1H), 3.75 (s, 3H), 3.70 (d, *J* = 17.5 Hz, 1H), 3.24 (d, *J* = 17.5 Hz, 1H).

^13^C{^1^H} NMR (101 MHz, CDCl_3_) δ 199.6, 171.5, 153.6, 132.4, 131.9, 131.8, 129.9, 126.4, 80.3, 53.6, 38.9.

HPLC conditions: Chiralcel OD-H column (250×4.6 mm), hexane/*i*-PrOH = 90/10, 1 mL/min, 254 nm, τ_R_ (major) = 22.277 min, τ_R_ (minor) = 27.732 min.

**(S)-Methyl 5-fluoro-2-hydroxy-1-oxo-2,3-dihydro-1H-indene-2-carboxylate (2c)^2c, 3^**

Prepared according to the general procedure with a reaction time of 1 h. After [column](app:ds:column) [chromatography](app:ds:chromatography) (silica gel, petroleum ether/ethyl acetate = 5/1 ) to give **2c** as white solid; 22.0 mg, 98% yield, 98% ee; [α]_D_^25^ = +72.0 (c 0.30, CHCl_3_); ^1^H NMR (400 MHz, CDCl_3_) δ 7.82 (dd, *J* = 8.4, 5.3 Hz, 1H), 7.23 – 7.07 (m, 2H), 4.05 (s, 1H), 3.75 (s, 3H), 3.74 (s, 1H), 3.25 (dd, *J* = 17.5, 1.2 Hz, 1H).

^13^C{^1^H} NMR (101 MHz, CDCl_3_) δ 198.9, 171.6, 169.2, 166.7, 155.2, 155.1, 129.9, 129.9, 127.9, 127.7, 116.8, 116.5, 113.5, 113.3, 80.5, 53.6, 39.2, 39.1.

HPLC conditions: Chiralcel OD-H column (250×4.6 mm), hexane/*i*-PrOH = 90/10, 1 mL/min, 254 nm, τ_R_ (major) = 20.200 min, τ_R_ (minor) = 26.742 min.

**(S)-Methyl 2-hydroxy-5-methyl-1-oxo-2,3-dihydro-1H-indene-2-carboxylate (2d)^2c, 3^**

Prepared according to the general procedure with a reaction time of 1 h. After [column](app:ds:column) [chromatography](app:ds:chromatography) (silica gel, petroleum ether/ethyl acetate = 5/1 ) to give **2d** as white solid; 21.8 mg, 99% yield, 99% ee; [α]_D_^25^ =+ 213.0 (c 0.10, CHCl_3_); ^1^H NMR (400 MHz, CDCl_3_) δ 7.69 (d, *J* = 7.9 Hz, 1H), 7.33 – 7.28 (m, 1H), 7.24 (d, *J* = 7.9 Hz, 1H), 4.07 (s, 1H), 3.73 (s, 3H), 3.68 (d, *J* = 17.3 Hz, 1H), 3.20 (d, *J* = 17.2 Hz, 1H), 2.47 (s, 3H).

^13^C{^1^H} NMR (101 MHz, CDCl_3_) δ 200.2, 172.1, 152.7, 147.8, 131.2, 129.5, 126.8, 125.2, 80.6, 53.4, 39.1, 22.3.

HPLC conditions: Chiralcel OD-H column (250×4.6 mm), hexane/*i*-PrOH = 90/10, 1 mL/min, 254 nm, τ_R_ (major) = 18.248 min, τ_R_ (minor) = 23.510 min.

**(S)-Methyl 2-hydroxy-5-methoxy-1-oxo-2,3-dihydro-1H-indene-2-carboxylate (2e)^2c, 3^**

Prepared according to the general procedure with a reaction time of 1 h. After [column](app:ds:column) [chromatography](app:ds:chromatography) (silica gel, petroleum ether/ethyl acetate = 5/1 ) to give **2e** as white solid; 23.1 mg, 98% yield, 98% ee; [α]_D_^25^ = +137.8 (c 0.11, CHCl_3_); ^1^H NMR (400 MHz, CDCl_3_) δ 7.72 (d, *J* = 8.5 Hz, 1H), 7.01 – 6.87 (m, 2H), 4.17 (s, 1H), 3.91 (s, 3H), 3.73 (s, 3H), 3.68 (d, *J* = 17.3 Hz, 1H), 3.20 (d, *J* = 17.3 Hz, 1H).

^13^C{^1^H} NMR (101 MHz, CDCl_3_) δ 198.8, 172.1, 166.5, 155.4, 127.1, 126.6, 116.3, 109.6, 80.7, 55.8, 53.4, 39.3.

HPLC conditions: Chiralcel OD-H column (250×4.6 mm), hexane/*i*-PrOH = 90/10, 1 mL/min, 254 nm, τ_R_ (major) = 19.412 min, τ_R_ (minor) = 24.132 min.

**(S)-Methyl 2-hydroxy-1-oxo-2,3-dihydro-1H-indene-2-carboxylate (2f)^2c, 3^**

Prepared according to the general procedure with a reaction time of 1 h. After [column](app:ds:column) [chromatography](app:ds:chromatography) (silica gel, petroleum ether/ethyl acetate = 5/1 ) to give **2f** as white solid; 20.4 mg, 99% yield, 99% ee; [α]_D_^25^ = +80.6 (c 0.13, CHCl_3_); ^1^H NMR (400 MHz, CDCl_3_) δ 7.83 – 7.74 (m, 1H), 7.67 (td, *J* = 7.5, 1.3 Hz, 1H), 7.49 (dt, *J* = 7.7, 1.0 Hz, 1H), 7.46 – 7.37 (m, 1H), 4.10 (s, 1H), 3.73 (s, 4H), 3.26 (d, *J* = 17.3 Hz, 1H).

^13^C{^1^H} NMR (101 MHz, CDCl_3_) δ 200.9, 171.9, 152.2, 136.2, 133.6, 128.2, 126.5, 125.3, 80.4, 53.5, 39.3.

HPLC conditions: Chiralcel OD-H column (250×4.6 mm), hexane/*i*-PrOH = 90/10, 1 mL/min, 254 nm, τ_R_ (major) = 18.597 min, τ_R_ (minor) = 23.542 min.

**(S)-Methyl 2-hydroxy-5,6-dimethoxy-1-oxo-2,3-dihydro-1H-indene-2-carboxylate (2g)^2c, 3^**

Prepared according to the general procedure with a reaction time of 1 h. After [column](app:ds:column) [chromatography](app:ds:chromatography) (silica gel, petroleum ether/ethyl acetate = 5/1 ) to give **2g** as white solid; 26.4 mg, 99% yield, 99% ee; [α]_D_^25^ = +117.4 (c 0.15, CHCl_3_); ^1^H NMR (400 MHz, CDCl_3_) δ 7.12 (s, 1H), 6.83 (s, 1H), 3.94 (s, 1H), 3.93 (s, 3H), 3.84 (s, 3H), 3.67 (s, 3H), 3.57 (d, *J* = 17.0 Hz, 1H), 3.09 (d, *J* = 17.0 Hz, 1H).

^13^C{^1^H} NMR (101 MHz, CDCl_3_) δ 199.1, 172.2, 156.8, 150.0, 148.1, 126.1, 107.3, 105.4, 80.8, 56.4, 56.2, 53.4, 39.0.

HPLC conditions: Chiralcel AD-H column (250×4.6 mm), hexane/*i*-PrOH = 80/20, 1 mL/min, 254 nm, τ_R_ (major) = 24.602 min, τ_R_ (minor) = 29.650 min.

**(S)-Methyl 4-chloro-2-hydroxy-1-oxo-2,3-dihydro-1H-indene-2-carboxylate (2h)^2c, 3^**

Prepared according to the general procedure with a reaction time of 1 h. After [column](app:ds:column) [chromatography](app:ds:chromatography) (silica gel, petroleum ether/ethyl acetate = 5/1 ) to give **2h** as white solid; 23.6 mg, 98% yield, 98% ee; [α]_D_^25^ = +60.5 (c 0.20, CHCl_3_); ^1^H NMR (400 MHz, CDCl_3_) δ 7.64 (d, *J* = 7.6 Hz, 1H), 7.59 (d, *J* = 7.8 Hz, 1H), 7.34 (t, *J* = 7.7 Hz, 1H), 4.03 (s, 1H), 3.76 – 3.57 (m, 4H), 3.14 (d, *J* = 17.9 Hz, 1H).

^13^C{^1^H} NMR (101 MHz, CDCl_3_) δ 200.1, 171.6, 149.9, 135.8, 135.4, 132.8, 129.7, 123.5, 80.0, 53.7, 38.4.

HPLC conditions: Chiralcel OD-H column (250×4.6 mm), hexane/*i*-PrOH = 98/2, 1 mL/min, 254 nm, τ_R_ (major) = 26.655 min, τ_R_ (minor) = 29.493 min.

**(S)-Methyl 4-bromo-2-hydroxy-1-oxo-2,3-dihydro-1H-indene-2-carboxylate (2i)^2c, 3^**

Prepared according to the general procedure with a reaction time of 1 h. After [column](app:ds:column) [chromatography](app:ds:chromatography) (silica gel, petroleum ether/ethyl acetate = 5/1 ) to give **2i** as white solid; 27.7 mg, 97% yield, 99% ee; [α]_D_^25^ = +63.2 (c 0.15, CHCl_3_); ^1^H NMR (400 MHz, CDCl_3_) δ 7.77 (dd, *J* = 7.8, 1.0 Hz, 1H), 7.69 (dd, *J* = 7.6, 1.0 Hz, 1H), 7.27 (tt, *J* = 7.7, 0.9 Hz, 1H), 3.95 (s, 1H), 3.69 (s, 3H), 3.60 (d, *J* = 17.8 Hz, 1H), 3.11 (d, *J* = 17.8 Hz, 1H).

^13^C{^1^H} NMR (101 MHz, CDCl_3_) δ 200.1, 171.5, 151.9, 138.8, 135.5, 129.9, 124.1, 121.9, 80.1, 53.6, 40.4.

HPLC conditions: Chiralcel OD-H column (250×4.6 mm), hexane/*i*-PrOH = 98/2, 1 mL/min, 254 nm, τ_R_ (major) = 27.980 min, τ_R_ (minor) = 30.888 min.

**(S)-Methyl 2-hydroxy-4-methyl-1-oxo-2,3-dihydro-1H-indene-2-carboxylate (2j)^2c, 3^**

Prepared according to the general procedure with a reaction time of 1 h. After [column](app:ds:column) [chromatography](app:ds:chromatography) (silica gel, petroleum ether/ethyl acetate = 5/1 ) to give **2j** as white solid; 21.8 mg, 99% yield, 99% ee; [α]_D_^25^ = +70.5 (c 0.20, CHCl_3_); ^1^H NMR (400 MHz, CDCl_3_) δ 7.64 (d, *J* = 7.6 Hz, 1H), 7.48 (dt, *J* = 7.4, 1.0 Hz, 1H), 7.35 (t, *J* = 7.5 Hz, 1H), 4.08 (s, 1H), 3.74 (s, 3H), 3.64 (d, *J* = 17.4 Hz, 1H), 3.12 (d, *J* = 17.3 Hz, 1H), 2.36 (s, 3H).

^13^C{^1^H} NMR (101 MHz, CDCl_3_) δ 201.2, 172.1, 151.3, 136.7, 135.8, 133.3, 128.4, 122.7, 80.3, 53.5, 38.2, 17.8.

HPLC conditions: Chiralcel OD-H column (250×4.6 mm), hexane/*i*-PrOH = 90/10, 1 mL/min, 254 nm, τ_R_ (major) = 16.120 min, τ_R_ (minor) = 18.550 min.

**(S)-Methyl 2-hydroxy-4-methoxy-1-oxo-2,3-dihydro-1H-indene-2-carboxylate (2k)^2c, 3^**

Prepared according to the general procedure with a reaction time of 1 h. After [column](app:ds:column) [chromatography](app:ds:chromatography) (silica gel, petroleum ether/ethyl acetate = 5/1 ) to give **2k** as white solid; 23.1 mg, 98% yield, 99% ee; [α]_D_^25^ = +66.0 (c 0.11, CHCl_3_); ^1^H NMR (400 MHz, CDCl_3_) δ 7.49 – 7.35 (m, 2H), 7.11 (dd, *J* = 6.7, 2.1 Hz, 1H), 3.97 (s, 1H), 3.92 (s, 3H), 3.74 (s, 3H), 3.66 (d, *J* = 17.7 Hz, 1H), 3.11 (d, *J* = 17.7 Hz, 1H).

^13^C{^1^H} NMR (101 MHz, CDCl_3_) δ 201.0, 172.1, 156.7, 141.2, 134.9, 129.7, 116.7, 116.3, 80.2, 55.6, 53.5, 36.2.

HPLC conditions: Chiralcel OD-H column (250×4.6 mm), hexane/*i*-PrOH = 90/10, 1 mL/min, 254 nm, τ_R_ (minor) = 25.318 min, τ_R_ (major) = 26.753 min.

**(S)-Methyl 2-hydroxy-6-methyl-1-oxo-2,3-dihydro-1H-indene-2-carboxylate (2l)^2c, 3^**

Prepared according to the general procedure with a reaction time of 1 h. After [column](app:ds:column) [chromatography](app:ds:chromatography) (silica gel, petroleum ether/ethyl acetate = 5/1 ) to give **2l** as white solid; 21.8 mg, 99% yield, 99% ee; [α]_D_^25^ = +52.0 (c 0.20, CHCl_3_); ^1^H NMR (400 MHz, CDCl_3_) δ 7.60 (d, *J* = 1.7 Hz, 1H), 7.50 (dd, *J* = 7.8, 1.8 Hz, 1H), 7.42 – 7.34 (m, 1H), 4.00 (s, 1H), 3.73 (s, 3H), 3.68 (d, *J* = 17.1 Hz, 1H), 3.21 (d, *J* = 17.1 Hz, 1H), 2.42 (s, 3H).

^13^C{^1^H} NMR (101 MHz, CDCl_3_) δ 200.9, 172.1, 149.7, 138.3, 137.6, 133.7, 126.2, 125.2, 80.7, 53.5, 39.0, 21.1.

HPLC conditions: Chiralcel OD-H column (250×4.6 mm), hexane/*i*-PrOH = 90/10, 1 mL/min, 254 nm, τ_R_ (major) = 10.352 min, τ_R_ (minor) = 12.497 min.

**(S)-Methyl 6-chloro-2-hydroxy-1-oxo-2,3-dihydro-1H-indene-2-carboxylate (2m)^2c, 3^**

Prepared according to the general procedure with a reaction time of 1 h. After [column](app:ds:column) [chromatography](app:ds:chromatography) (silica gel, petroleum ether/ethyl acetate = 5/1 ) to give **2m** as colorless oil; 23.3 mg, 97% yield, 97% ee; [α]_D_^25^ = + 28.7 (c 0.14, CHCl_3_); ^1^H NMR (400 MHz, CDCl_3_) δ 7.76 (d, *J* = 2.1 Hz, 1H), 7.63 (dd, *J* = 8.2, 2.1 Hz, 1H), 7.44 (d, *J* = 8.2 Hz, 1H), 4.02 (s, 1H), 3.75 (s, 3H), 3.69 (d, *J* = 17.4 Hz, 1H), 3.22 (d, *J* = 17.4 Hz, 1H).

^13^C{^1^H} NMR (101 MHz, CDCl_3_) δ 199.7, 171.5, 150.2, 136.2, 135.0, 134.6, 127.7, 125.0, 80.8, 53.6, 38.9.

HPLC conditions: Chiralcel AS-H column (250×4.6 mm), hexane/*i*-PrOH = 90/10, 1 mL/min, 254 nm, τ_R_ (major) = 11.835 min, τ_R_ (minor) = 14.308 min.

**(S)-Methyl 6-bromo-2-hydroxy-1-oxo-2,3-dihydro-1H-indene-2-carboxylate (2n)^2c, 3^**

Prepared according to the general procedure with a reaction time of 1 h. After [column](app:ds:column) [chromatography](app:ds:chromatography) (silica gel, petroleum ether/ethyl acetate = 5/1 ) to give **2m** as white solid; 27.9 mg, 98% yield, 99% ee; [α]_D_^25^ = +19.45 (c 0.25, CHCl_3_); ^1^H NMR (400 MHz, CDCl_3_) δ 7.92 (d, *J* = 1.9 Hz, 1H), 7.77 (dd, *J* = 8.1, 1.9 Hz, 1H), 7.39 (d, *J* = 8.2 Hz, 1H), 4.09 (s, 1H), 3.75 (s, 3H), 3.67 (d, *J* = 17.4 Hz, 1H), 3.20 (d, *J* = 17.4 Hz, 1H).

^13^C{^1^H} NMR (101 MHz, CDCl_3_) δ 199.6, 171.5, 150.7, 138.9, 135.4, 128.1, 128.0, 122.3, 80.6, 53.6, 39.0.

HPLC conditions: Chiralcel OD-H column (250×4.6 mm), hexane/*i*-PrOH = 90/10, 1 mL/min, 254 nm, τ_R_ (major) = 20.577 min, τ_R_ (minor) = 26.217 min.

**(S)-Methyl 6-fluoro-2-hydroxy-1-oxo-2,3-dihydro-1H-indene-2-carboxylate (2o)^2c, 3^**

Prepared according to the general procedure with a reaction time of 1 h. After [column](app:ds:column) [chromatography](app:ds:chromatography) (silica gel, petroleum ether/ethyl acetate = 5/1 ) to give **2o** as white solid; 22.0 mg, 98% yield, 98% ee; [α]_D_^25^ = +54.4 (c 0.14, CHCl_3_); ^1^H NMR (400 MHz, CDCl_3_) δ 7.53 – 7.34 (m, 3H), 4.04 (s, 1H), 3.75 (s, 3H), 3.69 (d, *J* = 17.1 Hz, 1H), 3.22 (dt, *J* = 17.0, 1.4 Hz, 1H).

^13^C{^1^H} NMR (101 MHz, CDCl_3_) δ 200.1, 200.0, 171.6, 163.8, 161.3, 147.7, 147.7, 135.3, 135.2, 128.0, 127.9, 124.1, 123.8, 111.2, 110.9, 81.0, 53.6, 38.8.

HPLC conditions: Chiralcel OD-H column (250×4.6 mm), hexane/*i*-PrOH = 90/10, 1 mL/min, 254 nm, τ_R_ (major) = 11.560 min, τ_R_ (minor) = 14.167 min.

**(S)-Methyl 2-hydroxy-6-methoxy-1-oxo-2,3-dihydro-1H-indene-2-carboxylate (2p)^2c, 3^**

Prepared according to the general procedure with a reaction time of 1 h. After [column](app:ds:column) [chromatography](app:ds:chromatography) (silica gel, petroleum ether/ethyl acetate = 5/1 ) to give **2p** as yellowish solid; 23.4 mg, 99% yield, 99% ee; [α]_D_^25^ = +36.2 (c 0.15, CHCl_3_); ^1^H NMR (400 MHz, CDCl_3_) δ 7.31 (d, *J* = 8.4 Hz, 1H), 7.19 (dd, *J* = 8.2, 2.7 Hz, 1H), 7.14 (d, *J* = 2.6 Hz, 1H), 3.97 (s, 1H), 3.77 (s, 3H), 3.67 (s, 3H), 3.58 (d, *J* = 16.9 Hz, 1H), 3.10 (d, *J* = 16.9 Hz, 1H).

^13^C{^1^H} NMR (101 MHz, CDCl_3_) δ 200.8, 172.0, 159.9, 145.2, 134.7, 127.2, 125.7, 106.2, 81.1, 55.7, 53.5, 38.7.

HPLC conditions: Chiralcel OD-H column (250×4.6 mm), hexane/*i*-PrOH = 90/10, 1 mL/min, 254 nm, τ_R_ (major) = 22.915 min, τ_R_ (minor) = 28.263 min.

**(S)-Methyl 2-hydroxy-7-methoxy-1-oxo-2,3-dihydro-1H-indene-2-carboxylate (2q)^2c, 3^**

Prepared according to the general procedure with a reaction time of 1 h. After [column](app:ds:column) [chromatography](app:ds:chromatography) (silica gel, petroleum ether/ethyl acetate = 5/1 ) to give **2q** as colorless solid; 23.4 mg, 99%yield, 99% ee; [α]_D_^25^ = +14.0 (c 0.18, CHCl_3_); ^1^H NMR (400 MHz, CDCl_3_) δ 7.61 (t, *J* = 7.9 Hz, 1H), 7.03 (d, *J* = 7.5 Hz, 1H), 6.84 (d, *J* = 8.2 Hz, 1H), 3.99 (s, 1H), 3.96 (s, 3H), 3.72 (s, 3H), 3.67 (d, *J* = 17.2 Hz, 1H), 3.19 (d, *J* = 17.2 Hz, 1H).

^13^C{^1^H} NMR (101 MHz, CDCl_3_) δ 198.3, 171.8, 159.1, 154.3, 138.1, 121.7, 118.1, 109.6, 80.6, 55.9, 53.4, 38.7.

HPLC conditions: Chiralcel OD-H column (250×4.6 mm), hexane/*i*-PrOH = 90/10, 1.0 mL/min, 254 nm, τ_R_ (major) = 37.138 min, τ_R_ (minor) = 46.032 min.

**(S)-Ethyl 2-hydroxy-1-oxo-2,3-dihydro-1H-indene-2-carboxylate (2r)^2c, 3^**

Prepared according to the general procedure with a reaction time of 1 h. After [column](app:ds:column) [chromatography](app:ds:chromatography) (silica gel, petroleum ether/ethyl acetate = 5/1 ) to give **2r** as yellowish solid; 21.8 mg, 99% yield, 99% ee; [α]_D_^25^ = +26.4 (c 0.20, CHCl_3_); ^1^H NMR (400 MHz, CDCl_3_) δ 7.80 (d, *J* = 7.7 Hz, 1H), 7.67 (td, *J* = 7.5, 1.3 Hz, 1H), 7.49 (dt, *J* = 7.6, 1.0 Hz, 1H), 7.46 – 7.38 (m, 1H), 4.21 (qq, *J* = 7.2, 3.6 Hz, 2H), 4.10 (s, 1H), 3.73 (d, *J* = 17.2 Hz, 1H), 3.25 (d, *J* = 17.3 Hz, 1H), 1.18 (t, *J* = 7.1 Hz, 3H).

^13^C{^1^H} NMR (101 MHz, CDCl_3_) δ 201.0, 171.5, 152.3, 136.1, 133.6, 128.1, 126.5, 125.3, 80.3, 62.8, 39.3, 14.0.

HPLC conditions: Chiralcel OD-H column (250×4.6 mm), hexane/*i*-PrOH = 90/10, 1 mL/min, 254 nm, τ_R_ (major) = 13.290 min, τ_R_ (minor) = 15.783 min.

**(S)-Ethyl 5-chloro-2-hydroxy-1-oxo-2,3-dihydro-1H-indene-2-carboxylate (2s)^2c, 3^**

Prepared according to the general procedure with a reaction time of 1 h. After [column](app:ds:column) [chromatography](app:ds:chromatography) (silica gel, petroleum ether/ethyl acetate = 5/1 ) to give **2s** as colorless oil; 24.7 mg, 99% yield, 99% ee; [α]_D_^25^ = +83.4 (c 0.18, CHCl_3_); ^1^H NMR (400 MHz, CDCl_3_) δ 7.73 (d, *J* = 8.2 Hz, 1H), 7.50 (dt, *J* = 1.6, 0.9 Hz, 1H), 7.45 – 7.37 (m, 1H), 4.22 (qd, *J* = 7.1, 3.2 Hz, 2H), 4.04 (s, 1H), 3.76 – 3.61 (m, 1H), 3.28 – 3.15 (m, 1H), 1.19 (t, *J* = 7.1 Hz, 3H).

^13^C{^1^H} NMR (101 MHz, CDCl_3_) δ 199.5, 171.1, 153.6, 142.8, 132.1, 129.0, 126.7, 126.3, 80.4, 63.0, 39.0, 14.0.

HPLC conditions: Chiralcel OD-H column (250×4.6 mm), hexane/*i*-PrOH = 90/10, 1.0 mL/min, 254 nm, τ_R_ (major) = 10.230 min, τ_R_ (minor) = 12.655 min.

**(S)-Ethyl 2-hydroxy-5-methyl-1-oxo-2,3-dihydro-1H-indene-2-carboxylate (2t)^2c, 3^**

Prepared according to the general procedure with a reaction time of 1 h. After [column](app:ds:column) [chromatography](app:ds:chromatography) (silica gel, petroleum ether/ethyl acetate = 5/1 ) to give **2t** as colorless solid; 23.2 mg, 99%yield, 98% ee; [α]_D_^25^ = +58.0 (c 0.12, CHCl_3_); ^1^H NMR (400 MHz, CDCl_3_) δ 7.69 (d, *J* = 7.9 Hz, 1H), 7.32 – 7.28 (m, 1H), 7.26 – 7.20 (m, 1H), 4.21 (qd, *J* = 7.1, 4.3 Hz, 2H), 4.04 (s, 1H), 3.67 (d, *J* = 17.2 Hz, 1H), 3.20 (d, *J* = 17.2 Hz, 1H), 2.47 (s, 3H), 1.18 (t, *J* = 7.1 Hz, 3H).

^13^C{^1^H} NMR (101 MHz, CDCl_3_) δ 200.4, 171.6, 152.8, 147.7, 131.3, 129.4, 126.8, 125.1, 80.5, 62.7, 39.1, 22.3, 14.0.

HPLC conditions: Chiralcel OD-H column (250×4.6 mm), hexane/*i*-PrOH = 90/10, 1.0 mL/min, 254 nm, τ_R_ (major) = 9.982 min, τ_R_ (minor) = 12.437 min.

**ethyl (S)-2-hydroxy-6-methyl-1-oxo-2,3-dihydro-1H-indene-2-carboxylate (2u)^2c, 3^**

Prepared according to the general procedure with a reaction time of 1 h. After [column](app:ds:column) [chromatography](app:ds:chromatography) (silica gel, petroleum ether/ethyl acetate = 5/1 ) to give **2u** as colorless solid; 23.2 mg, 99%yield, 97% ee; [α]_D_^25^ = +63.2 (c 0.25, CHCl_3_); ^1^H NMR (500 MHz, CDCl_3_) δ 7.52 (s, 1H), 7.41 (dd, *J* = 7.9, 1.6 Hz, 1H), 7.30 (d, *J* = 7.8 Hz, 1H), 4.13 (p, *J* = 6.9 Hz, 2H), 3.97 (d, *J* = 6.8 Hz, 1H), 3.60 (d, *J* = 17.1 Hz, 1H), 3.12 (d, *J* = 17.1 Hz, 1H), 2.34 (s, 3H), 1.11 (t, *J* = 7.1 Hz, 3H).

^13^C{^1^H} NMR (126 MHz, CDCl_3_) δ 201.1, 171.6, 149.7, 138.2, 137.4, 133.7, 126.1, 125.2, 80.7, 62.7, 39.0, 21.1, 14.0.

HPLC conditions: Chiralcel OD-H column (250×4.6 mm), hexane/*i*-PrOH = 90/10, 1.0 mL/min, 254 nm, τ_R_ (major) = 13.453 min, τ_R_ (minor) = 16.270 min.

**(S)-Ethyl 4-chloro-2-hydroxy-1-oxo-2,3-dihydro-1H-indene-2-carboxylate (2v)^2c, 3^**

Prepared according to the general procedure with a reaction time of 1 h. After [column](app:ds:column) [chromatography](app:ds:chromatography) (silica gel, petroleum ether/ethyl acetate = 5/1 ) to give **2v** as colorless solid; 24.7 mg, 97%yield, 99% ee; [α]_D_^25^ = +73.2 (c 0.15, CHCl_3_); ^1^H NMR (400 MHz, CDCl_3_) δ 7.76 – 7.69 (m, 1H), 7.67 (dd, *J* = 7.8, 1.1 Hz, 1H), 7.42 (td, *J* = 7.7, 0.9 Hz, 1H), 4.24 (qd, *J* = 7.1, 1.1 Hz, 2H), 4.11 (s, 1H), 3.72 (d, *J* = 17.8 Hz, 1H), 3.22 (d, *J* = 17.8 Hz, 1H), 1.20 (td, *J* = 7.2, 1.1 Hz, 3H).

^13^C{^1^H} NMR (101 MHz, CDCl_3_) δ 200.2, 171.1, 150.0, 135.7, 135.5, 132.7, 129.7, 123.5, 80.0, 63.1, 38.4, 14.0.

HPLC conditions: Chiralcel OD-H column (250×4.6 mm), hexane/*i*-PrOH = 98/2, 1.0 mL/min, 254 nm, τ_R_ (major) = 19.403 min, τ_R_ (minor) = 21.660 min.

**(S)-Ethyl 4-bromo-2-hydroxy-1-oxo-2,3-dihydro-1H-indene-2-carboxylate (2w)^2c, 3^**

Prepared according to the general procedure with a reaction time of 1 h. After [column](app:ds:column) [chromatography](app:ds:chromatography) (silica gel, petroleum ether/ethyl acetate = 5/1 ) to give **2w** as colorless solid; 29.3 mg, 98%yield, 99% ee; [α]_D_^25^ = +66.3 (c 0.13, CHCl_3_); ^1^H NMR (400 MHz, CDCl_3_) δ 7.84 (dd, *J* = 7.8, 1.0 Hz, 1H), 7.76 (dd, *J* = 7.6, 1.0 Hz, 1H), 7.35 (tt, *J* = 7.7, 0.9 Hz, 1H), 4.24 (qd, *J* = 7.1, 0.7 Hz, 2H), 4.08 (s, 1H), 3.66 (d, *J* = 17.8 Hz, 1H), 3.18 (d, *J* = 17.8 Hz, 1H), 1.20 (t, *J* = 7.1 Hz, 3H).

^13^C{^1^H} NMR (101 MHz, CDCl_3_) δ 200.3, 171.1, 152.0, 138.8, 135.6, 129.9, 124.0, 121.8, 80.0, 63.1, 40.4, 14.0.

HPLC conditions: Chiralcel OD-H column (250×4.6 mm), hexane/*i*-PrOH = 98/2, 1.0 mL/min, 254 nm, τ_R_ (major) = 20.010 min, τ_R_ (minor) = 22.058 min.

**(S)-Ethyl 2-hydroxy-4-methyl-1-oxo-2,3-dihydro-1H-indene-2-carboxylate (2x)^2c, 3^**

Prepared according to the general procedure with a reaction time of 1 h. After [column](app:ds:column) [chromatography](app:ds:chromatography) (silica gel, petroleum ether/ethyl acetate = 5/1 ) to give **2x** as colorless solid; 23.1 mg, 99%yield, 99% ee; [α]_D_^25^ = +40.6 (c 0.15, CHCl_3_); ^1^H NMR (500 MHz, CDCl_3_) δ 7.64 (d, *J* = 7.6 Hz, 1H), 7.48 (d, *J* = 7.3 Hz, 1H), 7.35 (t, *J* = 7.5 Hz, 1H), 4.23 (q, *J* = 7.2 Hz, 2H), 4.11 (s, 1H), 3.63 (d, *J* = 17.2 Hz, 1H), 3.12 (d, *J* = 17.3 Hz, 1H), 2.37 (s, 3H), 1.20 (t, *J* = 7.1 Hz, 3H).

^13^C{^1^H} NMR (126 MHz, CDCl_3_) δ 201.3, 171.6, 151.4, 136.6, 135.8, 133.3, 128.3, 122.7, 80.2, 62.7, 38.3, 17.8, 14.0.

HPLC conditions: Chiralcel OD-H column (250×4.6 mm), hexane/*i*-PrOH = 90/10, 1.0 mL/min, 254 nm, τ_R_ (major) = 23.515 min, τ_R_ (minor) = 28.175 min.

**(S)-Ethyl 2-hydroxy-6-methoxy-1-oxo-2,3-dihydro-1H-indene-2-carboxylate (2y)^2c, 3^**

Prepared according to the general procedure with a reaction time of 1 h. After [column](app:ds:column) [chromatography](app:ds:chromatography) (silica gel, petroleum ether/ethyl acetate = 5/1 ) to give **2y** as colorless solid; 24.5 mg, 98%yield, 98% ee; [α]_D_^25^ = +39.5.0 (c 0.10, CHCl_3_); ^1^H NMR (400 MHz, CDCl_3_) δ 7.38 (d, *J* = 8.4 Hz, 1H), 7.26 (dd, *J* = 8.4, 2.5 Hz, 1H), 7.22 (d, *J* = 2.6 Hz, 1H), 4.22 (qq, *J* = 7.3, 3.7 Hz, 2H), 4.03 (s, 1H), 3.85 (s, 3H), 3.64 (d, *J* = 16.9 Hz, 1H), 3.17 (d, *J* = 16.9 Hz, 1H), 1.19 (t, *J* = 7.1 Hz, 3H).

^13^C{^1^H} NMR (101 MHz, CDCl_3_) δ 201.0, 171.6, 159.8, 145.3, 134.7, 127.2, 125.6, 106.2, 81.0, 62.8, 55.7, 38.7, 14.0.

HPLC conditions: Chiralcel OD-H column (250×4.6 mm), hexane/*i*-PrOH = 90/10, 1.0 mL/min, 254 nm, τ_R_ (major) = 11.772 min, τ_R_ (minor) = 13.417 min.

**isopropyl (S)-2-hydroxy-1-oxo-2,3-dihydro-1H-indene-2-carboxylate (2z)^2c, 3^**

Prepared according to the general procedure with a reaction time of 1 h. After [column](app:ds:column) [chromatography](app:ds:chromatography) (silica gel, petroleum ether/ethyl acetate = 5/1 ) to give **2z** as colorless solid; 23.2 mg, 99%yield, 99% ee; [α]_D_^25^ = +73.2 (c 0.22, CHCl_3_);  ^1^H NMR (400 MHz, CDCl_3_) δ 7.80 (d, *J* = 7.7 Hz, 1H), 7.67 (td, *J* = 7.5, 1.3 Hz, 1H), 7.49 (dt, *J* = 7.7, 1.0 Hz, 1H), 7.46 – 7.37 (m, 1H), 5.07 (hept, *J* = 6.3 Hz, 1H), 4.03 (s, 1H), 3.70 (d, *J* = 17.2 Hz, 1H), 3.24 (d, *J* = 17.2 Hz, 1H), 1.20 (d, *J* = 6.3 Hz, 3H), 1.13 (d, *J* = 6.3 Hz, 3H).

^13^C{^1^H} NMR (101 MHz, CDCl_3_) δ 201.0, 171.0, 152.4, 136.0, 133.7, 128.1, 126.4, 125.2, 80.3, 70.9, 39.3, 21.5, 21.3.

HPLC conditions: Chiralcel OD-H column (250×4.6 mm), hexane/*i*-PrOH = 90/10, 1.0 mL/min, 254 nm, τ_R_ (major) = 7.852 min, τ_R_ (minor) = 8.685 min.

**(S)-Isopropyl 5-chloro-2-hydroxy-1-oxo-2,3-dihydro-1H-indene-2-carboxylate (2aa)^2c, 3^**

Prepared according to the general procedure with a reaction time of 1 h. After [column](app:ds:column) [chromatography](app:ds:chromatography) (silica gel, petroleum ether/ethyl acetate = 5/1 ) to give **2aa** as colorless solid; 26.6 mg, 99%yield, 99% ee; [α]_D_^25^ = +75.3 (c 0.20, CHCl_3_); ^1^H NMR (400 MHz, CDCl_3_) δ 7.73 (d, *J* = 8.2 Hz, 1H), 7.49 (d, *J* = 1.7 Hz, 1H), 7.41 (dd, *J* = 8.2, 1.8 Hz, 1H), 5.07 (hept, *J* = 6.2 Hz, 1H), 4.06 (s, 1H), 3.66 (d, *J* = 17.4 Hz, 1H), 3.22 (d, *J* = 17.4 Hz, 1H), 1.21 (d, *J* = 6.3 Hz, 3H), 1.14 (d, *J* = 6.3 Hz, 3H).

^13^C{^1^H} NMR (101 MHz, CDCl_3_) δ 199.6, 170.6, 153.7, 142.7, 132.1, 129.0, 126.7, 126.3, 80.3, 71.1, 39.0, 21.6, 21.3.

HPLC conditions: Chiralcel OD-H column (250×4.6 mm), hexane/*i*-PrOH = 90/10, 1.0 mL/min, 254 nm, τ_R_ (major) = 11.510 min, τ_R_ (minor) = 13.985 min.

**benzyl (S)-5-chloro-2-hydroxy-1-oxo-2,3-dihydro-1H-indene-2-carboxylate (2ab)^2c, 3^**

Prepared according to the general procedure with a reaction time of 1 h. After [column](app:ds:column) [chromatography](app:ds:chromatography) (silica gel, petroleum ether/ethyl acetate = 5/1 ) to give **2ab** as colorless solid; 31.4 mg, 99%yield, 99% ee; [α]_D_^25^ = +38.4 (c 0.16, CHCl_3_); ^1^H NMR (400 MHz, CDCl_3_) δ 7.64 (d, *J* = 8.2 Hz, 1H), 7.39 (s, 1H), 7.32 (dd, *J* = 8.2, 1.7 Hz, 1H), 7.23 (dd, *J* = 4.9, 1.9 Hz, 3H), 7.08 (dd, *J* = 6.8, 2.9 Hz, 2H), 5.15 (d, *J* = 12.3 Hz, 1H), 5.04 (d, *J* = 12.3 Hz, 1H), 3.99 (s, 1H), 3.60 (d, *J* = 17.4 Hz, 1H), 3.14 (d, *J* = 17.4 Hz, 1H).

^13^C{^1^H} NMR (101 MHz, CDCl_3_) δ 198.3, 169.9, 152.5, 141.8, 133.6, 131.0, 128.0, 127.6, 127.6, 126.8, 125.7, 125.3, 79.6, 67.2, 37.9.

HPLC conditions: Chiralcel OD-H column (250×4.6 mm), hexane/*i*-PrOH = 90/10, 1.0 mL/min, 254 nm, τ_R_ (major) = 26.618 min, τ_R_ (minor) = 34.592 min.

**benzyl (S)-2-hydroxy-1-oxo-2,3-dihydro-1H-indene-2-carboxylate (2ac)^2c, 3^**

Prepared according to the general procedure with a reaction time of 1 h. After [column](app:ds:column) [chromatography](app:ds:chromatography) (silica gel, petroleum ether/ethyl acetate = 5/1 ) to give **2ac** as colorless solid; 27.9 mg, 99%yield, 99% ee; [α]_D_^25^ = +48.2 (c 0.37, CHCl_3_); ^1^H NMR (400 MHz, CDCl_3_) δ 7.79 (dt, *J* = 7.6, 1.0 Hz, 1H), 7.64 (td, *J* = 7.5, 1.2 Hz, 1H), 7.46 (dt, *J* = 7.8, 1.0 Hz, 1H), 7.41 (td, *J* = 7.4, 0.9 Hz, 1H), 7.33 – 7.24 (m, 3H), 7.17 – 7.07 (m, 2H), 5.21 (d, *J* = 12.4 Hz, 1H), 5.11 (d, *J* = 12.4 Hz, 1H), 4.10 (s, 1H), 3.71 (d, *J* = 17.2 Hz, 1H), 3.24 (d, *J* = 17.2 Hz, 1H).

^13^C{^1^H} NMR (101 MHz, CDCl_3_) δ 200.8, 171.3, 152.2, 136.2, 134.8, 133.6, 128.6, 128.5, 128.2, 127.8, 126.5, 125.3, 80.6, 68.0, 39.3.

HPLC conditions: Chiralcel OD-H column (250×4.6 mm), hexane/*i*-PrOH = 90/10, 1.0 mL/min, 254 nm, τ_R_ (major) = 24.028 min, τ_R_ (minor) = 30.157 min.

**tert-butyl (S)-5-chloro-2-hydroxy-1-oxo-2,3-dihydro-1H-indene-2-carboxylate (2ad)^2c, 3^**

Prepared according to the general procedure with a reaction time of 1 h. After [column](app:ds:column) [chromatography](app:ds:chromatography) (silica gel, petroleum ether/ethyl acetate = 5/1 ) to give **2ad** as a colorless solid, 28.0 mg, 99%yield, 99% ee; [α]_D_^25^ = +45.6 (c 0.20, CHCl_3_); ^1^H NMR (400 MHz, CDCl_3_) δ 7.72 (d, *J* = 8.2 Hz, 1H), 7.48 (s, 1H), 7.40 (dd, *J* = 8.2, 1.8 Hz, 1H), 4.06 (s, 1H), 3.62 (d, *J* = 17.3 Hz, 1H), 3.20 (d, *J* = 17.3 Hz, 1H), 1.37 (s, 9H).

^13^C{^1^H} NMR (101 MHz, CDCl_3_) δ 199.9, 170.2, 153.7, 142.5, 132.4, 128.8, 126.6, 126.1, 84.3, 80.5, 39.2, 27.7.

HPLC conditions: Chiralcel OD-H column (250×4.6 mm), hexane/*i*-PrOH = 98/2, 1.0 mL/min, 254 nm, τ_R_ (major) = 8.173 min, τ_R_ (minor) = 8.992 min.

**(3S)-adamantan-1-yl(2S)-5-chloro-2-hydroxy-1-oxo-2,3-dihydro-1H-indene-2-carboxylate(2ae)^2c, 3^**

Prepared according to the general procedure with a reaction time of 1 h. After [column](app:ds:column) [chromatography](app:ds:chromatography) (silica gel, petroleum ether/ethyl acetate = 5/1 ) to give **2ae** as colorless solid; 35.7 mg, 98%yield, 96% ee; [α]_D_^25^ = +84.3 (c 0.24, CHCl_3_); ^1^H NMR (400 MHz, CDCl_3_) δ 7.65 (d, *J* = 8.2 Hz, 1H), 7.41 (s, 1H), 7.33 (dd, *J* = 8.3, 1.7 Hz, 1H), 3.96 (s, 1H), 3.55 (d, *J* = 17.3 Hz, 1H), 3.12 (d, *J* = 17.3 Hz, 1H), 2.06 (d, *J* = 4.4 Hz, 3H), 1.89 (d, *J* = 3.0 Hz, 6H), 1.53 (d, *J* = 3.1 Hz, 6H).

^13^C{^1^H} NMR (101 MHz, CDCl_3_) δ 200.0, 169.8, 153.7, 142.4, 132.5, 128.8, 126.5, 126.1, 84.3, 80.5, 40.9, 39.3, 35.8, 30.8.

HPLC conditions: Chiralcel AD-H column (250×4.6 mm), hexane/*i*-PrOH = 90/10, 1.0 mL/min, 254 nm, τ_R_ (major) = 17.553 min, τ_R_ (minor) = 29.010 min.

**(3S)-adamantan-1-yl(2S)-2-hydroxy-4-methoxy-1-oxo-2,3-dihydro-1H-indene-2-carboxylate(2af)^2c, 3^**

Prepared according to the general procedure with a reaction time of 1 h. After [column](app:ds:column) [chromatography](app:ds:chromatography) (silica gel, petroleum ether/ethyl acetate = 5/1 ) to give **2af** as colorless solid; 34.9 mg, 98%yield, 98% ee; [α]_D_^25^ = +39.4 (c 0.10, CHCl_3_); ^1^H NMR (400 MHz, CDCl_3_) δ 7.39 (d, *J* = 4.4 Hz, 2H), 7.15 – 7.07 (m, 1H), 4.95 (t, *J* = 3.5 Hz, 1H), 4.01 (s, 1H), 3.91 (s, 3H), 3.66 (d, *J* = 17.4 Hz, 1H), 3.13 (d, *J* = 17.4 Hz, 1H), 1.95 – 1.82 (m, 2H), 1.81 – 1.66 (m, 5H), 1.64 – 1.56 (m, 3H), 1.43 (q, *J* = 12.8 Hz, 2H), 1.31 (d, *J* = 4.1 Hz, 2H).

^13^C{^1^H} NMR (101 MHz, CDCl_3_) δ 201.3, 170.8, 156.7, 141.0, 135.4, 129.5, 116.5, 116.2, 80.9, 79.7, 55.7, 37.1, 36.3, 36.1, 36.0, 31.7, 31.5, 31.5, 31.3, 26.9, 26.7.

HPLC conditions: Chiralcel aD-H column (250×4.6 mm), hexane/*i*-PrOH = 90/10, 1.0 mL/min, 254 nm, τ_R_ (major) = 24.633 min, τ_R_ (minor) = 33.795 min.

**(3S)-adamantan-1-yl (2S)-2-hydroxy-1-oxo-2,3-dihydro-1H-indene-2-carboxylate (2ag)^2c, 3^**

Prepared according to the general procedure with a reaction time of 1 h. After [column](app:ds:column) [chromatography](app:ds:chromatography) (silica gel, petroleum ether/ethyl acetate = 5/1 ) to give **2ag** as colorless solid; 32 mg, 98%yield, 90% ee; [α]_D_^25^ = +33.2 (c 0.20, CHCl_3_); ^1^H NMR (400 MHz, CDCl_3_) δ 7.79 (d, *J* = 7.7 Hz, 1H), 7.65 (td, *J* = 7.5, 1.2 Hz, 1H), 7.47 (d, *J* = 7.7 Hz, 1H), 7.41 (td, *J* = 7.5, 1.0 Hz, 1H), 4.04 (s, 1H), 3.66 (d, *J* = 17.1 Hz, 1H), 3.22 (d, *J* = 17.1 Hz, 1H), 2.19 – 2.06 (m, 3H), 1.96 (d, *J* = 3.0 Hz, 6H), 1.59 (t, *J* = 3.0 Hz, 6H).

^13^C{^1^H} NMR (101 MHz, CDCl_3_) δ 201.5, 170.2, 152.4, 135.9, 134.0, 127.9, 126.3, 125.1, 84.0, 80.5, 40.9, 39.6, 35.9, 30.8.

HPLC conditions: Chiralcel AD-H column (250×4.6 mm), hexane/*i*-PrOH = 90/10, 1.0 mL/min, 254 nm, τ_R_ (major) = 16.303 min, τ_R_ (minor) = 26.235 min.

**(S)-5-chloro-2-hydroxy-1-oxo-N-phenyl-2,3-dihydro-1H-indene-2-carboxamide (2ah)^2c, 3^**

Prepared according to the general procedure with a reaction time of 1 h. After [column](app:ds:column) [chromatography](app:ds:chromatography) (silica gel, petroleum ether/ethyl acetate = 5/1 ) to give **2ah** as colorless solid; 29.6 mg, 98%yield, 90% ee; [α]_D_^25^ = +42.6 (c 0.25, CHCl_3_); ^1^H NMR (500 MHz, DMSO) δ 9.91 (s, 1H), 7.81 – 7.60 (m, 4H), 7.55 (dd, *J* = 8.2, 1.9 Hz, 1H), 7.31 (t, *J* = 7.8 Hz, 2H), 7.15 – 7.05 (m, 2H), 3.75 (d, *J* = 17.5 Hz, 1H), 3.15 (d, *J* = 17.5 Hz, 1H).

^13^C{^1^H} NMR (126 MHz, DMSO) δ 201.6, 169.7, 154.8, 140.8, 137.9, 132.6, 128.6, 128.4, 126.8, 125.7, 123.9, 120.0, 81.2, 40.7.

HPLC conditions: Chiralcel OD-H column (250×4.6 mm), hexane/*i*-PrOH = 90/10, 1.0 mL/min, 254 nm, τ_R_ (major) = 24.753 min, τ_R_ (minor) = 41.315 min.

**(S)-N-(tert-butyl)-5-chloro-2-hydroxy-1-oxo-2,3-dihydro-1H-indene-2-carboxamide (2ai)^2c, 3^**

Prepared according to the general procedure with a reaction time of 1 h. After [column](app:ds:column) [chromatography](app:ds:chromatography) (silica gel, petroleum ether/ethyl acetate = 5/1 ) to give **2ai** as colorless solid; 24.8 mg, 88%yield, 72% ee; [α]_D_^25^ = -7.33 (c 0.20, CHCl_3_); ^1^H NMR (400 MHz, CDCl_3_) δ 7.68 (d, *J* = 8.2 Hz, 1H), 7.47 (d, *J* = 1.9 Hz, 1H), 7.37 (dd, *J* = 8.3, 1.8 Hz, 1H), 6.71 (s, 1H), 3.75 – 3.57 (m, 2H), 3.04 (d, *J* = 16.8 Hz, 1H), 1.33 (s, 9H).

^13^C{^1^H} NMR (101 MHz, CDCl_3_) δ 202.4, 169.1, 154.5, 142.9, 132.5, 128.8, 128.8, 126.6, 126.0, 126.0, 82.5, 51.5, 40.5, 29.7, 28.5.

HPLC conditions: Chiralcel AS-H column (250×4.6 mm), hexane/*i*-PrOH = 90/10, 1.0 mL/min, 254 nm, τ_R_ (minor) = 7.365 min, τ_R_ (major) = 26.137 min.

**(S)-5-chloro-2-hydroxy-N-isopropyl-1-oxo-2,3-dihydro-1H-indene-2-carboxamide (2aj)^2c, 3^**

Prepared according to the general procedure with a reaction time of 1 h. After [column](app:ds:column) [chromatography](app:ds:chromatography) (silica gel, petroleum ether/ethyl acetate = 5/1 ) to give **2aj** as colorless solid; 23.0 mg, 86%yield, 73% ee; [α]_D_^25^ = -8.10 (c 0.12, CHCl_3_); ^1^H NMR (400 MHz, CDCl_3_) δ 7.63 (d, *J* = 8.2 Hz, 1H), 7.44 (d, *J* = 1.6 Hz, 1H), 7.39 – 7.29 (m, 1H), 6.77 (d, *J* = 8.0 Hz, 1H), 4.17 (s, 1H), 3.99 – 3.80 (m, 1H), 3.65 (d, *J* = 17.0 Hz, 1H), 3.00 (dd, *J* = 17.0, 1.2 Hz, 1H), 1.13 (t, *J* = 6.8 Hz, 6H).

^13^C{^1^H} NMR (101 MHz, CDCl_3_) δ 202.0, 169.2, 154.5, 142.8, 132.4, 128.7, 126.6, 126.0, 82.0, 41.7, 40.3, 40.3, 22.5, 22.4.

HPLC conditions: Chiralcel OD-H column (250×4.6 mm), hexane/*i*-PrOH = 90/10, 1.0 mL/min, 254 nm, τ_R_ (minor) = 8.383 min, τ_R_ (major) = 9.232 min.

**(S)-N-butyl-5-chloro-2-hydroxy-1-oxo-2,3-dihydro-1H-indene-2-carboxamide (2ak)^2c, 3^**

Prepared according to the general procedure with a reaction time of 1 h. After [column](app:ds:column) [chromatography](app:ds:chromatography) (silica gel, petroleum ether/ethyl acetate = 5/1 ) to give **2ak** as colorless solid; 23.7 mg, 84%yield, 59% ee; [α]_D_^25^ = +11.5 (c 0.14, CHCl_3_); ^1^H NMR (400 MHz, CDCl_3_) δ 7.64 (dd, *J* = 8.2, 6.4 Hz, 1H), 7.46 – 7.41 (m, 1H), 7.36 (dd, *J* = 8.3, 1.9 Hz, 1H), 6.93 (t, *J* = 6.1 Hz, 1H), 4.16 (s, 1H), 3.67 (d, *J* = 16.9 Hz, 1H), 3.17 (qd, *J* = 6.9, 2.9 Hz, 2H), 3.01 (dd, *J* = 17.0, 3.9 Hz, 1H), 1.53 – 1.41 (m, 2H), 1.31 (qd, *J* = 7.4, 2.5 Hz, 2H), 0.90 (t, *J* = 7.3 Hz, 3H).

^13^C{^1^H} NMR (101 MHz, CDCl_3_) δ 201.9, 170.0, 154.4, 142.8, 132.3, 128.7, 126.6, 126.6, 126.0, 126.0, 82.2, 40.4, 40.3, 39.3, 31.4, 19.9, 13.7.

HPLC conditions: Chiralcel OD-H column (250×4.6 mm), hexane/*i*-PrOH = 90/10, 1.0 mL/min, 254 nm, τ_R_ (minor) = 11.435 min, τ_R_ (major) = 12.933 min.

**methyl (S)-2-hydroxy-1-oxo-1,2,3,4-tetrahydronaphthalene-2-carboxylate (2al)^2c, 3^**

Prepared according to the general procedure with a reaction time of 1 h. After [column](app:ds:column) [chromatography](app:ds:chromatography) (silica gel, petroleum ether/ethyl acetate = 5/1 ) to give **2al** as colorless solid; 18.7 mg, 85%yield, 81% ee; [α]_D_^25^ = -16.0 (c 0.20, CHCl_3_); ^1^H NMR (400 MHz, CDCl_3_) δ 8.05 (dd, *J* = 7.9, 1.5 Hz, 1H), 7.53 (td, *J* = 7.5, 1.5 Hz, 1H), 7.40 – 7.31 (m, 1H), 7.27 (d, *J* = 7.8 Hz, 1H), 4.36 (s, 1H), 3.75 (s, 3H), 3.17 – 3.09 (m, 2H), 2.72 (dt, *J* = 13.5, 5.0 Hz, 1H), 2.25 (ddd, *J* = 13.6, 9.0, 6.2 Hz, 1H).

^13^C{^1^H} NMR (126 MHz, CDCl_3_) δ 194.5, 171.0, 144.0, 134.4, 130.2, 129.0, 128.2, 127.0, 77.7, 53.0, 53.0, 32.7, 25.6.

HPLC conditions: Chiralcel OD-H column (250×4.6 mm), hexane/*i*-PrOH = 98/2, 1.0 mL/min, 254 nm, τ_R_ (major) = 20.472 min, τ_R_ (minor) = 23.933 min.

**ethyl (S)-2-hydroxy-1-oxo-1,2,3,4-tetrahydronaphthalene-2-carboxylate (2 am)^2c, 3^**

Prepared according to the general procedure with a reaction time of 1 h. After [column](app:ds:column) [chromatography](app:ds:chromatography) (silica gel, petroleum ether/ethyl acetate = 5/1 ) to give **2am** as colorless solid; 19.4 mg, 83%yield, 81% ee; [α]_D_^25^ = -10.3 (c 0.15, CHCl_3_); ^1^H NMR (500 MHz, CDCl_3_) δ 8.05 (d, *J* = 7.9 Hz, 1H), 7.60 – 7.48 (m, 1H), 7.35 (t, *J* = 7.5 Hz, 1H), 7.30 – 7.21 (m, 1H), 4.30 (s, 1H), 4.21 (qq, *J* = 6.9, 3.6 Hz, 2H), 3.13 (dd, *J* = 7.5, 4.5 Hz, 2H), 2.72 (dt, *J* = 13.5, 5.1 Hz, 1H), 2.33 – 2.19 (m, 1H), 1.21 (td, *J* = 7.0, 0.9 Hz, 3H).2

^13^C{^1^H} NMR (126 MHz, CDCl_3_) δ 194.6, 170.7, 144.0, 134.3, 130.3, 128.9, 128.2, 127.0, 77.6, 62.2, 32.7, 25.6, 14.0.

HPLC conditions: Chiralcel OD-H column (250×4.6 mm), hexane/*i*-PrOH = 98/2, 1.0 mL/min, 254 nm, τ_R_ (major) = 15.528 min, τ_R_ (minor) = 17.693 min.

**methyl (S)-6-bromo-2-hydroxy-1-oxo-1,2,3,4-tetrahydronaphthalene-2-carboxylate (2an)^2c, 3^**

Prepared according to the general procedure with a reaction time of 1 h. After [column](app:ds:column) [chromatography](app:ds:chromatography) (silica gel, petroleum ether/ethyl acetate = 5/1 ) to give **2an** as colorless solid; 26.3 mg, 88%yield, 81% ee; [α]_D_^25^ = -11.4 (c 0.11, CHCl_3_); ^1^H NMR (400 MHz, CDCl_3_) δ 7.90 (d, *J* = 8.4 Hz, 1H), 7.58 – 7.43 (m, 2H), 4.26 (s, 1H), 3.76 (s, 3H), 3.10 (dd, *J* = 7.2, 4.4 Hz, 2H), 2.69 (dt, *J* = 13.7, 5.2 Hz, 1H), 2.25 (ddd, *J* = 13.6, 8.4, 6.6 Hz, 1H).

^13^C{^1^H} NMR (101 MHz, CDCl_3_) δ 193.7, 170.9, 145.6, 131.9, 130.6, 130.0, 129.8, 129.0, 77.5, 53.2, 32.5, 25.3.

HPLC conditions: Chiralcel OD-H column (250×4.6 mm), hexane/*i*-PrOH = 98/2, 1.0 mL/min, 254 nm, τ_R_ (major) = 23.590 min, τ_R_ (minor) = 22.753 min.

**methyl (S)-6-fluoro-2-hydroxy-1-oxo-1,2,3,4-tetrahydronaphthalene-2-carboxylate (2ao)^2c, 3^**

Prepared according to the general procedure with a reaction time of 1 h. After [column](app:ds:column) [chromatography](app:ds:chromatography) (silica gel, petroleum ether/ethyl acetate = 5/1 ) to give **2ao** as colorless solid; 20.7 mg, 87%yield, 81% ee; [α]_D_^25^ = -8.4 (c 0.18, CHCl_3_); ^1^H NMR (400 MHz, CDCl_3_) δ 8.08 (dd, *J* = 8.7, 5.9 Hz, 1H), 7.04 (td, *J* = 8.5, 2.5 Hz, 1H), 6.95 (dd, *J* = 9.1, 2.5 Hz, 1H), 4.36 (s, 1H), 3.76 (s, 3H), 3.12 (dd, *J* = 7.3, 4.6 Hz, 2H), 2.70 (dt, *J* = 13.6, 5.2 Hz, 1H), 2.26 (ddd, *J* = 13.5, 8.3, 6.6 Hz, 1H).

^19^F NMR (377 MHz, CDCl_3_) δ -102.57 (td, *J* = 8.6, 5.7 Hz).

^13^C{^1^H} NMR (101 MHz, CDCl_3_) δ 193.0, 171.1, 167.6, 165.1, 147.3, 147.2, 131.5, 131.4, 126.8, 126.8, 115.5, 115.2, 115.1, 114.8, 77.5, 53.1, 32.5, 25.7, 25.7.

HPLC conditions: Chiralcel OD-H column (250×4.6 mm), hexane/*i*-PrOH = 98/2, 1.0 mL/min, 254 nm, τ_R_ (major) = 21.522 min, τ_R_ (minor) = 25.157 min.

**methyl (S)-7-bromo-2-hydroxy-1-oxo-1,2,3,4-tetrahydronaphthalene-2-carboxylate (2ap)^2c, 3^**

Prepared according to the general procedure with a reaction time of 1 h. After [column](app:ds:column) [chromatography](app:ds:chromatography) (silica gel, petroleum ether/ethyl acetate = 5/1 ) to give **2ap** as colorless solid; 26.3 mg, 88%yield, 91% ee; [α]_D_^25^ = +7.81 (c 0.14, CHCl_3_); ^1^H NMR (400 MHz, CDCl_3_) δ 8.16 (d, *J* = 2.2 Hz, 1H), 7.64 (dd, *J* = 8.2, 2.2 Hz, 1H), 7.16 (d, *J* = 8.2 Hz, 1H), 4.22 (s, 1H), 3.76 (s, 3H), 3.07 (dd, *J* = 7.4, 5.2 Hz, 2H), 2.69 (dt, *J* = 13.6, 5.2 Hz, 1H), 2.24 (dt, *J* = 13.7, 7.5 Hz, 1H).

^13^C{^1^H} NMR (101 MHz, CDCl_3_) δ 193.3, 170.9, 142.7, 137.2, 131.7, 130.9, 130.7, 121.0, 77.5, 53.2, 32.5, 25.1.

HPLC conditions: Chiralcel OD-H column (250×4.6 mm), hexane/*i*-PrOH = 98/2, 1.0 mL/min, 254 nm, τ_R_ (major) = 21.338 min, τ_R_ (minor) = 24.002 min.

**methyl (S)-7-fluoro-2-hydroxy-1-oxo-1,2,3,4-tetrahydronaphthalene-2-carboxylate (2aq)^2c, 3^**

Prepared according to the general procedure with a reaction time of 1 h. After [column](app:ds:column) [chromatography](app:ds:chromatography) (silica gel, petroleum ether/ethyl acetate = 5/1 ) to give **2aq** as colorless solid; 20.5 mg, 86%yield, 81% ee; [α]_D_^25^ = +9.20 (c 0.11, CHCl_3_); ^1^H NMR (400 MHz, CDCl_3_) δ 7.74 – 7.66 (m, 1H), 7.31 – 7.22 (m, 2H), 4.24 (s, 1H), 3.76 (s, 3H), 3.09 (t, *J* = 6.3 Hz, 2H), 2.69 (dt, *J* = 13.7, 5.2 Hz, 1H), 2.25 (ddd, *J* = 13.7, 8.0, 6.6 Hz, 1H).

^19^F NMR (377 MHz, CDCl_3_) δ -114.45.

^13^C{^1^H} NMR (101 MHz, CDCl_3_) δ 193.6, 193.6, 171.0, 162.8, 160.4, 139.8, 139.7, 131.7, 131.6, 130.9, 130.8, 122.0, 121.8, 114.1, 113.9, 77.5, 77.5, 53.1, 32.7, 24.9.

HPLC conditions: Chiralcel OD-H column (250×4.6 mm), hexane/*i*-PrOH = 98/2, 1.0 mL/min, 254 nm, τ_R_ (major) = 18.897 min, τ_R_ (minor) = 20.452 min.

**methyl (S)-5-bromo-2-hydroxy-1-oxo-1,2,3,4-tetrahydronaphthalene-2-carboxylate (2ar)^2c, 3^**

Prepared according to the general procedure with a reaction time of 1 h. After [column](app:ds:column) [chromatography](app:ds:chromatography) (silica gel, petroleum ether/ethyl acetate = 5/1 ) to give **2ar** as colorless solid; 26.0 mg, 87%yield, 82% ee; [α]_D_^25^ = -10.3 (c 0.14, CHCl_3_); ^1^H NMR (400 MHz, CDCl_3_) δ 8.03 (dd, *J* = 7.8, 1.3 Hz, 1H), 7.80 (dd, *J* = 7.9, 1.3 Hz, 1H), 7.24 (t, *J* = 7.8 Hz, 1H), 4.27 (s, 1H), 3.75 (s, 3H), 3.27 – 3.13 (m, 1H), 3.12 – 2.99 (m, 1H), 2.75 (dt, *J* = 13.9, 5.1 Hz, 1H), 2.24 (ddd, *J* = 13.8, 9.6, 5.6 Hz, 1H).

^13^C{^1^H} NMR (101 MHz, CDCl_3_) δ 193.8, 170.6, 143.0, 138.3, 132.0, 128.1, 127.5, 124.8, 53.1, 31.7, 26.5.

HPLC conditions: Chiralcel OD-H column (250×4.6 mm), hexane/*i*-PrOH = 98/2, 1.0 mL/min, 254 nm, τ_R_ (major) = 19.627 min, τ_R_ (minor) = 26.690 min.

## Mechanism Study

**Formation of Zr(IV)-L6 complex**

A dried 10mL Schlenk tube equipped with a magnetic stir bar was charged with Zr(acac)4 (2.881 mg, 0.01 mmol), the salan ligand **L16** (3.7227 mg, 0.011 mmol), and cumene (2 mL). The mixture was stirred at 50°C for 30 min.

**Zr(IV)-L6 complex**

Figure S1. Molecular ion peak of **L16** in Zr(IV)-**L16** complex

Figure S2. Local amplification of the molecular ion peak of Zr(IV)-**L16** complex

Figure S3. Local amplification of the molecular ion peak of Zr(IV)-**L16** complex and **1aa**’.

Figure S4. Local amplification of the molecular ion peak of Zr(IV)-**L29** complex

Figure S5. Local amplification of the molecular ion peak of Zr(IV)-**L29** complex and **1aa**’.

## The gram-scale experiment of substrate 1a.*^a^*

| entry | solvent | Catalyst loading | Temperature (℃) | time (min) | yield (%)*^b^* | ee (%)*^c^* |
| --- | --- | --- | --- | --- | --- | --- |
| 1 | CM | 3.0 mol% | 70 | 40 | 99 | 99 |
| 2 | EA | 2.0 mol% | 50 | 240 | 99 | 99 |

^[a]^ Reaction conditions: Zr(acac)_4_ (1.0 mol%), ligand L16/L29 (3.3 mol%/2.0 mol%) were dissolved in 60 mL solvent and stirred at 50 °C for 30 mins. Then, substrate 1a (5 mmol, 1.12 g ) was dissolved in solvent 60 mL, and the CHP (1.5 equiv) was added at once.

^[b]^ Isolated yield by filtering.

^[c]^ Determined by HPLC on a chiral stationary phase.

## Transformation of chiral Hydroxylation product

Under a nitrogen atmosphere, 2a (0.24 g, 1 mmol), toluene (30 mL), and Et₃N (0.25 g, 2.0 equiv.) were successively added to a 50 mL Schlenk flask. Phenylacetyl chloride (0.20 g, 1.6 mmol) was then slowly introduced under continued nitrogen protection. The reaction mixture was gradually heated to reflux and stirred for 1.5 hours, with the reaction progress monitored by TLC. After completion, the system was cooled to room temperature. The organic layer was washed successively with water (4 × 20 mL) and a saturated brine solution, dried over sodium sulfate (Na₂SO₄), and then concentrated under reduced pressure. The crude product was purified by column chromatography (eluent: PE/EA = 5:1) to afford the target compound 3 (0.35 g, 98% yield, 99% ee). [α]_D_ ^25^ = +75.7 (c = 0.3, MeOH); ^1^H NMR (500 MHz, CDCl_3_) δ 7.73 (d, *J* = 8.2 Hz, 1H), 7.48 – 7.42 (m, 1H), 7.39 (dd, *J* = 8.3, 1.8 Hz, 1H), 7.35 – 7.24 (m, 5H), 4.06 (d, *J* = 17.7 Hz, 1H), 3.85 – 3.74 (m, 2H), 3.71 (s, 3H), 3.20 (d, *J* = 17.7 Hz, 1H). ^13^C NMR (126 MHz, CDCl_3_) δ 193.80, 170.83, 166.51, 152.85, 143.03, 132.82, 131.76, 129.36, 129.15, 128.73, 128.69, 128.66, 127.42, 126.61, 126.41, 84.30, 53.55, 53.53, 40.58, 40.56, 40.54, 38.43. HPLC conditions: Chiralcel OD-H column (250 × 4.6 mm), hexane / i-PrOH = 90 / 10, 1 mL/min, 254 nm, τR (major) = 21.0 min, τR (minor) =15.4 min. HRMS (ESI/[M+H]^+^) Calcd. for C_19_H_16_O_5_Cl requires m/z 359.0686, found m/z 359.0688.

## Computation details

The B3PW91 functional^5^ planted in the Gaussian 16 program^6^ was used for geometrical optimization and subsequent frequency calculations without any symmetry or geometrical constraints. In these calculations, the 6-31G(d) basis set^7^ was used for C, H, N and O atoms, and the Stuttgart/Dresden relativistic effective core potential (Lanl2dz) as well as the associated valence basis set was applied for Zr atom. Grimme’s DFT-D3 dispersion correction was also included in the single-point energy calculations.^8^ To obtain more accurate energies, single-point energy calculations were performed with a larger basis set. In such single-point calculations, the M062X functional, which often shows better performance in the treatment of transition-metal systems, was used. In these single-point calculations, the M062X functional, which often shows better performance in the treatment of transition-metal systems, was used. The same SDD/Los Alamos ECP, along with the associated basis set, was applied to the Zr atom, while the def2-TZVP basis set^9^ was used for the remaining atoms. The solvent effect was incorporated using the SMD solvation model.^10^ The three-dimensional molecular structures were shown using CYLView.^11^ The energy profiles were described by these single-point energies, including free energy corrections obtained from the frequency calculations. Weak interaction analyses were performed using the Independent Gradient Model based on Hirshfeld partition (IGMH) method implemented in Multiwfn, and the resulting isosurfaces were visualized with VMD to elucidate the origin of enantioselectivity.^12^

## Optimized transition state structures

**Zr-L16-Me**

**Zr-L29-Me**

Optimized transition state structures (distances in Å) and their relative free energies

**Zr-L35-Me**

Optimized transition state structures (distances in Å) and their relative free energies

**Zr-L16-*^i^*Pr**

Optimized transition state structures (distances in Å) and their relative free energies

**Zr-L29-*^i^*Pr**

Optimized transition state structures (distances in Å) and their relative free energies

## The independent gradient model based on Hirshfeld partition (IGMH)

**Zr-L16**

The independent gradient model based on Hirshfeld partition (IGMH) analysis. **a.** IGMH analysis of the non-covalent interactions in **TS-S** and **TS-R**. **b.** Illustration of the number of major non-covalent interactions in enantiomers of **TS-S** and **TS-R**.

**Zr-L29**

The independent gradient model based on Hirshfeld partition (IGMH) analysis. **a.** IGMH analysis of the non-covalent interactions in **TS-S** and **TS-R**. **b.** Illustration of the number of major non-covalent interactions in enantiomers of **TS-S** and **TS-R**.

**Zr-L35**

The independent gradient model based on Hirshfeld partition (IGMH) analysis. **a.** IGMH analysis of the non-covalent interactions in **TS-S** and **TS-R**. **b.** Illustration of the number of major non-covalent interactions in enantiomers of **TS-S** and **TS-R**.

**Intermediate**

**Table S13.** Optimized Cartesian Coordinates (Å), Gibbs Free Energies (a.u.) and imaginary frequencies (cm^-1^) of **TS-**S and **TS-**R. (**L16**)

Atom	X	Y	Z

**TS-**S, -3698.106564, Imaginary frequency = 346.46*i*

C	0.70562	2.76139	1.12955

H	-0.14554	2.82943	1.82336

C	0.12016	2.78923 -0.29899

H	0.94469	2.79501 -1.02937

C	1.62326	3.9386	1.39105

C	2.66019	4.25106	0.50242

C	1.47948	4.69705	2.55496

C	3.54286	5.29117	0.78075

H	2.78091	3.69482 -0.43149

C	2.36123	5.7414	2.8348

H	0.66501	4.46703	3.24771

C	3.39633	6.03761	1.95044

H	4.34406	5.52566	0.07666

H	2.23557	6.32753	3.74732

H	4.08619	6.85539	2.16775

C	-0.70039	4.05356 -0.49027

C	-0.34742	5.00205 -1.45216

C	-1.83947	4.27493	0.29256

C	-1.11842	6.14993 -1.63356

H	0.54194	4.84745 -2.0687

C	-2.61515	5.41666	0.10966

H	-2.13585	3.55555	1.06236

C	-2.25565	6.35755 -0.85569

H	-0.82931	6.88357 -2.3886

H	-3.50226	5.57351	0.7263

H	-2.86268	7.25343 -0.99977

O	-0.77549 -0.21059	1.6493

O	-1.04761 -1.13904 -1.11942

Zr	0.39299 -0.47592	0.09556

C	0.06959	1.03326	3.49603

C	-0.98357	0.36084	2.83945

C	-0.16562	1.59048	4.75388

C	-2.27189	0.32639	3.40816

C	-1.41568	1.49426	5.36131

H	0.64937	2.10969	5.26579

C	-2.46046	0.87699	4.68077

H	-3.45718	0.83844	5.12665

C	-2.39768	0.5581	-2.05718

C	-3.6061	0.97369 -2.62472

C	-2.21926 -0.7794	-1.66608

C	-4.65085	0.08038 -2.8399

H	-3.70571	2.02464 -2.90185

C	-3.2633	-1.71639 -1.85923

C	-4.43787 -1.24799 -2.44755

H	-5.25082 -1.95569 -2.60124

C	1.43811	1.14796	2.86353

H	1.98723	0.2011	2.94703

H	2.02244	1.91389	3.40008

C	-1.27767	1.5525	-1.91625

H	-1.66727	2.55421 -2.14583

H	-0.48082	1.33476 -2.63282

N	-0.66454	1.55931 -0.56762

H	-1.4469	1.53231	0.09258

N	1.37358	1.4751	1.42643

H	2.35062	1.5271	1.07064

C	2.6482	-2.84962 -0.0892

C	3.07427 -1.72426	0.64979

O	2.32967 -0.7477	0.96632

O	1.22805 -4.68667	0.23788

C	0.04843 -5.29679	0.77127

H	0.36583 -6.25962	1.18441

H	-0.40367 -4.66657	1.54478

H	-0.67351 -5.4529	-0.0394

C	1.37522 -3.38723	0.31467

O	0.47624 -2.6373	0.77147

C	3.85456 -3.53303 -0.67754

H	3.87955 -3.43291 -1.77546

H	3.88998 -4.61137 -0.45549

C	6.36242 -3.05077 -0.10854

C	7.20964 -2.32026	0.72735

C	6.72863 -1.3561	1.62557

C	5.37041 -1.06787	1.66268

C	4.51832 -1.8001	0.83565

C	4.99821 -2.80595 -0.01724

H	6.76841 -3.81384 -0.77376

H	7.43149 -0.82636	2.26916

H	4.98024 -0.30017	2.33289

C	5.95186 -0.03592 -2.89499

C	4.64598 -0.43368 -3.19228

C	3.54842	0.34074 -2.80203

C	3.82275	1.47988 -1.9953

C	5.11928	1.87043 -1.6863

C	6.19272	1.12184 -2.16674

H	6.7911	-0.63831 -3.24819

H	4.50007 -1.33146 -3.79392

H	2.96785	2.07127 -1.69185

H	5.29906	2.77034 -1.09291

H	7.21723	1.43478 -1.9575

C	2.12213	0.00633 -3.33871

O	1.16997 -0.16418 -2.26052

O	1.59663 -1.62883 -1.49942

H	0.98225 -2.17714 -2.02785

C	2.08373 -1.23726 -4.22234

H	2.56021 -2.09679 -3.73382

H	1.03777 -1.48653 -4.45364

H	2.60765 -1.04972 -5.16952

C	1.57764	1.21634 -4.12032

H	0.63679	0.95688 -4.62491

H	1.39815	2.06982 -3.45415

H	2.31791	1.51624 -4.87469

H	-1.58166	1.92378	6.35056

Cl	8.9183	-2.62563	0.66382

C	-5.99538	0.4845	-3.46062

C	-3.12467 -3.18312 -1.41958

C	-2.87556 -3.25061	0.09829

H	-1.88773 -2.86685	0.37396

H	-3.63252 -2.66526	0.63579

H	-2.95437 -4.2924	0.44892

C	-1.97095 -3.84528 -2.19424

H	-2.17676 -3.84749 -3.27597

H	-1.02528 -3.31504 -2.02196

H	-1.84613 -4.89404 -1.88013

C	-6.05319	1.9844	-3.77469

H	-5.94316	2.59532 -2.86525

H	-5.27099	2.28384 -4.4889

H	-7.02498	2.23391 -4.22653

C	-7.13901	0.15201 -2.48395

H	-8.10891	0.4465	-2.91456

H	-7.18867 -0.92428 -2.26099

H	-7.01711	0.6898	-1.53108

C	-3.43304 -0.19003	2.63054

C	-4.20575 -1.31121	3.07494

C	-3.80916	0.46783	1.47857

C	-3.84484 -2.08673	4.21317

C	-5.364	-1.69305	2.33088

C	-4.94326	0.08091	0.73481

H	-3.2359	1.33663	1.14715

C	-4.59801 -3.1681	4.60054

H	-2.94786 -1.81963	4.7746

C	-6.12524 -2.81429	2.76321

C	-5.71388 -0.97152	1.15763

H	-5.19216	0.62219 -0.17839

C	-5.75548 -3.53452	3.87161

H	-4.30124 -3.75317	5.47365

H	-7.01146 -3.09723	2.18972

H	-6.59361 -1.27828	0.58625

H	-6.3479	-4.39458	4.19085

C	-6.20124 -0.29658 -4.77145

H	-5.39517 -0.07698 -5.48832

H	-6.21334 -1.38336 -4.6001

H	-7.16003 -0.02119 -5.23845

C	-4.39916 -3.98905 -1.71133

H	-4.66389 -3.98502 -2.77955

H	-4.24083 -5.03743 -1.41677

H	-5.25838 -3.61158 -1.13612

**TS-**R, -3698.087619, Imaginary frequency = 368.11*i*

Atom	X	Y	Z

C	1.10721 -1.28093	3.0828

H	1.51379 -0.68615	3.9166

C	2.19365 -1.31334	1.99784

H	1.89283 -2.02766	1.21365

C	0.70992 -2.63289	3.65086

C	1.21658 -3.84147	3.16762

C	-0.21613 -2.67138	4.7036

C	0.80393 -5.05673	3.7159

H	1.94487 -3.85881	2.35724

C	-0.6354	-3.88203	5.246

H	-0.62916 -1.74379	5.1072

C	-0.12522 -5.08282	4.75152

H	1.21126 -5.98935	3.32078

H	-1.36051 -3.88722	6.06206

H	-0.4489	-6.03438	5.17759

C	3.56684 -1.72903	2.48647

C	4.34855 -2.5596	1.67675

C	4.11025 -1.24667	3.68124

C	5.64314 -2.91059	2.05298

H	3.9322	-2.93938	0.7385

C	5.40606 -1.59385	4.05948

H	3.53138 -0.5863	4.33175

C	6.17477 -2.42723	3.24786

H	6.2384	-3.56409	1.41213

H	5.81725 -1.21068	4.99539

H	7.18856 -2.69987	3.5474

O	-1.6751	0.97465	0.91162

O	0.83268	1.94984	0.30179

Zr	0.01668	0.17291	0.21237

C	-1.93684	0.96108	3.26305

C	-2.45209	1.19203	1.96929

C	-2.7596	1.11214	4.37809

C	-3.77773	1.65197	1.81628

C	-4.08125	1.52488	4.23426

H	-2.35224	0.9183	5.37423

C	-4.56857	1.80659	2.9628

H	-5.58913	2.1781	2.84563

C	3.21171	1.98071	0.28841

C	4.39843	2.69542	0.08973

C	1.97717	2.6332	0.12641

C	4.39589	4.03884 -0.26627

H	5.34085	2.16226	0.22831

C	1.9292	4.0182	-0.1741

C	3.14579	4.66698 -0.37634

H	3.13113	5.72802 -0.62058

C	-0.48639	0.60532	3.43441

H	-0.30473	0.36278	4.49251

H	0.15173	1.47665	3.19974

C	3.34328	0.52537	0.69398

H	4.21874	0.43027	1.35452

H	3.52461 -0.11467 -0.1775

N	2.14613	0.03406	1.39223

H	1.98811	0.70713	2.1451

N	-0.07313 -0.52395	2.57064

H	-0.85265 -1.17865	2.52225

C	0.0363	-0.89054 -2.88853

C	1.35171 -0.93705 -2.30515

O	1.63849 -0.3484	-1.24649

O	-1.49869	0.61534 -3.80918

C	-2.41608	1.70757 -3.66515

H	-3.28668	1.37207 -3.08826

H	-1.93527	2.54034 -3.13801

H	-2.70789	1.98834 -4.68052

C	-0.80173	0.28718 -2.76156

O	-0.85807	0.92665 -1.70541

C	0.02393 -1.77037 -4.11976

H	-0.09285 -1.16409 -5.03267

H	-0.79927 -2.50201 -4.11867

C	1.90349 -3.41663 -4.89694

C	3.21255 -3.83883 -4.6546

C	3.99909 -3.30056 -3.62197

C	3.47229 -2.31343 -2.8033

C	2.16614 -1.87999 -3.04889

C	1.38523 -2.41787 -4.08226

H	1.32135 -3.8599	-5.70575

H	5.01476 -3.6685	-3.47521

H	4.06243 -1.88595 -1.99

C	-4.42993 -1.19698 -1.79903

C	-3.60575 -1.45736 -0.70822

C	-3.0518	-2.73363 -0.5107

C	-3.35753 -3.73722 -1.43952

C	-4.19198 -3.47809 -2.52999

C	-4.72721 -2.20705 -2.71509

H	-4.85521 -0.20008 -1.92625

H	-3.36303 -0.64593 -0.01848

H	-2.9442	-4.73931 -1.32441

H	-4.42042 -4.27941 -3.23589

H	-5.37871 -2.00122 -3.56674

C	-2.06757 -2.9582	0.65482

O	-0.98536 -2.04838	0.53307

O	-0.61187 -1.70053 -1.23682

H	-1.43308 -2.12984 -1.52707

C	-1.49075 -4.37295	0.67264

H	-2.26904 -5.13335	0.83258

H	-0.96007 -4.59633 -0.26415

H	-0.76929 -4.44208	1.49625

C	-2.78883 -2.67925	1.98293

H	-3.0381	-1.61199	2.0844

H	-3.72587 -3.25239	2.03723

H	-2.15651 -2.99057	2.82739

H	-4.72063	1.64989	5.10937

Cl	3.89661 -5.07078 -5.66325

C	5.67851	4.84973 -0.49769

C	0.59092	4.7778	-0.25499

C	6.93402	3.9707	-0.43368

H	7.05282	3.49417	0.55132

H	6.91807	3.18122 -1.20106

H	7.82849	4.58753 -0.60782

C	5.79251	5.93926	0.58461

H	5.9217	5.48848	1.58067

H	6.66008	6.58895	0.38754

H	4.89645	6.57677	0.61736

C	-0.20101	4.60106	1.05691

H	-0.49659	3.55635	1.21522

H	0.39801	4.9302	1.92054

H	-1.11349	5.21795	1.02984

C	-0.24197	4.26021 -1.44244

H	0.32514	4.34874 -2.38281

H	-0.53222	3.21193 -1.3049

H	-1.15965	4.86158 -1.54543

C	-4.31362	2.08893	0.49731

C	-5.55127	1.57895 -0.01891

C	-3.6494	3.08021 -0.19722

C	-6.25574	0.49913	0.58749

C	-6.08851	2.14807 -1.21445

C	-4.1901	3.6474	-1.37211

H	-2.70301	3.45663	0.19338

C	-7.42403	0.02054	0.04761

H	-5.8435	0.02923	1.48133

C	-7.307	1.63544 -1.74246

C	-5.39071	3.20091 -1.86537

H	-3.65306	4.45389 -1.87632

C	-7.96165	0.5973	-1.12814

H	-7.93898 -0.81663	0.52323

H	-7.71146	2.08317 -2.65375

H	-5.82332	3.64314 -2.76616

H	-8.89368	0.21083 -1.5456

C	5.62334	5.50953 -1.88841

H	5.46649	4.75532 -2.6749

H	4.81187	6.2487	-1.96207

H	6.5679	6.03435 -2.10078

C	0.80739	6.285	-0.46026

H	1.40205	6.73104	0.35146

H	1.30263	6.50817 -1.41749

H	-0.16879	6.79238 -0.47153

**Table S14.** Optimized Cartesian Coordinates (Å), Gibbs Free Energies (a.u.) and imaginary frequencies (cm^-1^) of **TS-**S and **TS-**R. (**L29**)

Atom	X	Y	Z

**TS-**S, -3705.10, Imaginary frequency = 348.34*i*

O	0.72472	0.37953 -0.73225

O	0.02955 -2.51108 -0.40764

Zr	-0.7856	-0.7035	-0.03654

C	0.80496	2.59508	0.06841

C	1.45637	1.4903	-0.51431

C	1.50564	3.76732	0.31731

C	2.8246	1.55895 -0.83608

C	2.85417	3.88394 -0.02714

H	0.98929	4.60895	0.77387

C	3.47605	2.77751 -0.60102

H	4.51997	2.84734 -0.87617

C	1.48661 -3.26671	1.29162

C	2.58856 -4.00814	1.69995

C	1.03058 -3.33361 -0.03643

C	3.25534 -4.86107	0.81901

H	2.9235	-3.91172	2.73034

C	1.63888 -4.23	-0.93915

C	2.73978 -4.96052 -0.47422

H	3.22613 -5.63414 -1.16581

C	-0.63379	2.45675	0.45924

H	-1.28344	2.32878 -0.409

H	-0.96246	3.35883	0.98622

C	0.74495 -2.42783	2.27982

H	1.24385 -2.50589	3.24861

H	-0.2786	-2.79049	2.39841

N	0.63424 -1.00092	1.87587

H	1.54948 -0.70517	1.52393

N	-0.84374	1.25649	1.33148

H	-1.81784	1.28455	1.64146

C	-3.5455	-0.93106 -2.05933

C	-3.56542	0.18503 -1.16288

O	-2.5927	0.58421 -0.45569

O	-2.48162 -1.65455 -3.9908

C	-1.30794 -1.80082 -4.80664

H	-0.70142 -0.89443 -4.76636

H	-0.72263 -2.65046 -4.46035

H	-1.6823	-1.97524 -5.81419

C	-2.32226 -1.18824 -2.76216

O	-1.18922 -0.93259 -2.29364

C	-4.93167 -1.16104 -2.6055

H	-5.32981 -2.14501 -2.32543

H	-4.9554	-1.11173 -3.70046

C	-7.07072	0.26731 -2.11764

C	-7.5411	1.41545 -1.47427

C	-6.71198	2.24615 -0.70909

C	-5.37554	1.91068 -0.54479

C	-4.89793	0.7629	-1.1761

C	-5.72585 -0.03905 -1.98021

H	-7.74089 -0.33995 -2.71679

H	-7.12756	3.13089 -0.23971

H	-4.71789	2.51885	0.06602

C	-6.29421	0.03236	1.90936

C	-5.47644 -1.08943	1.78506

C	-4.17033 -1.08131	2.27955

C	-3.70067	0.09104	2.88414

C	-4.5074	1.21878	3.00122

C	-5.81582	1.18807	2.51953

H	-7.30567	0.00401	1.51572

H	-5.87634 -1.97753	1.30942

H	-2.70191	0.11292	3.3081

H	-4.12436	2.11116	3.48896

H	-6.45577	2.06	2.61738

C	-3.27267 -2.31005	2.22571

O	-2.13223 -2.0783	1.3808

O	-2.78375 -1.94714 -0.21808

H	-2.52166 -2.84433 -0.51765

C	-3.97619 -3.57784	1.74631

H	-4.43039 -3.44412	0.76307

H	-3.24559 -4.39006	1.68726

H	-4.75796 -3.86949	2.45328

C	-2.67922 -2.60849	3.61341

H	-2.06023 -3.50876	3.5774

H	-2.07869 -1.78416	4.00036

H	-3.50437 -2.7777	4.31189

Cl	-9.21929	1.83613 -1.63766

C	3.56783	0.38793 -1.49567

C	3.32329 -0.93854 -0.74645

H	3.53879 -0.84602	0.32344

H	2.29451 -1.26718 -0.8653

H	3.97778 -1.72102 -1.14251

C	3.01352	0.24787 -2.92335

H	3.49953 -0.57726 -3.45524

H	1.94206	0.03943 -2.87525

H	3.15631	1.16929 -3.49729

C	3.60322	5.17503	0.30286

C	3.82263	5.19864	1.82542

H	2.87263	5.21632	2.37061

H	4.36803	4.29789	2.12549

H	4.41573	6.06737	2.13193

C	4.98203	5.24228 -0.37569

H	5.44627	6.20932 -0.15835

H	5.64996	4.45656 -0.00537

H	4.90221	5.14393 -1.46289

C	4.49193 -5.62933	1.28334

C	4.11543 -6.54368	2.46174

H	3.72016 -5.97132	3.3079

H	3.35302 -7.2711	2.16097

H	4.99562 -7.09517	2.81319

C	5.08911 -6.49669	0.17086

H	5.97466 -7.01919	0.54875

H	4.37986 -7.25603 -0.17772

H	5.40095 -5.89381 -0.6896

C	5.56313 -4.62196	1.73765

H	5.8407	-3.95282	0.91546

H	5.20829 -4.00383	2.56944

H	6.4649	-5.14826	2.07261

C	1.17475 -4.36615 -2.39068

C	1.84748 -5.54541 -3.10581

H	1.66902 -6.49421 -2.58706

H	1.43435 -5.63343 -4.11673

H	2.92869 -5.40425 -3.2092

C	1.55692 -3.08069 -3.14041

H	1.01863 -2.21491 -2.74936

H	2.63087 -2.89365 -3.04347

H	1.33583 -3.18024 -4.21017

C	-0.34437 -4.60246 -2.45487

H	-0.61909 -5.49907 -1.88701

H	-0.8907	-3.75076 -2.04675

H	-0.65963 -4.75794 -3.49372

C	1.32682 -0.09206	4.14109

C	0.89362	0.85449	5.26253

C	0.62351	2.26269	4.73881

C	-0.39956	2.23286	3.60458

C	0.05572	1.27998	2.50241

C	0.28143 -0.12965	3.02297

H	0.26801	2.911	5.54695

H	-0.01774	0.4587	5.73331

H	1.66384	0.87542	6.04096

H	2.28315	0.24354	3.71387

H	1.49554 -1.08975	4.5552

H	-1.37516	1.90767	3.99417

H	-0.53879	3.23761	3.19112

H	1.02257	1.63591	2.12644

H	-0.65462 -0.53165	3.42648

H	1.55872	2.70281	4.36643

C	5.07727	0.63848 -1.4845

C	5.82121	0.83471 -2.64948

C	5.76042	0.67278 -0.25885

C	7.19986	1.05336 -2.59719

H	5.33148	0.82099 -3.61702

C	7.13145	0.89477 -0.1995

H	5.20431	0.54566	0.66599

C	7.86152	1.085	-1.37412

H	7.75346	1.20128 -3.52069

H	7.63291	0.91971	0.76455

H	8.9334	1.25746 -1.3325

C	2.77827	6.36549 -0.18971

C	2.4743	7.46581	0.61435

C	2.32624	6.37142 -1.51701

C	1.74201	8.54279	0.1095

H	2.8062	7.49608	1.64706

C	1.59968	7.44235 -2.02523

H	2.53931	5.51538 -2.15269

C	1.30259	8.53683 -1.21077

H	1.51749	9.38761	0.75558

H	1.26138	7.42327 -3.05806

H	0.73344	9.3748	-1.60393

**TS-**R, -3705.07, Imaginary frequency = 418.02*i*

Atom	X	Y	Z

O	-0.61666	0.87611 -0.04312

O	2.02768	1.83796 -0.65165

Zr	1.21339	0.161	-0.07814

C	-1.76895	0.58912	1.96733

C	-1.81236	0.83248	0.58285

C	-2.94117	0.52186	2.71239

C	-3.03921	1.05242 -0.06454

C	-4.17912	0.72507	2.10225

H	-2.87506	0.33148	3.77979

C	-4.18564	0.995	0.73561

H	-5.14297	1.19697	0.26621

C	3.97413	2.50881	0.50764

C	4.89188	3.49622	0.84947

C	2.90925	2.81996 -0.35311

C	4.78913	4.79258	0.33885

H	5.707	3.23736	1.52143

C	2.75425	4.11353 -0.88116

C	3.71661	5.06181 -0.51539

H	3.61709	6.06151 -0.91478

C	-0.43595	0.51422	2.64509

H	-0.59029	0.29386	3.70856

H	0.07412	1.48959	2.59231

C	4.13134	1.11053	1.03386

H	4.91979	1.10521	1.79337

H	4.41811	0.40593	0.24729

N	2.86065	0.60942	1.61726

H	2.51227	1.34734	2.23499

N	0.49632 -0.50478	2.07847

H	-0.08433 -1.3405	1.86645

C	1.62411 -2.56087 -1.91793

C	2.74902 -2.30252 -1.05207

O	2.99958 -1.1795	-0.51664

O	0.84909 -1.74986 -3.98048

C	0.3749	-0.66314 -4.80564

H	0.13825 -1.12089 -5.76383

H	-0.50763 -0.21147 -4.35257

H	1.16181	0.08621 -4.911

C	1.11662 -1.46896 -2.73176

O	0.98152 -0.32253 -2.25993

C	1.60643 -4.01164 -2.3128

H	1.80221 -4.13444 -3.38525

H	0.6271	-4.46605 -2.11611

C	3.09775 -5.91251 -1.30611

C	4.17503 -6.18328 -0.45525

C	4.86711 -5.16934	0.22657

C	4.47669 -3.848	0.06954

C	3.4017	-3.56885 -0.77953

C	2.72061 -4.59011 -1.47273

H	2.58793 -6.72372 -1.81529

H	5.69953 -5.43416	0.86927

H	4.99667 -3.04615	0.58493

C	-2.28343 -2.5174	-3.16511

C	-2.11352 -2.38698 -1.7924

C	-1.99084 -3.51435 -0.97426

C	-2.04523 -4.77846 -1.56254

C	-2.19946 -4.91267 -2.94581

C	-2.31662 -3.78422 -3.75184

H	-2.4045	-1.62892 -3.77924

H	-2.05444 -1.41027 -1.32438

H	-1.96706 -5.67365 -0.95278

H	-2.23632 -5.9047	-3.38806

H	-2.44667 -3.88945 -4.82523

C	-1.80873 -3.29676	0.52739

O	-1.06528 -2.13906	0.75722

O	0.41628 -2.23069 -0.47725

H	0.72082 -2.93418	0.12108

C	-1.15801 -4.49464	1.22912

H	-1.78952 -5.38806	1.21529

H	-0.20114 -4.76481	0.76096

H	-0.97202 -4.23242	2.27517

C	-3.18931 -2.99552	1.14971

H	-3.61112 -2.10121	0.68509

H	-3.87018 -3.83892	0.99125

H	-3.08346 -2.81255	2.22386

Cl	4.67195 -7.82374 -0.23267

C	-3.25841	1.45388 -1.53055

C	-1.99588	1.40785 -2.40786

H	-1.22901	2.10259 -2.06708

H	-1.55841	0.40602 -2.42447

H	-2.27685	1.68837 -3.42959

C	-4.28902	0.4904	-2.15312

H	-4.36585	0.65204 -3.23355

H	-3.984	-0.54619 -1.99424

H	-5.28815	0.61049 -1.72629

C	-5.50025	0.77741	2.87261

C	-5.82719	2.26311	3.10169

H	-5.91125	2.80914	2.15667

H	-6.76101	2.38203	3.66264

H	-5.02252	2.72812	3.68051

C	-5.40412	0.09416	4.24644

H	-5.06253 -0.94271	4.16514

H	-4.72028	0.63213	4.91272

H	-6.3902	0.08619	4.72061

C	1.44526 -2.22047	3.64937

C	2.55897 -2.44383	4.6753

C	3.94217 -2.17315	4.07968

C	4.03805 -0.78419	3.43585

C	2.93825 -0.65143	2.38689

C	1.57338 -0.83025	3.04103

H	4.7125	-2.27834	4.85094

H	2.39399 -1.77395	5.53044

H	2.50916 -3.46572	5.06629

H	1.51067 -2.96959	2.84683

H	0.45831 -2.33493	4.11314

H	3.92598	0.0005	4.19792

H	5.02701 -0.65677	2.98373

H	3.08955 -1.44537	1.64636

H	1.51409 -0.09464	3.85741

H	4.16048 -2.93205	3.31423

C	5.82328	5.84949	0.72753

C	1.56917	4.47075 -1.78075

C	5.54309	7.20551	0.07203

H	5.56817	7.14184 -1.02166

H	4.57032	7.61055	0.37318

H	6.30915	7.92579	0.37811

C	5.8049	6.03779	2.25456

H	4.81794	6.37116	2.5943

H	6.04718	5.10862	2.78182

H	6.54217	6.79164	2.55406

C	1.54559	3.56405 -3.02554

H	2.46318	3.69257 -3.61146

H	1.45098	2.51033 -2.75753

H	0.69537	3.83603 -3.6624

C	1.63549	5.92328 -2.26739

H	1.59846	6.63766 -1.43731

H	2.53999	6.11894 -2.85455

H	0.77254	6.12487 -2.91084

C	-6.57266	0.06365	2.04674

C	-6.39157 -1.28966	1.73119

C	-7.71713	0.70257	1.56704

C	-7.31777 -1.98166	0.96067

H	-5.50261 -1.80221	2.08646

C	-8.65156	0.01209	0.79183

H	-7.89174	1.75031	1.78733

C	-8.45633 -1.33013	0.48393

H	-7.15121 -3.03029	0.72817

H	-9.53379	0.5323	0.42853

H	-9.18259 -1.86604 -0.12072

C	-3.76078	2.90468 -1.544

C	-4.73558	3.34501 -2.44282

C	-3.19289	3.84413 -0.67445

C	-5.13145	4.68253 -2.47274

H	-5.20236	2.64597 -3.12907

C	-3.58122	5.17966 -0.70363

H	-2.44398	3.52082	0.04248

C	-4.55603	5.60621 -1.60501

H	-5.89643	4.9989	-3.17685

H	-3.1253	5.88743 -0.01629

H	-4.86666	6.64703 -1.62641

C	7.21935	5.37755	0.28497

H	7.49821	4.43077	0.76003

H	7.25574	5.23266 -0.80035

H	7.97657	6.12215	0.55662

C	0.26473	4.30538 -0.98143

H	-0.59706	4.57341 -1.60225

H	0.12571	3.27808 -0.63786

H	0.26424	4.96527 -0.1056

## References

[1]	(a) G. Guo, Y. Zhang, Z. Huang, W. Liu, X. Lu, Z. Liu, X.-Q. Xiao, Y. Bai, X. Shao. Direct trideuteromethylselenation with a shelf-stable reagent Se-methyl-d3 selenosulfonate. *Org. Chem. Front.* **2025**, *12*, 2592-2599; (b) M. A. Pasha, J. Jang, Y. Bae, S. Shin. N‐Insertion of Diazonium Salts Into Ketone Derivatives. *Angew. Chem. Int. Ed.* **2025**, *64*.

[2]	(a) S. V. Kochetkov, A. S. Kucherenko, S. G. Zlotin. Asymmetric synthesis of warfarin and its analogs catalyzed by C2-symmetric squaramide-based primary diamines. *Org. Biomol. Chem.* **2018**, *16*, 6423-6429; (b) H. b. Liu, M. Wang, Y. Wang, Q. Gu, L. c. Sun. Synthesis of 3‐Aryl‐5‐t‐butylsalicylaldehydes and their Chiral Schiff Base Compounds. *Synth. Commun.* **2007**, *37*, 3815-3826; (c) F. Yang, J. Zhao, X. Tang, G. Zhou, W. Song, Q. Meng. Enantioselective α-Hydroxylation by Modified Salen-Zirconium(IV)-Catalyzed Oxidation of β-Keto Esters. *Org. Lett.* **2017**, *19*, 448-451.

[3]	(a) C. Ma, K. Tang, Y. Wu, Y. Wang, Q. Liu, J. Zhao, J. Li, L. Duan, X. Tian, Q. Meng. Highly Enantioselective Hydroxylation of 1-Indanone-Derived β-Ketoesters Catalyzed by a Newly Designed C1-Symmetric Salan-Zr Complex. *J. Org. Chem.* **2026**, *91*, 955-965; (b) C. Ma, K. Tang, J. Zhao, Y. Wang, Y. Wu, G. Zhao, H. Sun, Q. Liu, Q. Meng. A robust Zr/Hf catalytic system for efficient and highly enantioselective α-hydroxylation of β-keto carbonyls and amides: mechanistic and DFT studies. *J. Catal.* **2026**, *454*.

[4]	J. Chen, H. Y. Gu, X. Y. Zhu, W. Nam, B. Wang. Zirconium-Salan Catalyzed Enantioselective α-Hydroxylation of β-Keto Esters. *Adv Synth Catal* **2020**, *362*, 2976-2983.

[5]	(a) A. D. Becke. Density-functional thermochemistry. III. The role of exact exchange. *J. Chem. Phys.* **1993**, *98*, 5648-5652; (b) J. P. Perdew, K. Burke, Y. Wang. Generalized gradient approximation for the exchange-correlation hole of a many-electron system. *J. Phys. Chem. B* **1996**, *54*, 16533.

[6]	M. J. Frisch, G. W. Trucks, H. B. Schlegel, G. E. Scuseria, M. A. Robb, J. R. Cheeseman, G. Scalmani, V. Barone, G. A. Petersson, H. Nakatsuji, X. Li, M. Caricato, A. V. Marenich, J. Bloino, B. G. Janesko, R. Gomperts, B. Mennucci, H. P. Hratchian, J. V. Ortiz, A. F. Izmaylov, J. L. Sonnenberg, Williams, F. Ding, F. Lipparini, F. Egidi, J. Goings, B. Peng, A. Petrone, T. Henderson, D. Ranasinghe, V. G. Zakrzewski, J. Gao, N. Rega, G. Zheng, W. Liang, M. Hada, M. Ehara, K. Toyota, R. Fukuda, J. Hasegawa, M. Ishida, T. Nakajima, Y. Honda, O. Kitao, H. Nakai, T. Vreven, K. Throssell, J. A. Montgomery Jr., J. E. Peralta, F. Ogliaro, M. J. Bearpark, J. J. Heyd, E. N. Brothers, K. N. Kudin, V. N. Staroverov, T. A. Keith, R. Kobayashi, J. Normand, K. Raghavachari, A. P. Rendell, J. C. Burant, S. S. Iyengar, J. Tomasi, M. Cossi, J. M. Millam, M. Klene, C. Adamo, R. Cammi, J. W. Ochterski, R. L. Martin, K. Morokuma, O. Farkas, J. B. Foresman, D. J. Fox, Wallingford, CT, **2016**.

[7]	P. C. Hariharan, J. Pople. The Influence of Polarization Functions on Molecular Orbital Hydrogenation Energies. *J. Theor. Chim. Acta* **1973**, *28*, 213-222.

[8]	S. Grimme, J. Antony, S. Ehrlich, H. Krieg. A consistent and accurate *ab* initio parametrization of density functional dispersion correction (DFT-D) for the 94 elements H-Pu. *J. Chem. Phys.* **2010**, *132*, 154104.

[9]	D. Andrae, U. Haeussermann, M. Dolg, H. Stoll, H. Preuss. Energy adjusted ab initio pseudopotentials for the second and third row transition elements. *Theor. Chem. Acc.* **1990**, *77*, 123-141.

[10]	A. V. Marenich, C. J. Cramer, D. G. Truhlar. Universal Solvation Model Based on Solute Electron Density and on a Continuum Model of the Solvent Defined by the Bulk Dielectric Constant and Atomic Surface Tensions. *J. Phys. Chem. B* **2009**, *113*, 6378-6396.

[11]	Legault, C.YCYLview, 1.0b. Université de Sherbrooke. *Université de Sherbrooke,* **2009**, (<http://www.cylview.org>).

[12]	(a) T. Lu, F. Chen. Multiwfn: A multifunctional wavefunction analyzer. *J. Comput. Chem.* **2012**, *33*, 580-592; (b) T. Lu, Q. Chen. Independent gradient model based on Hirshfeld partition: A new method for visual study of interactions in chemical systems. *J. Comput. Chem.* **2022**, *43*, 539-555; (c) W.-F. Zheng, J. Chen, X. Qi, Z. Huang. Modular and diverse synthesis of amino acids via asymmetric decarboxylative protonation of aminomalonic acids. *Nat. Chem.* **2023**, *15*, 1672-1682; (d) T. Lu. A comprehensive electron wavefunction analysis toolbox for chemists, Multiwfn. *J. Chem. Phys.* **2024**, *161*, 082503; (e) T. Lu. Visualization Analysis of Covalent and Noncovalent Interactions in Real Space. *Angew. Chem. Int. Ed.* **2025**, e202504895.

## NMR spectra

## NMR spectra of chiral ligands

**2-((((1S,2S)-2-amino-1,2-diphenylethyl)amino)methyl)-4,6-di-tert-butylphenol (M3)**

^13^C{^1^H} NMR (101 MHz, CDCl_3_) spectra of compound M3

**2-((((1S,2S)-2-aminocyclohexyl)amino)methyl)-4,6-di-tert-butylphenol (M4)**

^1^H NMR (400 MHz, CDCl_3_) spectra of compound M4

^13^C{^1^H} NMR (101 MHz, CDCl_3_) spectra of compound M4

**2-((((1S,2S)-2-aminocyclohexyl)amino)methyl)-6-(tert-butyl)phenol (M5)**

^1^H NMR (500 MHz, CDCl_3_) spectra of compound M5

^13^C{^1^H} NMR (126 MHz, CDCl_3_) spectra of compound M5

**2,4-di-tert-butyl-6-((((1S,2S)-2-((3-(tert-butyl)-2-hydroxybenzyl)amino)-1,2-diphenylethyl)amino)methyl)phenol (L1)**

^1^H NMR (400 MHz, CDCl_3_) spectra of compound L1

^13^C{^1^H} NMR (101 MHz, CDCl_3_) spectra of compound L1

**2,4-di-tert-butyl-6-((((1S,2S)-2-((3-(tert-butyl)-2-hydroxy-5-methoxybenzyl)amino)-1,2-diphenylethyl)amino)methyl)phenol (L2)**

^1^H NMR (400 MHz, CDCl_3_) spectra of compound L2

^13^C{^1^H} NMR (101 MHz, CDCl_3_) spectra of compound L2

**2-((((1S,2S)-2-((3-bromo-2-hydroxybenzyl)amino)-1,2-diphenylethyl)amino)methyl)-4,6-di-tert-butylphenol (L3)**

^1^H NMR (400 MHz, CDCl_3_) spectra of compound L3

^13^C{^1^H} NMR (101 MHz, CDCl_3_) spectra of compound L3

**2,4-di-tert-butyl-6-((((1S,2S)-2-((3-(tert-butyl)-5-((dimethylamino)methyl)-2-hydroxybenzyl)amino)-1,2-diphenylethyl)amino)methyl)phenol (L4)**

^1^H NMR (400 MHz, CDCl_3_) spectra of compound L4

^13^C{^1^H} NMR (101 MHz, CDCl_3_) spectra of compound L4

**2,4-di-tert-butyl-6-((((1S,2S)-2-((3-(tert-butyl)-5-((diethylamino)methyl)-2-hydroxybenzyl)amino)-1,2-diphenylethyl)amino)methyl)phenol (L5)**

^1^H NMR (400 MHz, CDCl_3_) spectra of compound L5

^13^C{^1^H} NMR (101 MHz, CDCl_3_) spectra of compound L5

**2,4-di-tert-butyl-6-((((1S,2S)-2-((3-(tert-butyl)-2-hydroxy-5-(morpholinomethyl)benzyl)amino)-1,2-diphenylethyl)amino)methyl)phenol (L6)**

^1^H NMR (400 MHz, CDCl_3_) spectra of compound L6

^13^C{^1^H} NMR (101 MHz, CDCl_3_) spectra of compound L6

**2,4-di-tert-butyl-6-((((1S,2S)-2-((5-(tert-butyl)-2-hydroxy-3-(morpholinomethyl)benzyl)amino)-1,2-diphenylethyl)amino)methyl)phenol (L7)**

^1^H NMR (400 MHz, CDCl_3_) spectra of compound L7

^13^C{^1^H} NMR (101 MHz, CDCl_3_) spectra of compound L7

**2,4-di-tert-butyl-6-((((1S,2S)-2-((3-(tert-butyl)-5-((diphenylamino)methyl)-2-hydroxybenzyl)amino)-1,2-diphenylethyl)amino)methyl)phenol (L8)**

^1^H NMR (400 MHz, CDCl_3_) spectra of compound L8

^13^C{^1^H} NMR (101 MHz, CDCl_3_) spectra of compound L8

**3-((((1S,2S)-2-((3,5-di-tert-butyl-2-hydroxybenzyl)amino)-1,2-diphenylethyl)amino)methyl)-[1,1'-biphenyl]-2-ol (L9)**

^1^H NMR (500 MHz, CDCl_3_) spectra of compound L9

^13^C{^1^H} NMR (126 MHz, CDCl_3_) spectra of compound L9

**3-((((1S,2S)-2-((3,5-di-tert-butyl-2-hydroxybenzyl)amino)-1,2-diphenylethyl)amino)methyl)-4'-methoxy-[1,1'-biphenyl]-2-ol (L10)**

^1^H NMR (400 MHz, CDCl_3_) spectra of compound L10

^13^C{^1^H} NMR (101 MHz, CDCl_3_) spectra of compound L10

**3-((((1S,2S)-2-((3,5-di-tert-butyl-2-hydroxybenzyl)amino)-1,2-diphenylethyl)amino)methyl)-3',4'-dimethoxy-[1,1'-biphenyl]-2-ol (L11)**

^1^H NMR (400 MHz, CDCl_3_) spectra of compound L11

^13^C{^1^H} NMR (101 MHz, CDCl_3_) spectra of compound L11

**3-((((1S,2S)-2-((3,5-di-tert-butyl-2-hydroxybenzyl)amino)-1,2-diphenylethyl)amino)methyl)-2',5,6'-trimethoxy-[1,1'-biphenyl]-2-ol (L12)**

^1^H NMR (400 MHz, CDCl_3_) spectra of compound L12

^13^C{^1^H} NMR (101 MHz, CDCl_3_) spectra of compound L12

**3-((((1S,2S)-2-((3,5-di-tert-butyl-2-hydroxybenzyl)amino)-1,2-diphenylethyl)amino)methyl)-3',5'-dimethoxy-[1,1'-biphenyl]-2-ol (L13)**

^1^H NMR (400 MHz, CDCl_3_) spectra of compound L13

^13^C{^1^H} NMR (101 MHz, CDCl_3_) spectra of compound L13

**3',5'-di-tert-butyl-3-((((1S,2S)-2-((3,5-di-tert-butyl-2-hydroxybenzyl)amino)-1,2-diphenylethyl)amino)methyl)-[1,1'-biphenyl]-2-ol (L14)**

^1^H NMR (400 MHz, CDCl_3_) spectra of compound L14

^13^C{^1^H} NMR (101 MHz, CDCl_3_) spectra of compound L14

**2,4-di-tert-butyl-6-((((1S,2S)-2-((3-(dibenzo[b,d]furan-4-yl)-2-hydroxybenzyl)amino)-1,2-diphenylethyl)amino)methyl)phenol (L15)**

^1^H NMR (400 MHz, CDCl_3_) spectra of compound L15

^13^C{^1^H} NMR (101 MHz, CDCl_3_) spectra of compound L15

**2,4-di-tert-butyl-6-((((1S,2S)-2-((2-hydroxy-3-(naphthalen-1-yl)benzyl)amino)-1,2-diphenylethyl)amino)methyl)phenol (L16)**

^1^H NMR (400 MHz, CDCl_3_) spectra of compound L16

^13^C{^1^H} NMR (101 MHz, CDCl_3_) spectra of compound L16

**2,4-di-tert-butyl-6-((((1S,2S)-2-((2-hydroxy-5-methoxy-3-(naphthalen-1-yl)benzyl)amino)-1,2-diphenylethyl)amino)methyl)phenol (L17)**

^1^H NMR (400 MHz, CDCl_3_) spectra of compound L17

^13^C{^1^H} NMR (101 MHz, CDCl_3_) spectra of compound L17

**2,4-di-tert-butyl-6-((((1S,2S)-2-((5-(tert-butyl)-2-hydroxy-3-(naphthalen-1-yl)benzyl)amino)-1,2-diphenylethyl)amino)methyl)phenol (L18)**

^1^H NMR (400 MHz, CDCl_3_) spectra of compound L18

^13^C{^1^H} NMR (101 MHz, CDCl_3_) spectra of compound L18

**2,4-di-tert-butyl-6-((((1S,2S)-2-((2-hydroxy-3,5-di(naphthalen-1-yl)benzyl)amino)-1,2-diphenylethyl)amino)methyl)phenol (L19)**

^1^H NMR (400 MHz, CDCl_3_) spectra of compound L19

^13^C{^1^H} NMR (101 MHz, CDCl_3_) spectra of compound L19

**2,4-di-tert-butyl-6-((((1S,2S)-2-((2-hydroxy-3-(naphthalen-2-yl)benzyl)amino)-1,2-diphenylethyl)amino)methyl)phenol (L20)**

^1^H NMR (400 MHz, CDCl_3_) spectra of compound L20

^13^C{^1^H} NMR (101 MHz, CDCl_3_) spectra of compound L20

**2,4-di-tert-butyl-6-((((1S,2S)-2-((2-hydroxy-3-(phenanthren-9-yl)benzyl)amino)-1,2-diphenylethyl)amino)methyl)phenol (L21)**

^1^H NMR (400 MHz, CDCl_3_) spectra of compound L21

^13^C{^1^H} NMR (101 MHz, CDCl_3_) spectra of compound L21

**2,4-di-tert-butyl-6-((((1S,2S)-2-((2-hydroxy-5-methoxy-3-(phenanthren-9-yl)benzyl)amino)-1,2-diphenylethyl)amino)methyl)phenol (L22)**

^1^H NMR (400 MHz, CDCl_3_) spectra of compound L22

^13^C{^1^H} NMR (101 MHz, CDCl_3_) spectra of compound L22

**2,4-di-tert-butyl-6-((((1S,2S)-2-((5-(tert-butyl)-2-hydroxy-3-(phenanthren-9-yl)benzyl)amino)-1,2-diphenylethyl)amino)methyl)phenol (L23)**

^1^H NMR (400 MHz, CDCl_3_) spectra of compound L23

^13^C{^1^H} NMR (101 MHz, CDCl_3_) spectra of compoundL23

**2,4-di-tert-butyl-6-((((1S,2S)-2-((2-hydroxy-3-(pyren-1-yl)benzyl)amino)-1,2-diphenylethyl)amino)methyl)phenol (L24)**

^1^H NMR (400 MHz, CDCl_3_) spectra of compound L24

^13^C{^1^H} NMR (101 MHz, CDCl_3_) spectra of compound L24

**2-((((1S,2S)-2-((3-(anthracen-9-yl)-2-hydroxybenzyl)amino)-1,2-diphenylethyl)amino)methyl)-4,6-di-tert-butylphenol (L25)**

^1^H NMR (400 MHz, CDCl_3_) spectra of compound L25

^13^C{^1^H} NMR (101 MHz, CDCl_3_) spectra of compound L25

**4-(butoxymethyl)-2-(tert-butyl)-6-((((1S,2S)-2-((3,5-di-tert-butyl-2-hydroxybenzyl)amino)-1,2-diphenylethyl)amino)methyl)phenol (L26)**

^1^H NMR (500 MHz, CDCl_3_) spectra of compound L26

^13^C{^1^H} NMR (126 MHz, CDCl_3_) spectra of compound L26

**2,4-di-tert-butyl-6-((((1S,2S)-2-((2-hydroxy-3-(2-phenylpropan-2-yl)benzyl)amino)-1,2-diphenylethyl)amino)methyl)phenol (L27)**

^1^H NMR (400 MHz, CDCl_3_) spectra of compound L27

^13^C{^1^H} NMR (101 MHz, CDCl_3_) spectra of compound L27

**2,4-di-tert-butyl-6-((((1S,2S)-2-((2-hydroxy-3-tritylbenzyl)amino)-1,2-diphenylethyl)amino)methyl)phenol (L28)**

^1^H NMR (500 MHz, CDCl_3_) spectra of compound L28

^13^C{^1^H} NMR (126 MHz, CDCl_3_) spectra of compound L28

**2,4-di-tert-butyl-6-((((1S,2S)-2-((2-hydroxy-3,5-bis(2-phenylpropan-2-yl)benzyl)amino)cyclohexyl)amino)methyl)phenol (L29)**

^1^H NMR (400 MHz, CDCl_3_) spectra of compound L29

^13^C{^1^H} NMR (101 MHz, CDCl_3_) spectra of compound L29

**2,4-di-tert-butyl-6-((((1S,2S)-2-((3-(tert-butyl)-2-hydroxybenzyl)amino)cyclohexyl)amino)methyl)phenol (L30)**

^1^H NMR (400 MHz, CDCl_3_) spectra of compound L30

^13^C{^1^H} NMR (101 MHz, CDCl_3_) spectra of compound L30

**2-((3S,5S,7S)-adamantan-1-yl)-4-(tert-butyl)-6-((((1S,2S)-2-((3,5-di-tert-butyl-2-hydroxybenzyl)amino)cyclohexyl)amino)methyl)phenol (L31)**

^1^H NMR (400 MHz, CDCl_3_) spectra of compound L31

^13^C{^1^H} NMR (101 MHz, CDCl_3_) spectra of compound L31

**2,4-di-tert-butyl-6-((((1S,2S)-2-((5-(tert-butyl)-2-hydroxy-3-tritylbenzyl)amino)cyclohexyl)amino)methyl)phenol (L32)**

^1^H NMR (400 MHz, CDCl_3_) spectra of compound L32

^13^C{^1^H} NMR (101 MHz, CDCl_3_) spectra of compound L32

**3-(tert-butyl)-5-((((1S,2S)-2-((3,5-di-tert-butyl-2-hydroxybenzyl)amino)cyclohexyl)amino)methyl)-4-hydroxyphenyl pivalate (L33)**

^1^H NMR (400 MHz, CDCl_3_) spectra of compound L33

^13^C{^1^H} NMR (101 MHz, CDCl_3_) spectra of compound L33

**2,4-di-tert-butyl-6-((((1S,2S)-2-((3-(tert-butyl)-5-((diphenylamino)methyl)-2-hydroxybenzyl)amino)cyclohexyl)amino)methyl)phenol (L34)**

^1^H NMR (400 MHz, CDCl_3_) spectra of compound L34

^13^C{^1^H} NMR (101 MHz, CDCl_3_) spectra of compound L34

**6,6'-((((1S,2S)-cyclohexane-1,2-diyl)bis(azanediyl))bis(methylene))bis(2,4-bis(2-phenylpropan-2-yl)phenol) (L35)**

^1^H NMR (400 MHz, CDCl_3_) spectra of compound L35

^13^C{^1^H} NMR (101 MHz, CDCl_3_) spectra of compound L35

**6,6'-((((1S,2S)-cyclohexane-1,2-diyl)bis(azanediyl))bis(methylene))bis(2-(tert-butyl)-4-methoxyphenol) (L36)**

^1^H NMR (400 MHz, CDCl_3_) spectra of compound L36

^13^C{^1^H} NMR (101 MHz, CDCl_3_) spectra of compound L36

**2-((((1S,2S)-2-((3-(tert-butyl)-2-hydroxybenzyl)amino)cyclohexyl)amino)methyl)-4,6-bis(2-phenylpropan-2-yl)phenol (L37)**

^1^H NMR (400 MHz, CDCl_3_) spectra of compound L37

^13^C{^1^H} NMR (101 MHz, CDCl_3_) spectra of compound L37

## NMR spectra of hydroxylation products

^1^H NMR (500 MHz, CDCl_3_) spectra of compound 2a

^13^C{^1^H} NMR (126 MHZ, CDCl_3_) spectra of compound 2a

^1^H NMR (400 MHz, CDCl_3_) spectra of compound 2b

^13^C{^1^H} NMR (101 MHZ, CDCl_3_) spectra of compound 2b

^1^H NMR (400 MHz, CDCl_3_) spectra of compound 2c

^13^C{^1^H} NMR (101 MHZ, CDCl_3_) spectra of compound 2c

^1^H NMR (400 MHz, CDCl_3_) spectra of compound 2d

^13^C{^1^H} NMR (101 MHZ, CDCl_3_) spectra of compound 2d

^1^H NMR (400 MHz, CDCl_3_) spectra of compound 2e

^13^C{^1^H} NMR (101 MHZ, CDCl_3_) spectra of compound 2e

^1^H NMR (400 MHz, CDCl_3_) spectra of compound 2f

^13^C{^1^H} NMR (101 MHZ, CDCl_3_) spectra of compound 2f

^1^H NMR (400 MHz, CDCl_3_) spectra of compound 2g

^13^C{^1^H} NMR (101 MHZ, CDCl_3_) spectra of compound 2g

^1^H NMR (400 MHz, CDCl_3_) spectra of compound 2h

^13^C{^1^H} NMR (101 MHZ, CDCl_3_) spectra of compound 2h

^1^H NMR (400 MHz, CDCl_3_) spectra of compound 2i

^13^C{^1^H} NMR (101 MHZ, CDCl_3_) spectra of compound 2i

^1^H NMR (400 MHz, CDCl_3_) spectra of compound 2j

^13^C{^1^H} NMR (101 MHZ, CDCl_3_) spectra of compound 2j

^1^H NMR (400 MHz, CDCl_3_) spectra of compound 2k

^13^C{^1^H} NMR (101 MHZ, CDCl_3_) spectra of compound 2k

^1^H NMR (400 MHz, CDCl_3_) spectra of compound 2l

^13^C{^1^H} NMR (101 MHZ, CDCl_3_) spectra of compound 2l

^1^H NMR (400 MHz, CDCl_3_) spectra of compound 2m

^13^C{^1^H} NMR (101 MHZ, CDCl_3_) spectra of compound 2m

^1^H NMR (400 MHz, CDCl_3_) spectra of compound 2n

^13^C{^1^H} NMR (101 MHZ, CDCl_3_) spectra of compound 2n

^1^H NMR (400 MHz, CDCl_3_) spectra of compound 2o

^13^C{^1^H} NMR (101 MHZ, CDCl_3_) spectra of compound 2o

^1^H NMR (400 MHz, CDCl_3_) spectra of compound 2p

^13^C{^1^H} NMR (101 MHZ, CDCl_3_) spectra of compound 2p

^1^H NMR (400 MHz, CDCl_3_) spectra of compound 2q

^13^C{^1^H} NMR (101 MHZ, CDCl_3_) spectra of compound 2q

^1^H NMR (400 MHz, CDCl_3_) spectra of compound 2r

^13^C{^1^H} NMR (101 MHZ, CDCl_3_) spectra of compound 2r

^1^H NMR (400 MHz, CDCl_3_) spectra of compound 2s

^13^C{^1^H} NMR (101 MHZ, CDCl_3_) spectra of compound 2s

^1^H NMR (400 MHz, CDCl_3_) spectra of compound 2t

^13^C{^1^H} NMR (101 MHZ, CDCl_3_) spectra of compound 2t

^1^H NMR (500 MHz, CDCl_3_) spectra of compound 2u

^13^C{^1^H} NMR (126 MHZ, CDCl_3_) spectra of compound 2u

^1^H NMR (400 MHz, CDCl_3_) spectra of compound 2v

^13^C{^1^H} NMR (101 MHZ, CDCl_3_) spectra of compound 2v

^1^H NMR (400 MHz, CDCl_3_) spectra of compound 2w

^13^C{^1^H} NMR (101 MHZ, CDCl_3_) spectra of compound 2w

^1^H NMR (400 MHz, CDCl_3_) spectra of compound 2x

^13^C{^1^H} NMR (101 MHZ, CDCl_3_) spectra of compound 2x

^1^H NMR (400 MHz, CDCl_3_) spectra of compound 2y

^13^C{^1^H} NMR (101 MHZ, CDCl_3_) spectra of compound 2y

^1^H NMR (400 MHz, CDCl_3_) spectra of compound 2z

^13^C{^1^H} NMR (101 MHZ, CDCl_3_) spectra of compound 2z

^1^H NMR (400 MHz, CDCl_3_) spectra of compound 2aa

^13^C{^1^H} NMR (101 MHZ, CDCl_3_) spectra of compound 2aa

^1^H NMR (400 MHz, CDCl_3_) spectra of compound 2ab

^13^C{^1^H} NMR (101 MHZ, CDCl_3_) spectra of compound 2ab

^1^H NMR (400 MHz, CDCl_3_) spectra of compound 2ac

^13^C{^1^H} NMR (101 MHZ, CDCl_3_) spectra of compound 2ac

^1^H NMR (400 MHz, CDCl_3_) spectra of compound 2ad

^13^C{^1^H} NMR (101 MHZ, CDCl_3_) spectra of compound 2ad

^1^H NMR (400 MHz, CDCl_3_) spectra of compound 2ae

^13^C{^1^H} NMR (101 MHZ, CDCl_3_) spectra of compound 2ae

^1^H NMR (400 MHz, CDCl_3_) spectra of compound 2af

^13^C{^1^H} NMR (101 MHZ, CDCl_3_) spectra of compound 2af

^1^H NMR (400 MHz, CDCl_3_) spectra of compound 2ag

^13^C{^1^H} NMR (101 MHZ, CDCl_3_) spectra of compound 2ag

^1^H NMR (500 MHz, DMSO-d_6_) spectra of compound 2ah

^13^C{^1^H} NMR (126 MHZ, DMSO-d_6_) spectra of compound 2ah

^1^H NMR (400 MHz, CDCl_3_) spectra of compound 2ai

^13^C{^1^H} NMR (101 MHZ, CDCl_3_) spectra of compound 2ai

^1^H NMR (400 MHz, CDCl_3_) spectra of compound 2aj

^13^C{^1^H} NMR (101 MHZ, CDCl_3_) spectra of compound 2aj

^1^H NMR (400 MHz, CDCl_3_) spectra of compound 2ak

^13^C{^1^H} NMR (101 MHZ, CDCl_3_) spectra of compound 2ak

^1^H NMR (400 MHz, CDCl_3_) spectra of compound 2al

^13^C{^1^H} NMR (101 MHZ, CDCl_3_) spectra of compound 2al

^1^H NMR (500 MHz, CDCl_3_) spectra of compound 2am

^13^C{^1^H} NMR (126 MHZ, CDCl_3_) spectra of compound 2am

^1^H NMR (400 MHz, CDCl_3_) spectra of compound 2an

^13^C{^1^H} NMR (101 MHZ, CDCl_3_) spectra of compound 2an

^13^C{^1^H} NMR (101 MHz, CDCl_3_) δ 193.69, 170.93, 145.61, 131.93, 130.59, 129.95, 129.83, 129.01, 77.54, 53.15, 32.45, 25.32.

^1^H NMR (400 MHz, CDCl_3_) spectra of compound 2ao

^13^C{^1^H} NMR (101 MHZ, CDCl_3_) spectra of compound 2ao

^19^F NMR (377 MHz, CDCl_3_) spectra of compound 2ao

^1^H NMR (400 MHz, CDCl_3_) spectra of compound 2ap

^13^C{^1^H} NMR (101 MHZ, CDCl_3_) spectra of compound 2ap

^1^H NMR (400 MHz, CDCl_3_) spectra of compound 2aq

^19^F NMR (377 MHz, CDCl_3_) spectra of compound 2aq

^13^C{^1^H} NMR (101 MHZ, CDCl_3_) spectra of compound 2aq

^1^H NMR (400 MHz, CDCl_3_) spectra of compound 2ar

^13^C{^1^H} NMR (101 MHZ, CDCl_3_) spectra of compound 2ar

^1^H NMR (500 MHz, CDCl_3_) spectra of compound 3

^13^C{^1^H} NMR (126 MHZ, CDCl_3_) spectra of compound 3

## HPLC Charts for **Hydroxylation** Products

**(S)-methyl 5-chloro-2-hydroxy-1-oxo-2,3-dihydro-1H-indene-2-carboxylate (2a)**

| **R. Time** | **Area** | **Area %** |
| --- | --- | --- |
| 19.645 | 18375618.000 | 99.6442 |
| 25.392 | 65611.594 | 0.3558 |

| **R. Time** | **Area** | **Area %** |
| --- | --- | --- |
| 19.317 | 35678844.000 | 50.7337 |
| 24.375 | 36646872.000 | 49.2663 |

**(S)-Methyl 5-bromo-2-hydroxy-1-oxo-2,3-dihydro-1H-indene-2-carboxylate (2b)**

| **R. Time** | **Area** | **Area %** |
| --- | --- | --- |
| 22.277 | 25805482.000 | 99.4065 |
| 27.732 | 154073.297 | 0.5935 |

| **R. Time** | **Area** | **Area %** |
| --- | --- | --- |
| 22.610 | 3438953.750 | 50.1247 |
| 27.623 | 3421843.500 | 49.8753 |

**(S)-Methyl 5-fluoro-2-hydroxy-1-oxo-2,3-dihydro-1H-indene-2-carboxylate (2c)**

| **R. Time** | **Area** | **Area %** |
| --- | --- | --- |
| 20.200 | 15260105.000 | 99.2217 |
| 26.742 | 119706.547 | 0.7783 |

| **R. Time** | **Area** | **Area %** |
| --- | --- | --- |
| 20.235 | 5285290.000 | 50.5922 |
| 26.422 | 5161553.500 | 49.4078 |

**(S)-Methyl 2-hydroxy-5-methyl-1-oxo-2,3-dihydro-1H-indene-2-carboxylate (2d)**

| **R. Time** | **Area** | **Area %** |
| --- | --- | --- |
| 18.248 | 19455548.000 | 99.2742 |
| 23.510 | 142237.656 | 0.7258 |

| **R. Time** | **Area** | **Area %** |
| --- | --- | --- |
| 18.323 | 5200637.500 | 45.2543 |
| 22.958 | 5358115.500 | 50.7457 |

**(S)-Methyl 2-hydroxy-5-methoxy-1-oxo-2,3-dihydro-1H-indene-2-carboxylate (2e)**

| **R. Time** | **Area** | **Area %** |
| --- | --- | --- |
| 19.412 | 23591598.000 | 98.7930 |
| 24.132 | 288231.688 | 1.2070 |

| **R. Time** | **Area** | **Area %** |
| --- | --- | --- |
| 19.822 | 6450387.000 | 50.3220 |
| 23.767 | 6367839.000 | 49.6780 |

**methyl (S)-2-hydroxy-1-oxo-2,3-dihydro-1H-indene-2-carboxylate (2f)**

| **R. Time** | **Area** | **Area %** |
| --- | --- | --- |
| 18.597 | 13720314.000 | 99.6684 |
| 23.542 | 45653.602 | 0.3316 |

| **R. Time** | **Area** | **Area %** |
| --- | --- | --- |
| 18.362 | 5848212.500 | 49.8976 |
| 22.750 | 5872220.000 | 50.1024 |

**(S)-Methyl 2-hydroxy-5,6-dimethoxy-1-oxo-2,3-dihydro-1H-indene-2-carboxylate (2g)**

| **R. Time** | **Area** | **Area %** |
| --- | --- | --- |
| 24.602 | 11941665.000 | 99.5338 |
| 29.650 | 55928.461 | 0.4662 |

| **R. Time** | **Area** | **Area %** |
| --- | --- | --- |
| 24.428 | 2958619.750 | 50.2881 |
| 29.603 | 2924714.500 | 49.7119 |

**methyl (S)-4-chloro-2-hydroxy-1-oxo-2,3-dihydro-1H-indene-2-carboxylate (2h)**

| **R. Time** | **Area** | **Area %** |
| --- | --- | --- |
| 26.655 | 20050268.000 | 99.1084 |
| 29.493 | 180369.500 | 0.8916 |

| **R. Time** | **Area** | **Area %** |
| --- | --- | --- |
| 26.480 | 3432733.500 | 50.0609 |
| 28.760 | 3424384.000 | 49.9391 |

**methyl (S)-4-bromo-2-hydroxy-1-oxo-2,3-dihydro-1H-indene-2-carboxylate (2i)**

| **R. Time** | **Area** | **Area %** |
| --- | --- | --- |
| 27.980 | 20736394.000 | 99.7697 |
| 30.888 | 47861.902 | 0.2303 |

| **R. Time** | **Area** | **Area %** |
| --- | --- | --- |
| 28.937 | 16661244.000 | 50.1104 |
| 31.215 | 16587829.000 | 49.8896 |

**methyl (S)-2-hydroxy-4-methyl-1-oxo-2,3-dihydro-1H-indene-2-carboxylate (2j)**

| **R. Time** | **Area** | **Area %** |
| --- | --- | --- |
| 16.120 | 16847660.000 | 99.3790 |
| 18.550 | 105279.656 | 0.6210 |

| **R. Time** | **Area** | **Area %** |
| --- | --- | --- |
| 16.258 | 13095738.000 | 50.0203 |
| 18.383 | 13085099.000 | 49.9797 |

**methyl (S)-2-hydroxy-4-methoxy-1-oxo-2,3-dihydro-1H-indene-2-carboxylate (2k)**

| **R. Time** | **Area** | **Area %** |
| --- | --- | --- |
| 25.318 | 84660.500 | 0.6715 |
| 26.753 | 12522335.000 | 99.3285 |

| **R. Time** | **Area** | **Area %** |
| --- | --- | --- |
| 24.693 | 5188951.000 | 49.7806 |
| 26.773 | 5234680.500 | 50.2194 |

**methyl (S)-2-hydroxy-6-methyl-1-oxo-2,3-dihydro-1H-indene-2-carboxylate (2l)**

| **R. Time** | **Area** | **Area %** |
| --- | --- | --- |
| 10.352 | 16292143.000 | 99.5829 |
| 12.497 | 68235.102 | 0.4171 |

| **R. Time** | **Area** | **Area %** |
| --- | --- | --- |
| 10.555 | 14194408.000 | 50.1858 |
| 12.405 | 14089310.000 | 49.8142 |

**methyl (S)-6-chloro-2-hydroxy-1-oxo-2,3-dihydro-1H-indene-2-carboxylate (2m)**

| **R. Time** | **Area** | **Area %** |
| --- | --- | --- |
| 11.835 | 9396613.000 | 98.6777 |
| 14.308 | 125915.492 | 1.3223 |

| **R. Time** | **Area** | **Area %** |
| --- | --- | --- |
| 12.198 | 2768353.000 | 52.0934 |
| 14.668 | 2545858.750 | 47.9066 |

**methyl (S)-6-bromo-2-hydroxy-1-oxo-2,3-dihydro-1H-indene-2-carboxylate (2n)**

| **R. Time** | **Area** | **Area %** |
| --- | --- | --- |
| 20.577 | 5349903.500 | 99.3480 |
| 26.217 | 35111.910 | 0.6520 |

| **R. Time** | **Area** | **Area %** |
| --- | --- | --- |
| 20.510 | 1806306.875 | 50.0959 |
| 25.845 | 1799392.750 | 49.9041 |

**methyl (S)-6-fluoro-2-hydroxy-1-oxo-2,3-dihydro-1H-indene-2-carboxylate (2o)**

| **R. Time** | **Area** | **Area %** |
| --- | --- | --- |
| 11.560 | 9538836.000 | 99.0258 |
| 14.167 | 93839.008 | 0.9742 |

| **R. Time** | **Area** | **Area %** |
| --- | --- | --- |
| 11.577 | 7539721.500 | 49.8725 |
| 14.037 | 7578284.500 | 50.1275 |

**methyl (S)-2-hydroxy-6-methoxy-1-oxo-2,3-dihydro-1H-indene-2-carboxylate (2p)**

| **R. Time** | **Area** | **Area %** |
| --- | --- | --- |
| 22.915 | 14521009.000 | 99.6929 |
| 28.263 | 44729.949 | 0.3071 |

| **R. Time** | **Area** | **Area %** |
| --- | --- | --- |
| 23.078 | 4944815.000 | 50.1270 |
| 27.887 | 4919751.500 | 49.8730 |

**methyl (S)-2-hydroxy-7-methoxy-1-oxo-2,3-dihydro-1H-indene-2-carboxylate (2q)**

| **R. Time** | **Area** | **Area %** |
| --- | --- | --- |
| 37.138 | 20803750.000 | 99.5836 |
| 46.032 | 86981.609 | 0.4164 |

| **R. Time** | **Area** | **Area %** |
| --- | --- | --- |
| 36.083 | 16686281.000 | 54.0667 |
| 42.827 | 14176094.000 | 45.9333 |

**ethyl (S)-2-hydroxy-1-oxo-2,3-dihydro-1H-indene-2-carboxylate(2r)**

| **R. Time** | **Area** | **Area %** |
| --- | --- | --- |
| 13.290 | 16458898.000 | 99.2530 |
| 15.783 | 123874.203 | 0.7470 |

| **R. Time** | **Area** | **Area %** |
| --- | --- | --- |
| 13.268 | 5532814.000 | 49.4724 |
| 15.517 | 5650827.000 | 50.5276 |

**ethyl (S)-5-chloro-2-hydroxy-1-oxo-2,3-dihydro-1H-indene-2-carboxylate (2s)**

| **R. Time** | **Area** | **Area %** |
| --- | --- | --- |
| 10.230 | 19200346.000 | 99.2548 |
| 12.655 | 144161.141 | 0.7452 |

| **R. Time** | **Area** | **Area %** |
| --- | --- | --- |
| 10.340 | 11691243.000 | 49.7938 |
| 12.777 | 11788073.000 | 50.2062 |

**ethyl (S)-2-hydroxy-5-methyl-1-oxo-2,3-dihydro-1H-indene-2-carboxylate (2t)**

| **R. Time** | **Area** | **Area %** |
| --- | --- | --- |
| 9.982 | 18736152.000 | 98.9733 |
| 12.437 | 194358.500 | 1.0267 |

| **R. Time** | **Area** | **Area %** |
| --- | --- | --- |
| 10.235 | 16521788.000 | 49.9916 |
| 12.622 | 16527307.000 | 50.0084 |

**ethyl (S)-2-hydroxy-6-methyl-1-oxo-2,3-dihydro-1H-indene-2-carboxylate (2u)**

| **R. Time** | **Area** | **Area %** |
| --- | --- | --- |
| 13.453 | 16051895.000 | 98.5904 |
| 16.270 | 229498.203 | 1.4096 |

| **R. Time** | **Area** | **Area %** |
| --- | --- | --- |
| 13.553 | 15535968.000 | 52.5302 |
| 16.147 | 14039345.000 | 47.4698 |

**ethyl (S)-4-chloro-2-hydroxy-1-oxo-2,3-dihydro-1H-indene-2-carboxylate (2v)**

| **R. Time** | **Area** | **Area %** |
| --- | --- | --- |
| 19.403 | 14867254.000 | 99.3087 |
| 21.660 | 103485.500 | 0.6913 |

| **R. Time** | **Area** | **Area %** |
| --- | --- | --- |
| 19.622 | 4950511.000 | 49.1501 |
| 21.613 | 5121725.000 | 50.8499 |

**ethyl (S)-4-bromo-2-hydroxy-1-oxo-2,3-dihydro-1H-indene-2-carboxylate (2w)**

| **R. Time** | **Area** | **Area %** |
| --- | --- | --- |
| 20.010 | 12314272.000 | 99.3297 |
| 22.058 | 83098.695 | 0.6703 |

| **R. Time** | **Area** | **Area %** |
| --- | --- | --- |
| 20.460 | 4455502.500 | 49.3895 |
| 22.110 | 4565644.000 | 50.6105 |

**ethyl (S)-2-hydroxy-4-methyl-1-oxo-2,3-dihydro-1H-indene-2-carboxylate (2x)**

| **R. Time** | **Area** | **Area %** |
| --- | --- | --- |
| 23.515 | 17301134.000 | 99.5679 |
| 28.175 | 75085.508 | 0.4321 |

| **R. Time** | **Area** | **Area %** |
| --- | --- | --- |
| 23.277 | 11973844.000 | 50.2069 |
| 27.155 | 11875135.000 | 49.7931 |

**ethyl (S)-2-hydroxy-6-methoxy-1-oxo-2,3-dihydro-1H-indene-2-carboxylate(2y)**

| **R. Time** | **Area** | **Area %** |
| --- | --- | --- |
| 11.772 | 6594527.500 | 99.0068 |
| 13.417 | 66151.406 | 0.9932 |

| **R. Time** | **Area** | **Area %** |
| --- | --- | --- |
| 11.875 | 7100174.000 | 49.7429 |
| 13.482 | 7173570.500 | 50.2571 |

**isopropyl (S)-2-hydroxy-1-oxo-2,3-dihydro-1H-indene-2-carboxylate (2z)**

| **R. Time** | **Area** | **Area %** |
| --- | --- | --- |
| 7.852 | 8704854.000 | 99.4310 |
| 8.685 | 49812.008 | 0.5690 |

| **R. Time** | **Area** | **Area %** |
| --- | --- | --- |
| 7.733 | 9555157.000 | 51.7815 |
| 8.493 | 8897668.000 | 48.2185 |

**isopropyl (S)-5-chloro-2-hydroxy-1-oxo-2,3-dihydro-1H-indene-2-carboxylate (2aa)**

| **R. Time** | **Area** | **Area %** |
| --- | --- | --- |
| 11.510 | 12130260.000 | 99.3970 |
| 13.985 | 73583.398 | 0.6030 |

| **R. Time** | **Area** | **Area %** |
| --- | --- | --- |
| 11.652 | 8166759.000 | 49.8778 |
| 14.110 | 8206764.500 | 50.1222 |

**benzyl (S)-5-chloro-2-hydroxy-1-oxo-2,3-dihydro-1H-indene-2-carboxylate (2ab)**

| **R. Time** | **Area** | **Area %** |
| --- | --- | --- |
| 26.618 | 33432668.000 | 99.2594 |
| 34.592 | 249458.906 | 0.7406 |

| **R. Time** | **Area** | **Area %** |
| --- | --- | --- |
| 26.808 | 18007980.000 | 49.9844 |
| 34.093 | 18019206.000 | 50.0156 |

**benzyl (S)-2-hydroxy-1-oxo-2,3-dihydro-1H-indene-2-carboxylate (2ac)**

| **R. Time** | **Area** | **Area %** |
| --- | --- | --- |
| 24.028 | 23954396.000 | 99.2770 |
| 30.157 | 174459.047 | 0.7230 |

| **R. Time** | **Area** | **Area %** |
| --- | --- | --- |
| 23.977 | 8198957.000 | 50.2403 |
| 29.377 | 8120530.500 | 49.7597 |

**tert-butyl (S)-5-chloro-2-hydroxy-1-oxo-2,3-dihydro-1H-indene-2-carboxylate (2ad)**

| **R. Time** | **Area** | **Area %** |
| --- | --- | --- |
| 8.173 | 14247679.000 | 99.3439 |
| 8.992 | 94090.203 | 0.6561 |

| **R. Time** | **Area** | **Area %** |
| --- | --- | --- |
| 8.448 | 17417896.000 | 49.3658 |
| 9.298 | 17865412.000 | 50.6342 |

**(3S)-adamantan-1-yl (2S)-5-chloro-2-hydroxy-1-oxo-2,3-dihydro-1H-indene-2-carboxylate (2ae)**

| **R. Time** | **Area** | **Area %** |
| --- | --- | --- |
| 17.553 | 24106248.000 | 97.8326 |
| 29.010 | 534044.813 | 2.1674 |

| **R. Time** | **Area** | **Area %** |
| --- | --- | --- |
| 17.615 | 14346540.000 | 49.8908 |
| 29.075 | 14409356.000 | 50.1092 |

**(3S)-adamantan-1-yl(2S)-2-hydroxy-4-methoxy-1-oxo-2,3-dihydro-1H-indene-2-carboxylate (2af)**

| **R. Time** | **Area** | **Area %** |
| --- | --- | --- |
| 24.633 | 10842725.000 | 98.8656 |
| 33.795 | 124411.406 | 1.1344 |

| **R. Time** | **Area** | **Area %** |
| --- | --- | --- |
| 24.653 | 5029230.000 | 53.4641 |
| 36.053 | 4377508.500 | 46.5359 |

**(3S)-adamantan-1-yl (2S)-2-hydroxy-1-oxo-2,3-dihydro-1H-indene-2-carboxylate (2ag)**

| **R. Time** | **Area** | **Area %** |
| --- | --- | --- |
| 16.303 | 9016080.000 | 94.7676 |
| 26.235 | 497803.719 | 5.2324 |

| **R. Time** | **Area** | **Area %** |
| --- | --- | --- |
| 16.303 | 3614286.000 | 49.8632 |
| 26.118 | 3634117.750 | 50.1368 |

**(S)-5-chloro-2-hydroxy-1-oxo-N-phenyl-2,3-dihydro-1H-indene-2-carboxamide (2ah)**

| **R. Time** | **Area** | **Area %** |
| --- | --- | --- |
| 24.753 | 19257062.000 | 95.0626 |
| 41.315 | 1000181.250 | 4.9374 |

| **R. Time** | **Area** | **Area %** |
| --- | --- | --- |
| 25.790 | 4814540.500 | 49.9838 |
| 42.053 | 4817655.000 | 50.0162 |

**(S)-N-(tert-butyl)-5-chloro-2-hydroxy-1-oxo-2,3-dihydro-1H-indene-2-carboxamide (2ai)**

| **R. Time** | **Area** | **Area %** |
| --- | --- | --- |
| 7.365 | 3668235.250 | 14.1862 |
| 26.137 | 22189622.000 | 85.8139 |

| **R. Time** | **Area** | **Area %** |
| --- | --- | --- |
| 7.363 | 3840346.250 | 46.9942 |
| 26.565 | 4331607.500 | 53.0058 |

**(S)-5-chloro-2-hydroxy-N-isopropyl-1-oxo-2,3-dihydro-1H-indene-2-carboxamide (2aj)**

| **R. Time** | **Area** | **Area %** |
| --- | --- | --- |
| 8.383 | 948596.563 | 13.2831 |
| 9.232 | 6192761.500 | 86.7169 |

| **R. Time** | **Area** | **Area %** |
| --- | --- | --- |
| 8.270 | 10766026.000 | 50.7908 |
| 9.180 | 10430779.000 | 49.2092 |

**(S)-N-butyl-5-chloro-2-hydroxy-1-oxo-2,3-dihydro-1H-indene-2-carboxamide (2ak)**

| **R. Time** | **Area** | **Area %** |
| --- | --- | --- |
| 11.435 | 5062128.000 | 20.4609 |
| 12.933 | 19678408.000 | 79.5391 |

| **R. Time** | **Area** | **Area %** |
| --- | --- | --- |
| 11.457 | 5863991.500 | 52.6665 |
| 13.138 | 5270199.000 | 47.3335 |

**methyl (S)-2-hydroxy-1-oxo-1,2,3,4-tetrahydronaphthalene-2-carboxylate (2al)**

| **R. Time** | **Area** | **Area %** |
| --- | --- | --- |
| 20.472 | 7731463.500 | 90.6170 |
| 23.932 | 800558.563 | 9.3830 |

| **R. Time** | **Area** | **Area %** |
| --- | --- | --- |
| 20.635 | 5565916.000 | 47.5715 |
| 23.975 | 6134184.500 | 52.4285 |

**ethyl (S)-2-hydroxy-1-oxo-1,2,3,4-tetrahydronaphthalene-2-carboxylate (2am)**

| **R. Time** | **Area** | **Area %** |
| --- | --- | --- |
| 15.528 | 6588262.000 | 90.3097 |
| 17.693 | 706928.313 | 9.6903 |

| **R. Time** | **Area** | **Area %** |
| --- | --- | --- |
| 15.592 | 8751661.000 | 50.0591 |
| 17.710 | 8730991.000 | 49.9409 |

**methyl (S)-6-bromo-2-hydroxy-1-oxo-1,2,3,4-tetrahydronaphthalene-2-carboxylate (2an)**

| **R. Time** | **Area** | **Area %** |
| --- | --- | --- |
| 23.590 | 12820399.000 | 90.4216 |
| 26.753 | 1358071.875 | 9.5784 |

| **R. Time** | **Area** | **Area %** |
| --- | --- | --- |
| 23.907 | 26749752.000 | 50.1203 |
| 26.970 | 26621364.000 | 49.8797 |

**methyl (S)-6-fluoro-2-hydroxy-1-oxo-1,2,3,4-tetrahydronaphthalene-2-carboxylate (2ao)**

| **R. Time** | **Area** | **Area %** |
| --- | --- | --- |
| 21.522 | 5564221.000 | 90.4394 |
| 25.157 | 588210.063 | 9.5606 |

| **R. Time** | **Area** | **Area %** |
| --- | --- | --- |
| 21.668 | 12756600.000 | 50.1739 |
| 25.280 | 12668161.000 | 49.8261 |

**methyl (S)-7-bromo-2-hydroxy-1-oxo-1,2,3,4-tetrahydronaphthalene-2-carboxylate (2ap)**

| **R. Time** | **Area** | **Area %** |
| --- | --- | --- |
| 21.338 | 13043748.000 | 95.6315 |
| 24.002 | 595850.188 | 4.3585 |

| **R. Time** | **Area** | **Area %** |
| --- | --- | --- |
| 21.712 | 7450994.500 | 50.0086 |
| 24.282 | 7448419.000 | 49.9914 |

**methyl (S)-7-fluoro-2-hydroxy-1-oxo-1,2,3,4-tetrahydronaphthalene-2-carboxylate (2aq)**

| **R. Time** | **Area** | **Area %** |
| --- | --- | --- |
| 18.897 | 7137056.000 | 90.5343 |
| 20.452 | 746201.500 | 9.4656 |

| **R. Time** | **Area** | **Area %** |
| --- | --- | --- |
| 19.525 | 4184983.250 | 49.3612 |
| 21.018 | 4293298.000 | 50.6388 |

**methyl (S)-5-bromo-2-hydroxy-1-oxo-1,2,3,4-tetrahydronaphthalene-2-carboxylate (2ar)**

| **R. Time** | **Area** | **Area %** |
| --- | --- | --- |
| 19.627 | 5060842.500 | 91.1054 |
| 26.690 | 494086.500 | 8.8946 |

| **R. Time** | **Area** | **Area %** |
| --- | --- | --- |
| 19.737 | 24843682.000 | 49.1503 |
| 26.957 | 25702708.000 | 50.8497 |

**(S)-5-chloro-1-oxo-2-(2-phenylacetoxy)-2,3-dihydro-1H-indene-2-carboxylate (3)**

| **R. Time** | **Area** | **Area %** |
| --- | --- | --- |
| 18.305 | 36127.902 | 0.0587 |
| 26.430 | 62552200.000 | 99.9413 |

| **R. Time** | **Area** | **Area %** |
| --- | --- | --- |
| 17.880 | 28562598.000 | 49.5656 |
| 28.012 | 29063252.000 | 50.4344 |
